# Anti-*Trypanosoma cruzi* Effects of Sesquiterpenoids from Branches of *Drimys brasiliensis* (Winteraceae)

**DOI:** 10.1021/acsomega.5c08083

**Published:** 2025-11-03

**Authors:** Eric Umehara, Dayana A. S Ferreira, Mariana B. Abiuzi, Myron Christodoulides, Ravi Kant, Andre G. Tempone, João Henrique G. Lago

**Affiliations:** † Center for Natural and Human Sciences, Federal University of the ABC, Santo Andre, Sao Paulo 09210-580, Brazil; ‡ Laboratory of Pathophysiology, 196591 Instituto Butantan, 1500, São Paulo, Sao Paulo 05503-900, Brazil; § Molecular Microbiology, School of Clinical and Experimental Sciences, Faculty of Medicine, 7423 University of Southampton, Southampton SO166YD, U.K.

## Abstract

The hexane extract of *Drimys brasiliensis* (Winteraceae) branches displayed activity against trypomastigote forms of *Trypanosoma cruzi* and was subjected to a bioactivity-guided fractionation procedures to afford four bisabolene—*rel*-(3*S*,7*R*)-3-hydroxy-bisabola-1­(6),10-dien-2-one (**1**), *rel*-(2*S*,3*S*,7*R*)-bisabola-1­(6),10-diene-2,3-diol (**2**), *rel*-(3*S*,6*R*,7*R*)-3,6-epidioxy-1,10-bisaboladiene (**3**), and α-curcumene (**4**)—as well as two drimane—polygodial (**5**), and 9-deoxymuzigadial (**6**)—type sesquiterpenes. This is the first report of compounds **1** and **2** as natural products. Compounds **1–6** showed promising activity against trypomastigotes and intracellular amastigotes with EC_50_ values ranging from 3.8 to 88.3 μM and 3.1 to 24.2 μM, respectively. Furthermore, the isolated compounds displayed no cytotoxicity for mammalian fibroblasts (CC_50_ > 200 μM), demonstrating a safety profile. *In silico* studies of bioactive sesquiterpenes showed adequate predictions for drug-like properties, with adherence to Lipinski′s rules of five (RO5), acceptable ADMET properties and no similarities to interference compounds (PAINS). Finally, an *in silico* molecular docking exercise suggested that the more active compounds **5** and **6** could interact favorably with the *T. cruzi* mitochondrial ATP synthase protein. Altogether, these findings highlight the potential of bisabolane and drimane type sesquiterpenoids isolated from branches of *D. brasiliensis* as new hit compounds and contribute to the ongoing search for novel molecular scaffolds for the treatment of a neglected tropical disease such as Chagas disease.

## Graphical abstract

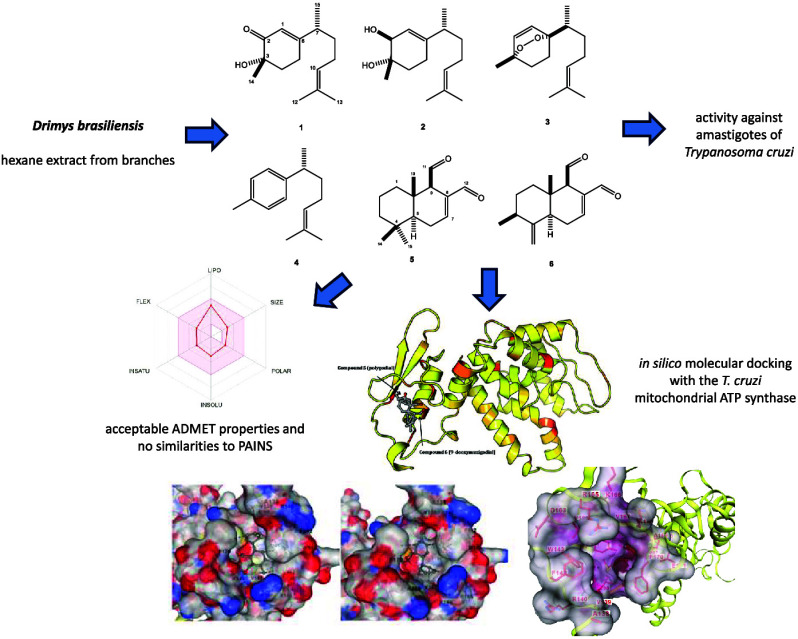

## 1 Introduction

Neglected Tropical Diseases (NTDs) comprise a group of approximately 21 infectious diseases caused by diverse pathogens, which collectively exert substantial health, social, and economic burdens on affected regions. These diseases predominantly impact populations in tropical and subtropical areas and are estimated to affect over one billion individuals worldwide.^1^ The global health impact of NTDs is often underestimated, especially with Chagas disease (CD) due to the long asymptomatic period, resulting in no diagnostics.^2–4^ Chagas disease—caused by the protozoan parasite *Trypanosoma cruzi*—stands out, affecting more than seven million people mainly in the Americas.^5^ Current chemotherapeutic options, primarily nitro-derivatives such as benznidazole and nifurtimox, are limited by high toxicity and reduced efficacy, particularly during the acute phase of the disease.^3,4^ In this context, the identification of natural products with potent activity against trypomastigote and amastigote stages of the parasite represents a promising strategy for the development of novel antitrypanosomal agents.^5^

*Drimys brasiliensis* Miers. (Winteraceae), commonly referred to as “casca de anta” or “cataia”, has gained recognition as a species of pharmacological interest due to its demonstrated therapeutic potential. Previous investigations have reported its antifungal,^6^ antiparasitic,^7–10^ cytotoxic,^11,12^ and anti-inflammatory^13,14^ activities. Phytochemical analyses have identified several drimane-type sesquiterpenes in this species,^15^ including polygodial and *epi*-polygodial, both exhibit antiparasitic activity against *T. cruzi*.^7,10^ As part of ongoing research on *D. brasiliensis*,^16^ the hexane extract derived from its branches was found to be active against the trypomastigote forms of *T. cruzi*. Six sesquiterpenes (**1–6**) were isolated through a bioactivity-guided fractionation strategy, and their activity against trypomastigotes and intracellular amastigotes of *T. cruzi*, as well as their cytotoxicity toward NCTC mammalian cells, was evaluated *in vitro*.

## 2 Results and Discussion

The hexane extract from the branches of *D. brasiliensis* displayed activity against trypomastigote forms of *T. cruzi* (100% of parasite death at 200 μg/mL). Based on this result, this extract was subjected to a bioactivity-guided fractionation to afford four bisabolane (**1–4**) and two drimane (**5** and **6**) type sesquiterpenes, as showed in Figure 1.

**Figure 1:**
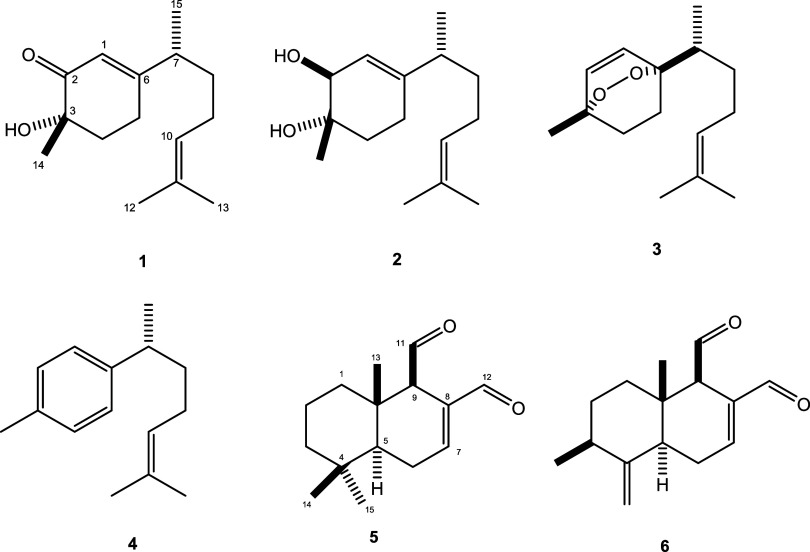
Structures of bisabolane (**1–4**) and drimane (**5** and **6**) type sesquiterpenes.

Compound **1** was isolated as a white amorphous powder, and its molecular formula, C_15_H_24_O_2_, was established based on ESI-HRMS, which displayed a [M + H]^+^ ion at *m*/*z* 237.1843 (calculated for C_15_H_25_O_2_, 237.1854). The ^1^H NMR spectrum of compound **1** showed the characteristic signals of double bonds at δ 5.89 (s, H-1) and 5.05 (t, *J* = 7.0 Hz, H-10) as well as four signals at δ 1.68 (s, H-12), 1.57 (s, H-13), 1.30 (s, H-14), and 1.10 (d, *J* = 6.9 Hz, H-15), assigned to methyl groups. ^13^C and DEPT NMR spectra revealed 15 signals, including those assigned to an α,β-unsaturated carbonyl system at δ 202.8 (C-2), 171.7 (C-6) and 121.8 (C-1). One carbinol carbon peak was observed at δ 72.8 (C-3) whereas four methyl signals were detected at δ 25.9 (C-12), 24.3 (C-14), 19.3 (C-15), and 17.8 (C-13). Additional methylene and methine carbons were detected as a set of signals between δ 35.8 and 25.9. Based on the correlations observed in the HMBC spectrum between H-1 and C-3/C-7, H-15 and C-7/C-6, and H-14 and C-3/C-2, the carbonyl and hydroxyl groups were positioned at C-2 and C-3, respectively. Additionally, the relative configuration of C-3 was suggested based on the correlations between H-4_eq_ and H-14, as well as between H-4_eq_ and H-5_ax_, observed in the NOESY spectrum. As a result, compound **1** was named *rel*-(3*S*,7*R*)-3-hydroxy-bisabola-1­(6),10-dien-2-one (Figure 1), previously reported as a synthetic product.^17^

Compound **2** was likewise obtained as a white amorphous powder, and its molecular formula, C_15_H_26_O_2_, was determined ESI-HRMS, which exhibited a [M + Na]^+^ ion peak at *m*/*z* 261.1812 (calculated for C_15_H_26_O_2_Na, 261.1830). Similarly to compound **1**, the ^1^H NMR spectrum of **2** exhibited characteristic signals corresponding to double bonds at δ 5.35 (br s, H-1) and at δ 5.09 (br t, *J* = 7.5 Hz, H-10), along with four methyl group resonances at δ 1.67 (s, H-12), 1.58 (s, H-13), 1.20 (s, H-14), and 1.00 (d, *J* = 6.5 Hz, H-15). Additionally, a signal at δ 4.00 (br s) suggested the presence of an oxymethine hydrogen, assigned to H-2. The ^13^C and DEPT NMR spectra of compound **2** revealed 15 carbon signals, including sp^2^-hybridized carbons at δ 122.3 (C-1), 145.0 (C-6), 124.4 (C-10), and 131.4 (C-11), as well as two carbinol carbons at δ 74.7 (C-2) and 72.2 (C-3). The absence of a carbonyl signal, combined with these data, supports the identification of compound **2** as the 2,3-diol derivative of compound **1**. Key HMBC correlations were observed between H-1 and C-3/C-7, and between H-4 and C-2/C-3/C-6, confirming the proposed carbon framework. Additionally, NOESY correlations between H-14 and H-4_eq_, and between H-4_eq_ and H-5_ax_, as well as the absence of correlation between H-2 and H-14, suggest a *trans* configuration of the hydroxyl groups at C-2 and C-3, positioning the methyl group (C-14) at axial, similarly as observed to compound **1**. Therefore, this compound was named *rel*-(2*S*,3*S*,7*R*)-bisabola-1­(6),10-diene-2,3-diol (Figure 1), previously reported as an oxidation product of γ-curcumene.^17^

Structures of *rel*-(3*S*,6*R*,7*R*)-3,6-epidioxy-1,10-bisaboladiene (**3**), α-curcumene (**4**), polygodial (**5**), and 9-deoxymuzigadial (**6**) were proposed by analysis of NMR and ESI-HRMS spectral data followed by comparison with those published in the literature.^16,18,19^ While compounds **3–6** have been previously isolated from *D. brasiliensis*,^7,12–16^ this study represents the first report of compounds **1** and **2** as naturally occurring products.

Compounds **1–6** were evaluated *in vitro* for their activity against both the trypomastigote and intracellular amastigote forms of *T. cruzi* (Table 1).

**Table 1:** Anti-*T. cruzi* Activity (Trypomastigote and Amastigote Forms) and Mammalian Cytotoxicity (NCTC Cells) of Compounds **1–6** and Standard Drug^a^

	EC_50_/μM		CC_50_/μM	SI	
compounds	trypomastigote	amastigote	NCTC	trypomastigote	amastigote
**1**	38.5 ± 2.7	6.7 ± 3.8	>200	>5.2	>29.7
**2**	88.3 ± 6.5	24.2 ± 4.4	>200	>2.3	>8.3
**3**	65.5 ± 5.4	NA	>200	>3.0	
**4**	20.5 ± 1.6	4.9 ± 0.1	>200	>9.0	>41.1
**5**	3.8 ± 3.1	3.5 ± 0.2	>200	>52.0	>57.3
**6**	5.9 ± 0.9	3.1 ± 0.1	>200	>34.0	>64.3
**benznidazole**	17.4 ± 1.8	5.5 ± 2.2	>200	>13.0	>36.4

a NA: nonactive; SD: Standard deviation; EC_50_: 50% Effective concentration; CC_50_: 50% Cytotoxic Concentration; SI: Selectivity Index.

Among them, compounds **5** and **6** exhibited the highest potency against trypomastigotes, with EC_50_ values of 3.8 and 5.9 μM, respectively. In comparing the antiparasitic activity of the bisabolane derivatives (**1**–**4**) against the extracellular forms, the data suggest that aromatization of the C6 ring may play an important role in enhancing activity, as compounds **1**–**3** demonstrated limited efficacy, whereas compound **4** exhibited an improved potency with an EC_50_ value of 20.5 μM. When evaluated against intracellular forms of *T. cruzi*, compounds **1**, **2**, **4**–**6** exhibited activities with EC_50_ values ranging from 3.1 to 24.2 μM. Among the bisabolane derivatives **1** and **2**, the presence of a carbonyl group at C-2 appears to be a key structural feature influencing activity, as evidenced by the EC_50_ values determined to be 6.7 and 24.2 μM for compounds **1** and **2**, respectively. Additionally, compound **4** demonstrated an EC_50_ of 4.9 μM, underscoring the importance of C6 ring aromatization, a structural trait also associated with activity against trypomastigote forms. In contrast, compound **3**, which contains an endoperoxide functional group in its structure, exhibited no significant antiparasitic activity. Regarding the drimane-type derivatives **5** and **6**, which differ in their substituents at C-3 and C-4, both exhibited high potency against amastigotes, with EC_50_ values of 3.5 and 3.1 μM, respectively. While the activity of compound **5** against *T. cruzi* trypomastigotes has been previously reported,^7^ this study represents the first report of its efficacy against the intracellular forms of the parasite. Notably, all compounds (**1**–**6**) showed no cytotoxicity toward NCTC mammalian cells to the highest tested concentration (CC_50_ > 200 μM).

The Drugs for Neglected Diseases initiative (DNDi) establishes specific criteria for the selection of novel hit compounds against Chagas disease, including an inhibitory concentration (EC_50_) below 10 μM for intracellular *T. cruzi* and selectivity index (SI) higher than 10.^20^ In accordance to these criteria, compounds **1**, **4**–**6** meets these requirements, exhibiting notably SI values −29.7, 41.1, 57.3 and 64.3, respectively.

To assess the potential of sesquiterpenes **1**–**6** as novel hit compounds against *T. cruzi*, an *in silico* analysis was conducted using the SwissADME platform to evaluate their physicochemical properties, pharmacokinetic parameters, and drug-likeness^21^ (Figure 2).

**Figure 2:**
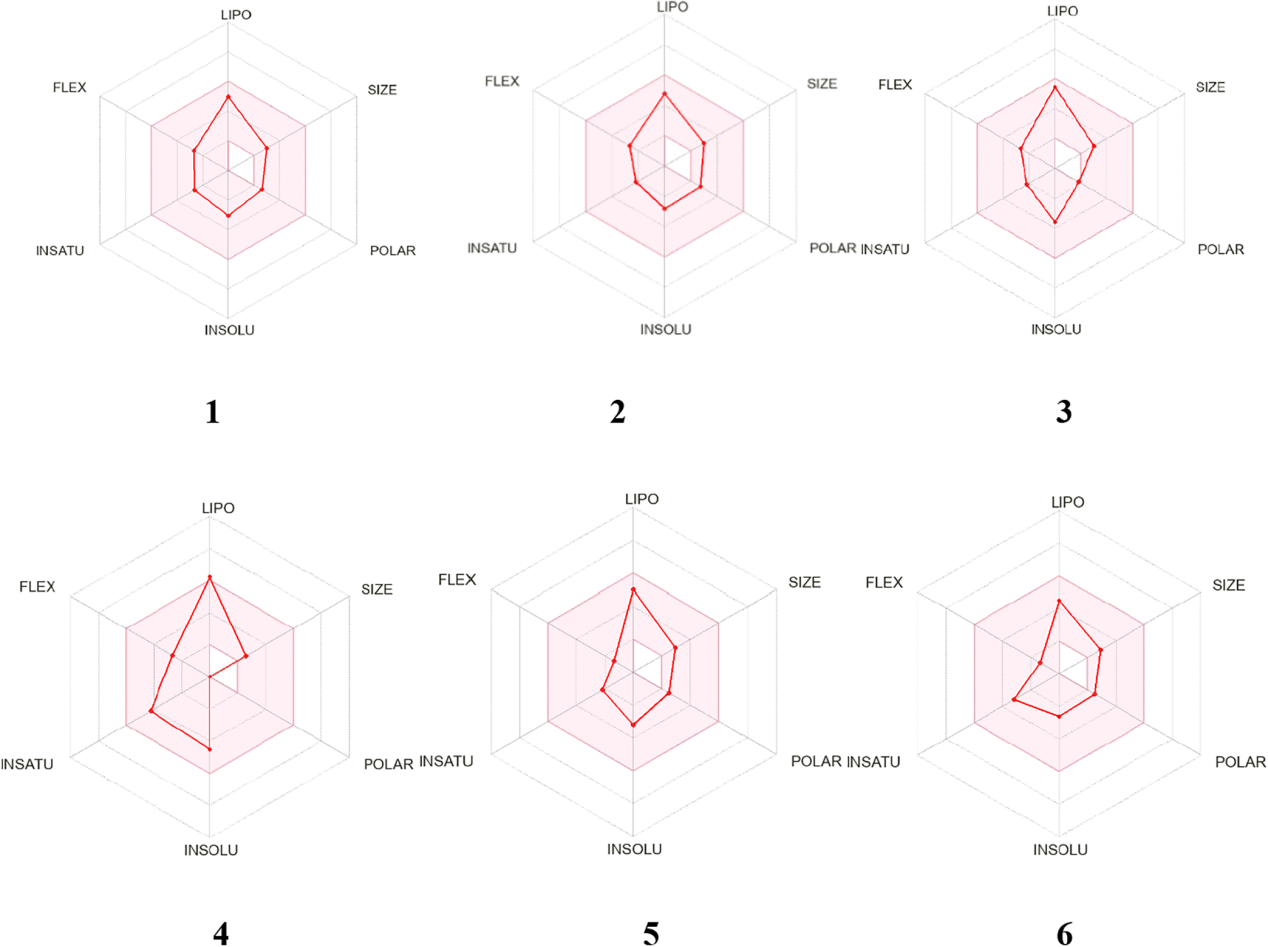
*In silico* study for drug-likeness using the SwissADME tool of compounds **1**–**6**. Red area represents the optimal range for each property including lipophilicity (XLOGP3), size (MW), polarity (TPSA), solubility (log *S*), saturation (fraction of carbons in the sp^3^ hybridization) and flexibility (rotatable bonds).

The initial screening via the Bioavailability Radar indicated favorable alignment of compounds **1**–**3**, **5** and **6** with all evaluated criteria, including lipophilicity (XLOGP3), size (MW), polarity (TPSA), solubility (log *S*), saturation (fraction of carbons in the sp3 hybridization) and flexibility (rotatable bonds), as indicated in Table 2.

**Table 2:** *In Silico* Physicochemical Properties and ADMET Predictions for Compounds **1**–**6**

parameters	**1**	**2**	**3**	**4**	**5**	**6**
	Physicochemical Properties					
molecular weight	236.35	238.37	236.35	202.34	234.33	234.33
num. heavy atoms	17	17	17	15	17	17
fraction Csp^3^	0.67	0.73	0.73	0.47	0.73	0.60
num. rotatable bonds	4	4	4	4	2	2
num. H-donnors	1	2	0	0	0	0
num. H-acceptors	2	2	2	0	2	2
molar refractivity	72.56	73.52	71.29	69.55	69.40	69.18
TPSA/Å^2^ ^a^	37.30	40.46	18.46	0	34.14	34.14

	Lipofilicity^b^					
log *P*_o/w_ ^b^	3.19	2.95	3.79	4.86	3.23	3.38

	Water Solubility^c^					
log *S*	–3.04	–2.84	–3.59	–4.52	–3.20	–2.63
class	S	S	S	MS	S	S

	Pharmacokinetics					
GI absorption	high	high	high	low	high	high
BBB permeant	yes	yes	yes	no	yes	yes
P-gp substrate	no	no	no	no	no	no
CYP1A2 inhibitor	no	no	no	no	no	no
CYP2C19 inhibitor	no	no	yes	no	no	no
CYP2C9 inhibitor	no	no	yes	no	no	no
CYP2D6 inhibitor	no	no	no	yes	no	no
CYP3A4 inhibitor	no	no	no	no	no	no

	Drug-likeness					
Lipinksi^d^	yes	yes	yes	no	yes	yes
Ghose^e^	yes	yes	yes	yes	yes	yes
Veber^f^	yes	yes	yes	yes	yes	yes
Egan^g^	yes	yes	yes	yes	yes	yes

	Medicinal Chemistry					
PAINS	0 alert	0 alert	0 alert	0 alert	0 alert	0 alert

a TPSA: topological polar surface area.

b log *P*_o/w_ = partition coefficient between *n*-octanol and water;

c log *S*/Class: insoluble (I) < −10 < poor soluble (PS) < −6 < moderately soluble (MS) < −4 < soluble (S) < −2 < very soluble (VS) < 0 < highly soluble (HS);

d Lipinski = MW ≤ 500; log *P*_o/w_ ≤ 5; H-bond donors ≤ 5; H-bond acceptors ≤ 10;

e Ghose = 180 ≤ MW ≤ 480; 20 ≤ No. of atoms ≤ 70; 40 ≤ Molar Refractivity ≤ 130; −0.4 ≤ log *P*_o/w_ ≤ 5.6;

f Veber = Num. Rotatable Bonds ≤ 10; TPSA ≤ 140 Å^2^;

g Egan = log *P*_o/w_ ≤ 5.88; TPSA ≤ 131.6 Å^2^.

Physicochemical analysis revealed adequate solubility profiles for compounds **1**, **5** and **6**, with log *P*_o/w_ values of 3.19, 3.23, and 3.38, respectively, suggesting appropriate lipophilicity for oral bioavailability. Furthermore, prediction of interactions with key cytochrome P450 isoenzymes—responsible for xenobiotic metabolism—indicated low promiscuity for these compounds. In terms of drug-likeness, compounds **1**, **5**, and **6** complied with multiple pharmaceutical filters, including those proposed by Ghose, Veber, Egan, and Muegge, and did not violate Lipinski’s Rule of Five. Notably, no Pan Assay Interference Compounds (PAINS) alerts were detected, supporting the suitability of these sesquiterpenes as promising candidates for further optimization in Chagas disease drug discovery.

Compounds **5** and **6** appeared to be the most promising hit compounds with respect to biological activity against trypomastigote and amastigote forms. The exact mechanisms of action of these compounds against the parasite are not known, but several hypotheses can be constructed from studies on other microorganisms. Activity could be multifunctional, e.g., in fungi, polygodial has been shown to act as a membrane surfactant, induce reactive oxygen species and inhibit mitochondrial F_0_F_1_ ATPase.^19,22,23^ Unlike polygodial, the mechanism of action of 9-deoxymuzigadial is unknown, but the presence of the reactive α,β-unsaturated dialdehyde moiety common to drimane sesquiterpenes, could enable it to potentially form covalent adducts (Schiff bases or Michael additions) with nucleophiles like lysine/cysteine residues in proteins or amines in membrane lipids.^24^ Based on these studies, we hypothesized that the mitochondrial ATP synthase could be a promising and specific intracellular protein target for these two structurally related compounds. We thus did a protein modeling docking study of the *T. cruzi* mitochondrial ATP synthase and polygodial and 9-deoxymuzigadial.

Initially, the homology model of the *T. cruzi* mitochondrial ATP synthase β-subunit was generated using the AlphaFold server. The predicted structure displayed a well-defined globular architecture consistent with known ATP synthase β-subunit folds (Figure 3).

**Figure 3:**
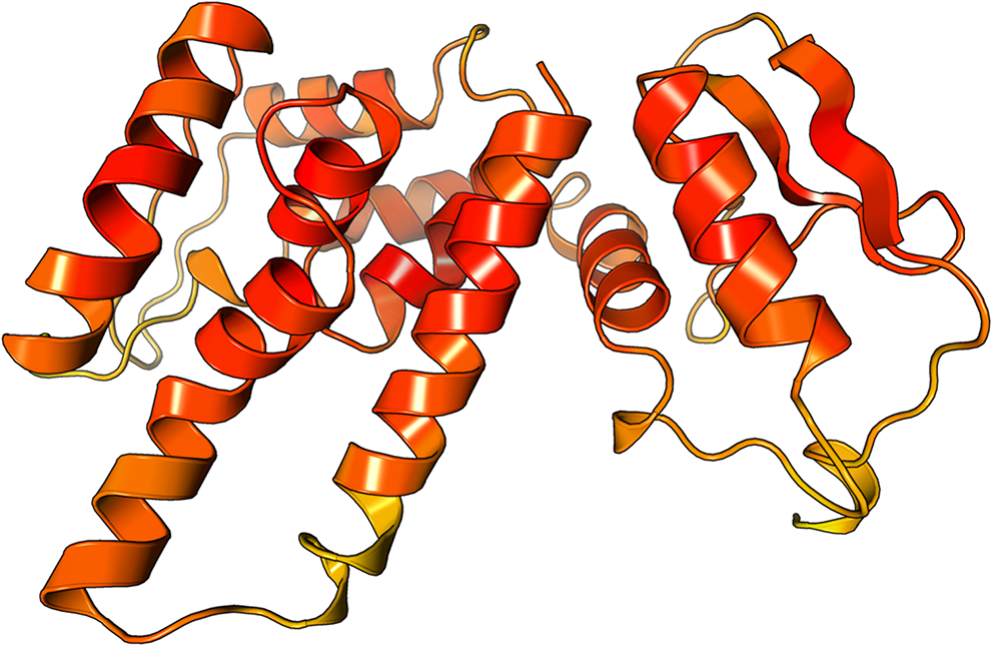
Predicted structure of the *T. cruzi* ATP synthase β-subunit modeled using AlphaFold. The model shows a typical globular fold with well-defined α-helices and high per-residue confidence across conserved catalytic regions.

Confidence scores (pLDDT) were uniformly high across most regions, particularly in conserved α-helical domains forming the core of the catalytic site. For validation of the modeled structure, the ProSA-web analysis yielded a *Z*-score of −6.82, which was well within the range typically observed for native protein structures of similar length, supporting the model’s reliability (Figure S43—Supporting Information). Structural superimposition with the *Trypanosoma brucei* ATP synthase (8AP6) revealed a close alignment, with a root-mean-square deviation (RMSD) of 1.89A0, which indicated strong structural conservation. The secondary structure elements and functionally relevant regions showed positional agreement, reinforcing the biological plausibility of the modeled conformation. Next, PocketFinder identified the potential binding pocket that was the top-ranked site defined by depth and accessible surface area and this was selected for docking (Figure 4). This cavity was located near a conserved region of the catalytic core, adjacent to residues known to participate in nucleotide binding in homologous ATP synthase structures.

**Figure 4:**
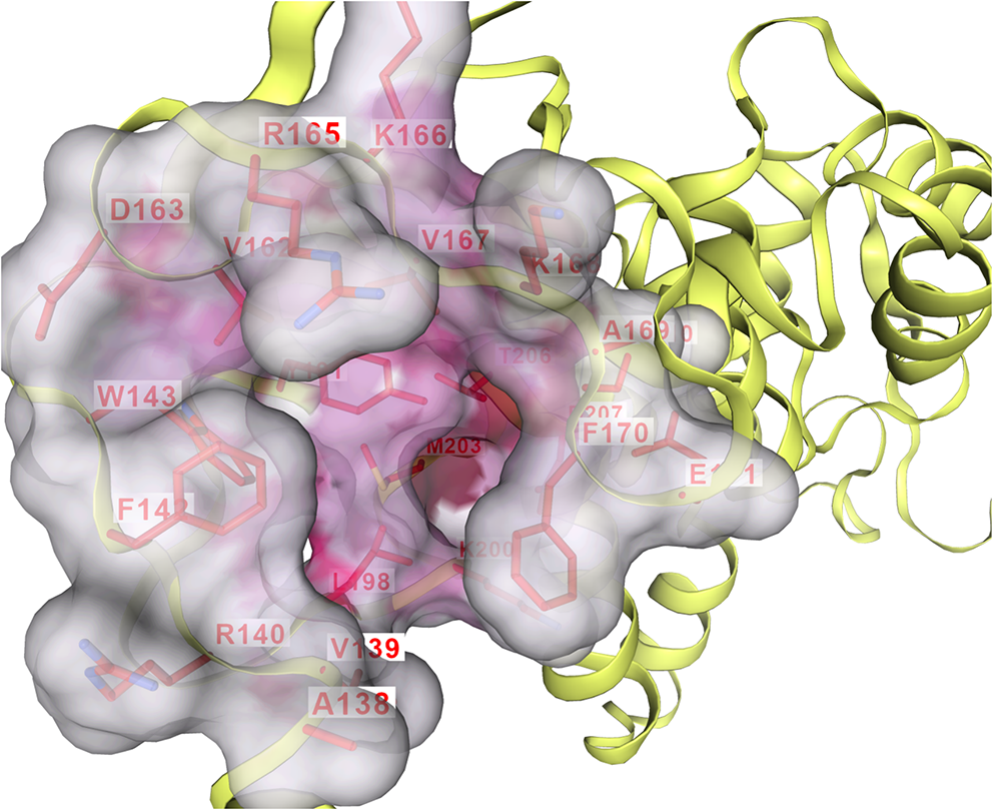
Predicted binding pocket in the modeled *T. cruzi* ATP synthase β-subunit identified by PocketFinder. The top-ranked cavity, selected for docking, is located near the conserved catalytic core and overlaps with regions associated with nucleotide binding.

Both compounds **5** and **6** were successfully docked into the predicted binding pocket of the modeled protein. As results, compound **5** showed a binding affinity of −7.6 kcal/mol, while compound **6** exhibited a binding affinity of −6.8 kcal/mol. Docking scores suggested a moderate to strong interaction potential with the mitochondrial ATP synthase of *T. cruzi* (Figure 5).

**Figure 5:**
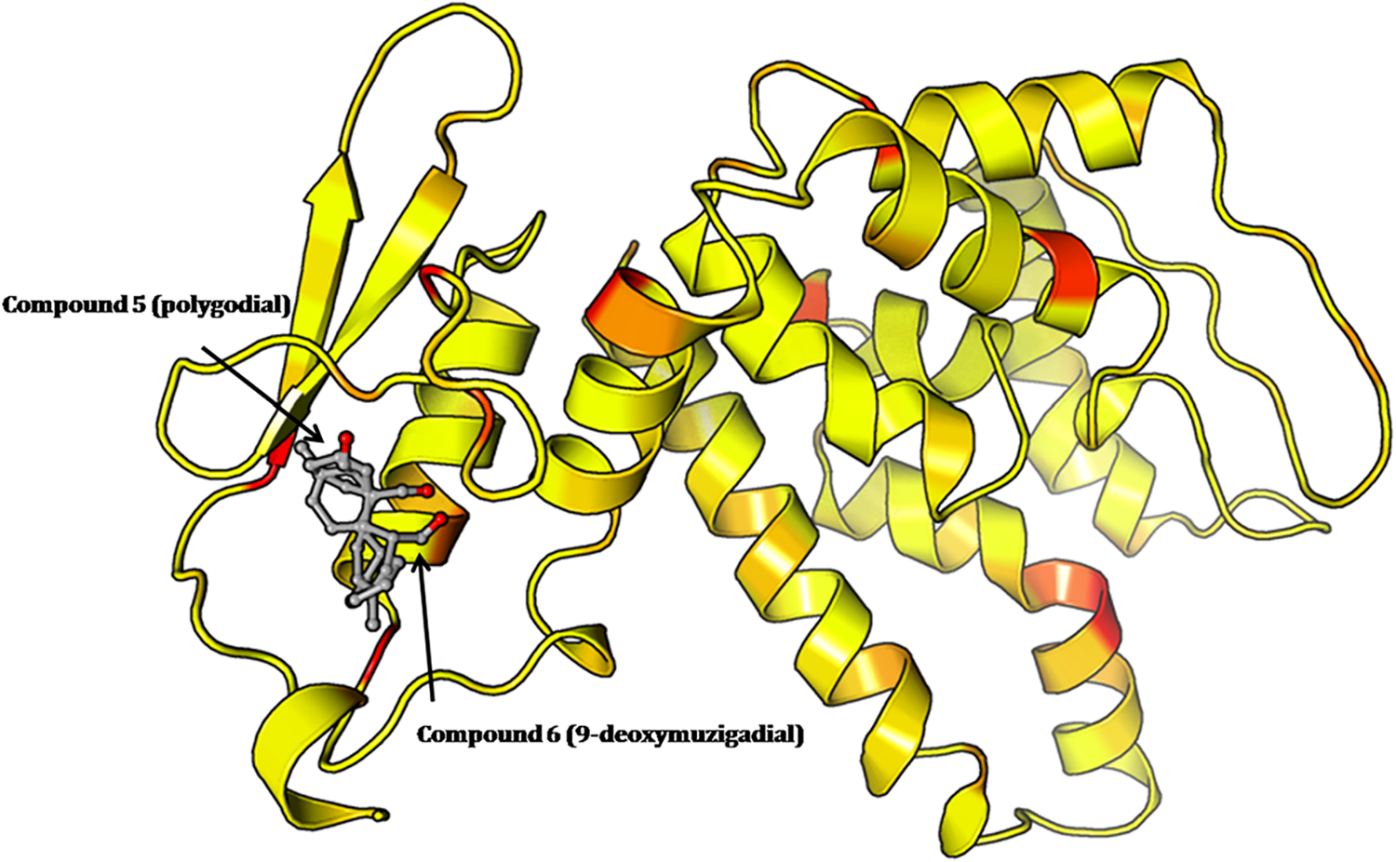
Docking poses of compounds **5** and **6** in the predicted binding pocket of *T. cruzi* ATP synthase β-subunit. Both compounds fit well within the cavity, suggesting stable interactions with the target site.

Interaction profiling of the top-scoring binding poses revealed a consistent set of contacts for both compounds within the predicted binding cavity of the modeled ATP synthase β-subunit (comprising 392 amino acids). On analysis of the molecular interactions, compound **5** interacted with a broader set of residues, including: ALA138, VAL139, ARG140, PHE142, TRP143, VAL162, ARG165, LYS166, VAL167, LYS168, ALA169, PHE170, THR173, TYR191, LEU198, LYS200, MET203, THR206, and ASP207 (Figure 6a). These residues were predominantly located within the core of the binding pocket, engaging the ligand through a combination of hydrogen bonding, hydrophobic contacts, and polar interactions. The aldehyde moiety of compound **5** was oriented toward polar residues such as ARG140 and ASP207, which may contribute to its stronger binding affinity and stabilization within the pocket. In comparison, compound **6** exhibited interactions with a partially overlapping but narrower set of residues, specifically: ALA138, VAL139, ARG140, PHE142, TRP143, VAL162, ARG165, LYS166, VAL167, LYS168, ALA169, PHE170, THR173, TYR191, and LEU198 (Figure 6b).

**Figure 6:**
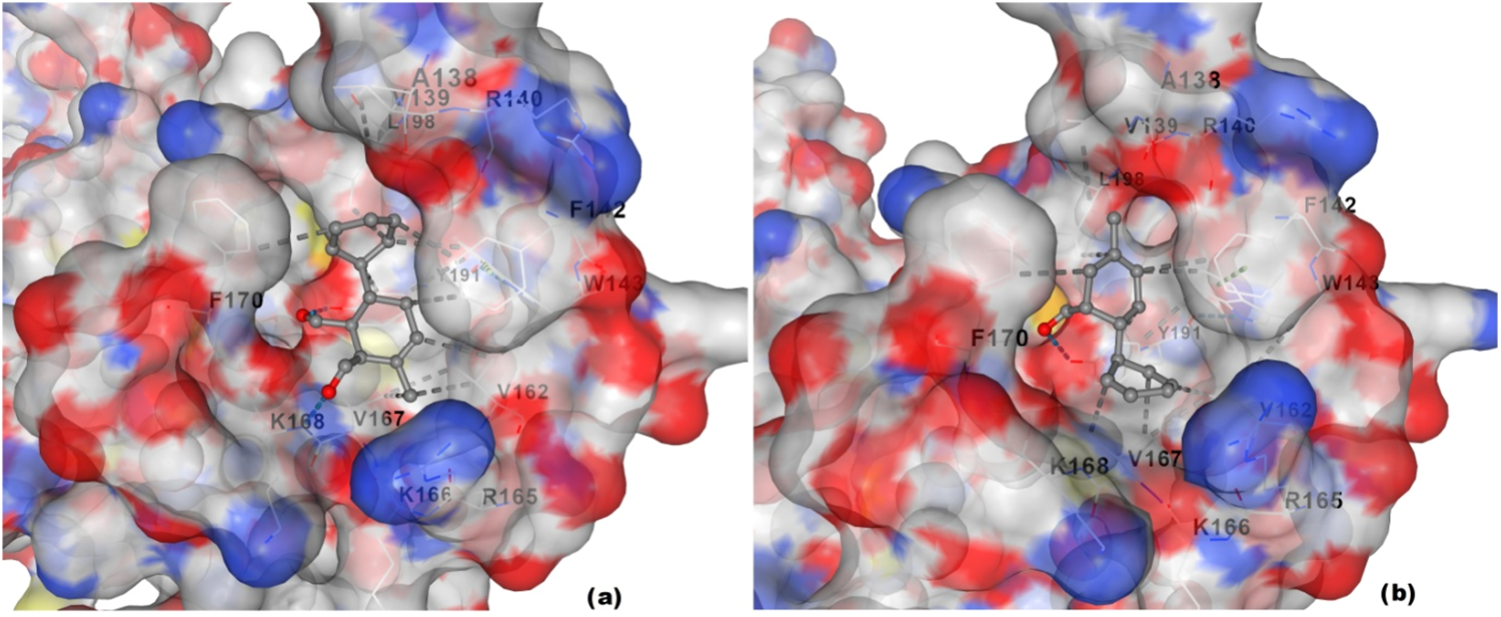
Molecular interactions of docked compounds within the binding pocket of *T. cruzi* ATP synthase β-subunit. Compounds **5** (a) and **6** (b) showing spatial orientation and residue-level contacts within the predicted binding cavity.

While this profile reflected similar positioning within the same cavity, the absence of interactions with key polar or charged residues like ASP207 might underline the slightly reduced binding strength observed relative to compound **5**. These residue-specific contacts suggested a favorable and stable orientation of both ligands within the predicted pocket and offered a plausible molecular explanation for their previously reported *in vitro* efficacy.

Additionally, polygodial (**5**) has been shown to affect the mitochondria of *Leishmania infantum*, a protozoan parasite that causes visceral leishmaniasis. In a previous study by our group, compound **5** caused ultrastructural damage to *Leishmania*, leading to intense mitochondrial swelling after a short incubation period.^7^ These data may further support the potential targeting of ATP synthase by polygodial, as suggested by our *in silico* studies.

Overall, these findings complemented the *in vitro* data for compounds **5** and **6**, which showed the highest activity against *T. cruzi* trypomastigote and amastigote forms with high selectivity indices. The docking and interaction analysis provided a potential molecular basis for their bioactivity, supporting the hypothesis that the mitochondrial ATP synthase is a potential drug target. To reinforce this conclusion, we then did comparative molecular docking with four other potential drug targets (Table 3).

**Table 3:** Comparative Molecular Docking Scores from Four Potential Drug Targets to Bioactive Compounds **5** and **6**, Isolated from *D. brasiliensis*

		compounds	
target protein	PDB ID/model	**5** (kcal/mol)	**6** (kcal/mol)
trypanothione reductase	1AOG	–5.3	–5.8
cruzipain	1ME3	–4.6	–4.1
mitochondrial ATP synthase	modeled	–7.6	–6.8
CYP51	3GW9	–5.8	–5.2

Comparative docking analysis across four *T. cruzi* targets revealed that the mitochondrial ATP synthase exhibited the strongest binding affinities for both compounds **5** (−7.6 kcal/mol) and **6** (−6.8 kcal/mol), compared to trypanothione reductase, Cruzipain, and CYP51. While the latter three targets are well-characterized, the consistently higher interaction potential observed with ATP synthase suggested a more favorable and stable binding environment. Given the enzyme’s essential role in parasite energy metabolism and its structural divergence from the human homologue, mitochondrial ATP synthase represents a hypothetically selective and mechanistically relevant target. These findings support its prioritization for future functional validation and structure-based optimization.

## 3 Conclusions

In conclusion, this study investigated the anti-*T. cruzi* potential of the extract from branches of *D. brasiliensis*, which yielded six sesquiterpenes including four bisabolanes (**1**–**4**) and two drimanes (**5** and **6**) through successive chromatographic separations. Isolated compounds demonstrated activity against trypomastigote and amastigote forms of *T. cruzi*, with compounds **1**, **4**–**6** exhibiting similar efficacies to the reference drug benznidazole and reduced cytotoxicity against NCTC cells (CC_50_ > 200 μM). An in silico molecular docking exercise suggested that the more active compounds **5** and **6** could interact favorably with the *T. cruzi* mitochondrial ATP synthase protein. These results highlight the antitrypanosomal potential of *D. brasiliensis* metabolites and contribute valuable insights toward the development of effective and selective therapeutic agents for Chagas disease.

## 4 Experimental Section

### 4.1 General

^1^H and ^13^C NMR spectra were recorded on a Varian INOVA spectrometer operating at 500 and 125 MHz for ^1^H and ^13^C nucleus, respectively, using CDCl_3_ (Aldrich) as the solvent and TMS (Aldrich) as the internal standard. ESI-HRMS spectrum was measured on a Bruker Daltonics MicroTOF QII spectrometer. IR spectra were recorded on a PerkinElmer Infrared Spectrometer model 1750. Specific optical rotations were obtained on a JASCO polarimeter model P-2000 with Na filter (λ = 588 nm). Silica gel (230–400 mesh, Merck) was used for column chromatography (CC), and silica gel 60 PF_254_ (Merck) was used for analytical TLC separations.

### 4.2 Plant Material

Branches of *D. brasiliensis* were collected in the Serra do Cipó National Park, Minas Gerais State, Brazil, on March 2021 by Dr. Guilherme M. Antar and received a registration code at SISGEN A4123E4. A voucher specimen (SPF4105) has been deposited at the Botanical Institute of São Paulo, Sao Paulo State, Brazil.

### 4.3 Extraction and Isolation

The branches (316 g) were dried at 30 °C for 2 days and then grounded. The obtained material was extracted using hexane (10 × 500 mL) at room temperature. After concentration under reduced pressure, 17 g of crude extract were obtained. Part of this extract (15 g) was chromatographed over silica gel column eluted with mixtures of hexane:EtOAc (9:1, 8:2, 7:3, 1:1, 6:4, 3:7, 2:8, and 1:9) and pure EtOAc to afford nine groups (A–I), being the bioactivity concentrated on groups A, C, D, F, and G. Groups A (344 mg) and C (262 mg) were individually chromatographed over a silica gel column, eluted with hexane/EtOAc (9:1 and 8:2) to afford **4** (118.1 mg) and **3** (167.8 mg), respectively. Group D (451 mg) was chromatographed over silica gel/AgNO_3_ (10%) eluted with hexane/EtOAc 7:3 to give **5** (223.0 mg) and **6** (35.3 mg). Groups F (122 mg) and G (319 mg) were pooled together and subjected to Sephadex LH-20 column chromatography eluted with MeOH to give five groups (FG-1 to FG-5). Bioactive groups FG-3 (79.9 mg) and FG-4 (36.5 mg) were individually chromatographed over a silica gel column, eluted with hexane:EtOAc 7:3 to afford **1** (2.0 mg) and **2** (7.1 mg), respectively.

#### 4.3.1 rel-(3*S*,7*R*)-3-Hydroxy-bisabola-1­(6),10-dien-2-one (**1**)

White amorphous solid; [α]_D_^25^ +16.0 (*c* 0.02, MeOH). FT-IR (MeOH) ν_max_/cm^–1^: 3650, 2890, 1660, 1450, 1145. ESI-HRMS *m*/*z* 237.1843 [M + H]^+^. ^1^H NMR (500 MHz, CDCl_3_), δ 5.89 (s, H-1), 5.05 (br s, H-10), 2.40 (m, H-8_eq_), 2.30 (m, H-7), 2.15 (m, H-4_eq_), 1.98 (m, H-4_ax_), 1.92 (m, H-8_ax_), 1.90 (m, H-9), 1.68 (s, H-12), 1.57 (s, H-13), 1.53 (m, H-5_eq_), 1.43 (m, H-5_ax_), 1.30 (s, H-14), 1.10 (d, *J* = 6.9 Hz, H-15). ^13^C NMR (125 MHz, CDCl_3_), δ 202.8 (C-2), 171.7 (C-6), 132.4 (C-11), 123.7 (C-10), 121.8 (C-1), 72.8 (C-3), 41.2 (C-7), 35.8 (C-4), 35.2 (C-5), 26.0 (C-8) 25.9 (C-9), 25.8 (C-12), 24.3 (C-14), 19.3 (C-15), 17.8 (C-12).

#### 4.3.2 rel-(2S,3S,7R)-Bisabola-1­(6),10-diene-2,3-diol (**2**)

White amorphous solid; [α]_D_^25^ −21.0 (*c* 0.02, MeOH). FT-IR (MeOH) ν_max_/cm^–1^: 3480, 2891, 1780, 1450, 1200. ESI-HRMS *m*/*z* 261.1812 [M + Na]^+^. ^1^H NMR (500 MHz, CDCl_3_), δ 5.35 (br s, H-1), 5.09 (t, *J* = 7.5 Hz, H-10), 4.03 (br s, H-2), 2.15 (m, H-8_eq_), 2.10 (m, H-7), 2.05 (m, H-8_ax_), 1.90 (m, H-9), 1.73 (m, H-4_eq_), 1.69 (m, H-4_ax_), 1.67 (s, H-12), 1.58 (s, H-13), 1.40 (m, H-5_eq_), 1.31 (m, H-5_ax_), 1.20 (s, H-14), 1.00, (d, *J* = 6.5 Hz, H-15). ^13^C NMR (125 MHz, CDCl_3_), δ 145.0 (C-6), 131.4 (C-11), 124.4 (C-10), 122.3 (C-1), 74.7 (C-2), 72.2 (C-3), 39.8 (C-7), 35.2 (C-5), 33.4 (C-4), 26.1 (C-9), 25.7 (C-12), 24.0 (C-8), 21.1 (C-14), 19.7 (C-15), 17.6 (C-13).

### 4.5 Mammalian Cells and Parasite Maintenance

The trypomastigote forms of *T. cruzi* (Y strain) were maintained in culture using Rhesus monkey kidney cells (LLC-MK2, ATCC CCL-7). These cells were cultured in RPMI-1640 medium supplemented with 2% fetal bovine serum (FBS) and incubated at 37 °C in a humidified atmosphere containing 5% CO_2_. Mouse fibroblast cells (NCTC clone L929, ATCC) and LLC-MK2 cells were similarly maintained in RPMI-1640 medium supplemented with 10% FBS under identical incubation conditions (37 °C, 5% CO_2_). Peritoneal macrophages were isolated from BALB/c mice by peritoneal lavage using RPMI-1640 medium supplemented with 10% FBS, followed by incubation at 37 °C in a 5% CO_2_ humidified atmosphere. In addition, the human monocytic cell line THP-1 (ATCC TIB-202) was cultured in RPMI-1640 medium supplemented with 20% FBS and 2 μM glutamine, and maintained at 37 °C in a humidified 5% CO_2_ environment.^25^

### 4.6 Determination of the EC_50_ Against Trypomastigotes and Intracellular Amastigotes Forms

Compounds **1**–**6** were initially dissolved in DMSO and subsequently diluted in RPMI-1640 medium to the desired concentrations before being dispensed into 96-well microplates. *T. cruzi* trypomastigotes were then added at a density of 1 × 10^6^ parasites per well. The plates were incubated for 24 h at 37 °C in a humidified atmosphere containing 5% CO_2_. Following incubation, the 50% effective concentration (EC_50_) was determined using the resazurin reduction assay. Absorbance readings were acquired at 570 nm using a microplate spectrophotometer (FilterMax F5). Untreated parasites served as the negative control, while benznidazole, a standard reference drug, was used as the positive control.

The antiparasitic activity of compounds **1**–**6** against the intracellular amastigote forms of *T. cruzi* was assessed using an *in vitro* infection model with peritoneal macrophages. Macrophages were seeded in 16-well plates at a density of 1 × 10^5^ cells per well and incubated at 37 °C in a humidified atmosphere containing 5% CO_2_ for 24 h to promote adherence. Cells were then infected with trypomastigotes for 2 h under the same conditions to allow parasite internalization. Following infection, noninternalized parasites were removed by washing with RPMI-1640 medium. The infected macrophages were subsequently treated with serial dilutions of compounds **1**–**6** (150.0–1.6 μM) and incubated for an additional 48 h at 37 °C in 5% CO_2_ to evaluate their antitrypanosomal efficacy.^22,26^

### 4.7 Cytotoxic Assay Against Mammalian Cells

The cytotoxicity of compounds **1**–**6** was assessed using NCTC (clone L929) cell line. NCTC cells (6 × 10^4^ cells/well) were seeded into 96-well plates and treated with serial dilutions of the test compounds prepared in RPMI-1640 medium supplemented with 10% fetal bovine serum (FBS). Following a 48-h incubation at 37 °C in a humidified atmosphere with 5% CO_2_, cell viability was determined using the MTT colorimetric assay. The 50% cytotoxic concentration (CC_50_) was calculated based on absorbance measurements at 570 nm using a FilterMax F5 microplate reader (Molecular Devices).^27^

### 4.8 Statistical Analysis

The CC_50_ and EC_50_ values were obtained from sigmoid dose–response curves using Graph-Pad Prims 6.0 software. All samples were tested in triplicate.

### 4.9 *In Silico* Studies

*In silico* analyses were conducted using the SwissADME platform (http://www.swissadme.ch/) to assess physicochemical properties, pharmacokinetic behavior, drug-likeness, and medicinal chemistry parameters.^21^ This computational tool evaluates a wide range of descriptors, including: (i) physicochemical characteristics such as the number of rotatable bonds, hydrogen bond donors and acceptors; (ii) ADMET-related properties (Absorption, Distribution, Metabolism, Excretion, and Toxicity); (iii) lipophilicity, expressed as log *P*; (iv) hydrophilicity, expressed as log *S*; (v) pharmacokinetic parameters including predicted gastrointestinal absorption and cytochrome P450 inhibition profiles; (vi) drug-likeness based on major pharmaceutical industry filters (Lipinski, Veber, Egan, and Muegge); and (vii) structural alerts for Pan-Assay Interference Compounds (PAINS).

### 4.10 Protein Modeling

The amino acid sequence of the mitochondrial ATP synthase β-subunit from *T. cruzi* was retrieved from the UniProt database (accession ID: K2MZU1), comprising 392 amino acids. Since no experimentally determined structure was available in the Protein Data Bank (PDB), a three-dimensional structural model was generated using the AlphaFold Protein Structure Database.^28^ The prediction utilized AlphaFold’s default settings and was based on multiple sequence alignments and structural templates, yielding a high-confidence model suitable for further computational analysis.

### 4.11 Validation of Modeled Structure

To evaluate the structural quality of the modeled *T. cruzi* ATP synthase β-subunit, the model was first subjected to validation using the ProSA-web server.^29^ The overall model quality was assessed via the *Z*-score, which reflects the model’s compatibility with native protein structures of similar size. Additionally, structural superimposition was performed between the modeled *T. cruzi* protein and the available cryo-EM structure of *T. brucei* mitochondrial ATP synthase (PDB ID: 8AP6) using PyMOL.^30^ This comparative alignment was used to assess overall fold conservation and spatial arrangement of catalytically relevant regions.

### 4.12 Binding Site Prediction

The binding pocket of the modeled *T. cruzi* ATP synthase was identified using the PocketFinder tool.^31^ This method employs a grid-based approach to detect clefts and surface depressions likely to act as ligand binding sites based on geometric and energetic parameters. The top-ranked pocket based on volume and depth was selected for subsequent docking studies.

### 4.13 Molecular Docking (MD)

MD investigated the binding potential of compounds **5** and **6**. The 3D structures of the ligands were geometry-optimized and prepared for docking using standard parameters. The docking was executed using AutoDock Vina,^32^ where the grid box was centered over the predicted binding pocket. Default exhaustiveness and scoring parameters were used to evaluate binding affinities and pose stability.

### 4.14 Molecular Interaction Analysis

Postdocking, the top-scoring binding poses were analyzed to identify key molecular interactions between the ligands and the target protein. Visualization was performed using PyMOL^30^ and Discovery Studio Visualizer^33^ to characterize hydrogen bonding, hydrophobic interactions, and potential π-alkyl or van der Waals contacts. Special attention was given to the interaction of ligand functional groups with residues within the binding pocket, as these may correlate with the observed *in vitro* efficacy.

## Supplementary Material



## Data Availability

The data that support the findings of this study are available throughout the manuscript and supporting files.

## Notes

NMR, IR and MS data for compounds **1–6** (PDF)

## ∥

Faculty of Applied Sciences & Biotechnology, Shoolini University, Solan, Himachal Pradesh 173229, India
