# Supplementary material for: Anti-Trypanosoma cruzi Effects of Sesquiterpenoids from Branches of Drimys brasiliensis (Winteraceae)
Source: ACS Omega. 2025 Nov 3;10(44):53307–16. doi: 10.1021/acsomega.5c08083 (PMC12613110; doi:10.1021/acsomega.5c08083)
Supplement: Supplementary file 1 [file ao5c08083_si_001.pdf]

Anti-*Trypanosoma cruzi* effects of sesquiterpenoids from branches of *Drimys brasiliensis* (Winteraceae)

Eric Umehara[1], Dayana A. S. Ferreira,[2] Mariana B. Abbiuzi,[2] Myron Christodoulides[3], Ravi Kant⁋[3], Andre G. Tempone*[2], and João Henrique G. Lago*[1]

[1]Center for Natural and Human Sciences, Federal University of ABC, 09210-580, Santo Andre, SP, Brazil

[2]Laboratory of Pathophysiology, Instituto Butantan, 05503-900, 1500, São Paulo, SP, Brazil

[3]Molecular Microbiology, School of Clinical and Experimental Sciences, Faculty of Medicine, University of Southampton, Southampton, SO166YD, United Kingdom.

SUPPLEMENTARY MATERIAL

*Correspondence: Andre G. Tempone (andre.tempone@butantan.gov.br) and João Henrique G. Lago (joao.lago@ufabc.edu.br)

⁋Current address:

Faculty of Applied Sciences & Biotechnology, Shoolini University, 173229, Solan, Himachal Pradesh, India

Table S1. NMR data of compound **1** (δ/ppm, $CDCl_3$, 500 and 125 MHz)

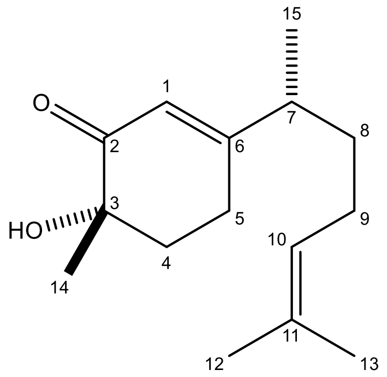

position	δ_H / ppm	δ_C / ppm	HMBC (H → C)	NOESY
**1**	5.89 (s)	121.8	C-3, C-7	H-15, H-7
**2**	-	202.8	-	-
**3**	-	72.8	-	-
**4**	eq 2.15 (m)	35.8	C-2, C-3	H-5_{ax}, H-8, H-14
	ax 1.98 (m)			
**5**	eq 1.53 (m)	35.2	C-6	
	ax 1.43 (m)			
**6**	-	171.7	-	-
**7**	2.30 (m)	41.2	C-1, C-5, C-6, C-9	
**8**	eq 2.40 (m)	26.0	C-6	H-4
	ax 1.92 (m)			
**9**	1.90 (m)	25.9	C-7	
**10**	5.05 (br t, *J* = 7.0 Hz)	123.7	-	H-12
**11**	-	132.4	-	-
**12**	1.68 (s)	25.9	C-10, C-11, C-13	
**13**	1.57 (s)	17.8	C-10, C-11, C-12	
**14**	1.30 (s)	24.3	C-2, C-3, C-4	H-4_{eq}, H-8a
**15**	1.10 (d, *J* = 6.9 Hz)	19.3	C-5, C-6, C-7	H-7, H-8a

Table S2. NMR data of compound **2** (δ/ppm, $CDCl_3$, 500 and 125 MHz)

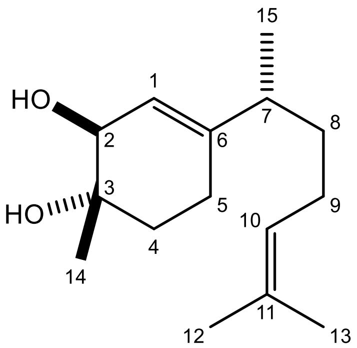

position	δ_H / ppm	δ_C / ppm	HMBC (H → C)	NOESY
**1**	5.35 (br s)	122.3	C-3, C-7	
**2**	4.00 (br s)	74.7	C-1, C-4, C-6	
**3**	-	72.2	-	-
**4**	eq 1.73 (m)	33.4	C-2, C-3, C-6	H-5_{ax}
	ax 1.69 (m)			
**5**	eq 1.40 (m)	35.2	C-3, C-6, C-7	H-4_{eq}, H-15
	ax 1.31 (m)			
**6**	-	145.0	-	-
**7**	2.10 (m)	39.8	C-1, C-5, C-6, C-8	
**8**	eq 2.15 (m)	24.0	C-6, C-10	H-9
	ax 2.05 (m)			
**9**	1.90 (m)	26.1	C-7, C-10, C-11	H-8
**10**	5.09 (t, *J* = 7.5 Hz)	124.4	C-12, C-13	
**11**	-	131.4	-	-
**12**	1.67 (s)	25.7	C-10, C-11, C-13	
**13**	1.58 (s)	17.6	C-10, C-11, C-12	
**14**	1.20 (s)	21.1	C-2, C-3, C-4	H-4_{eq}, H-12, H-15
**15**	1.00 (d, *J* = 6.5 Hz)	19.6	C-6, C-7	H-14

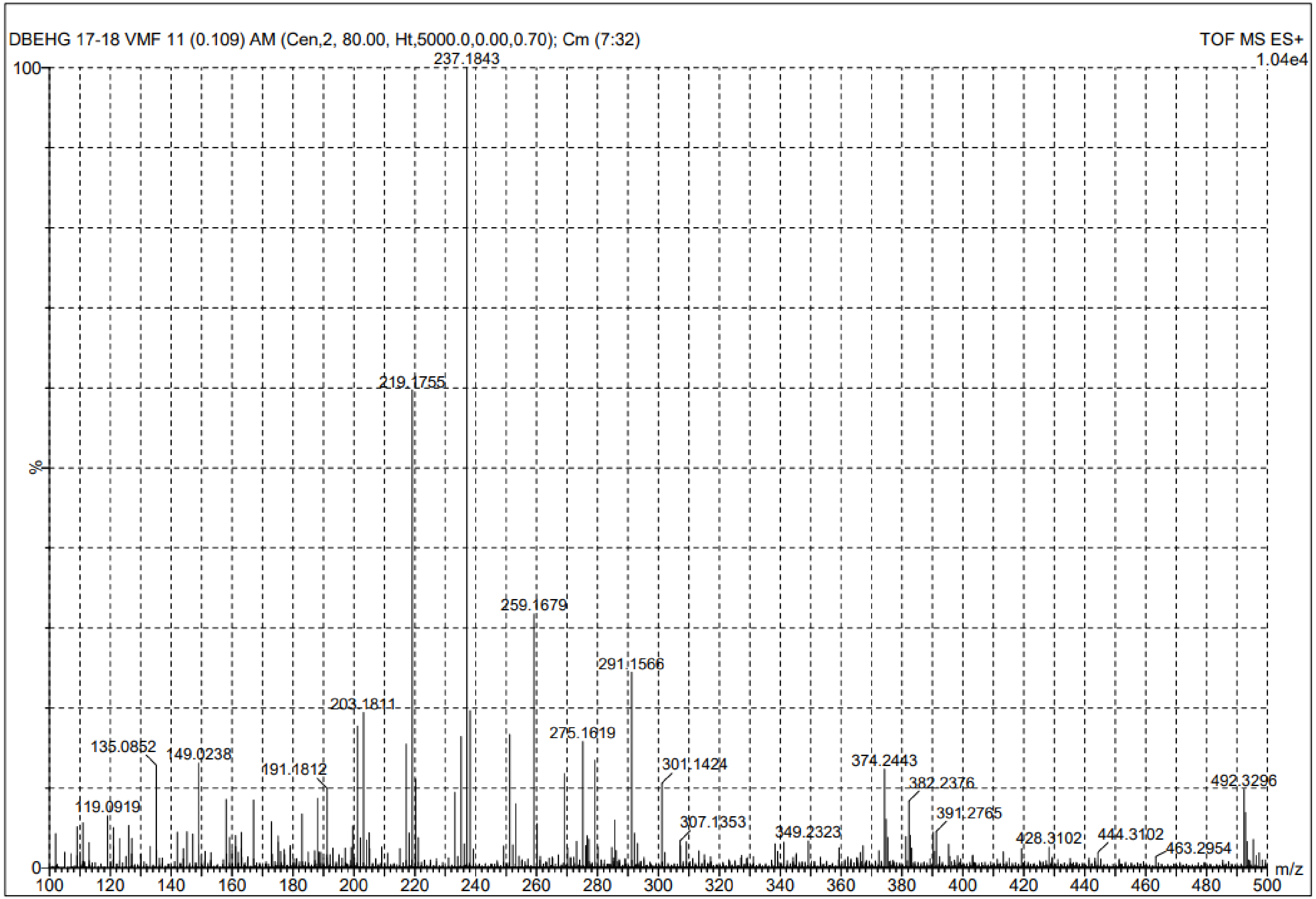

Figure S1. ESI-HRMS spectrum of compound **1**

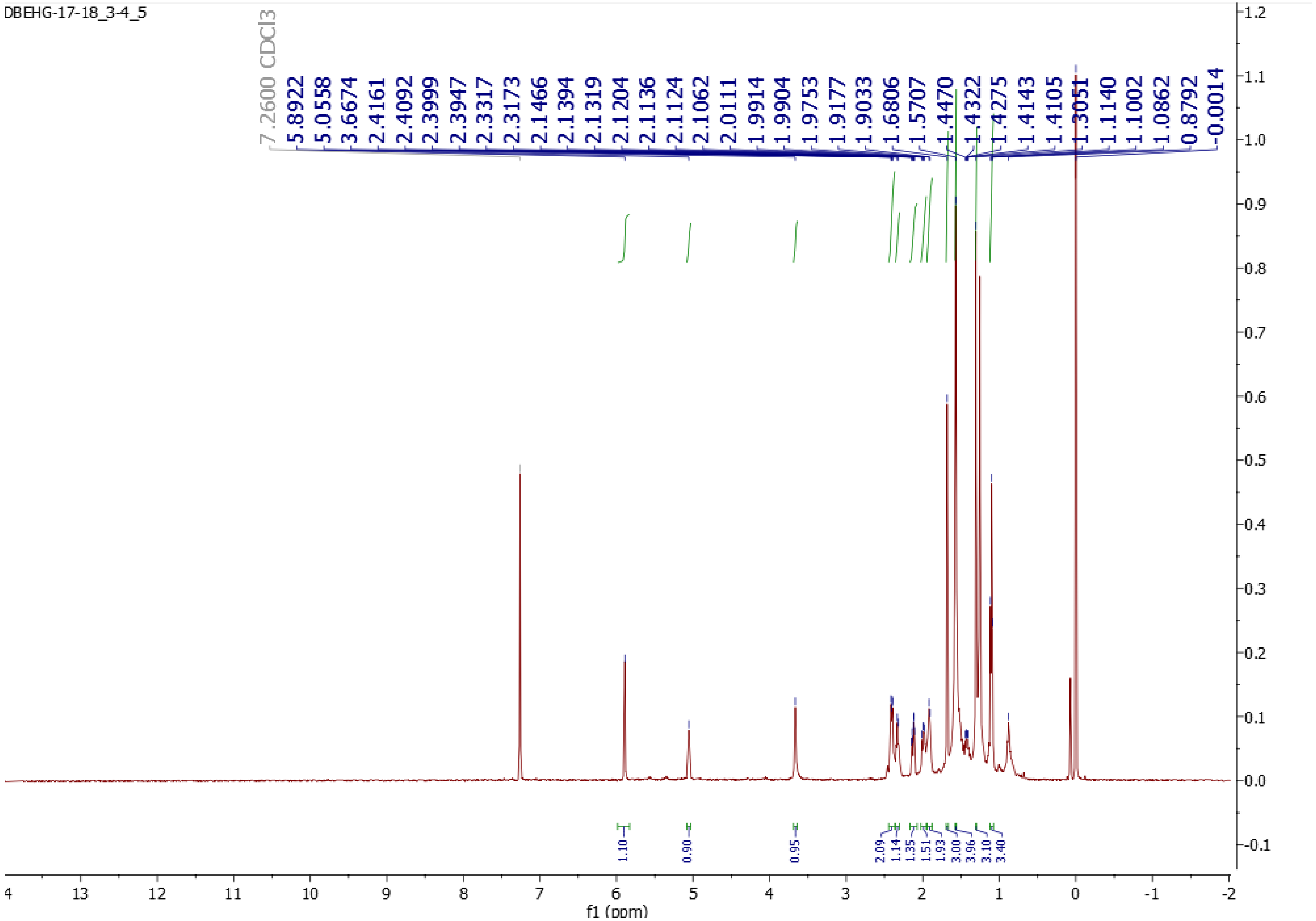

Figure S2. ^{1}H NMR spectrum of compound **1** (δ, $CDCl_3$, 500 MHz)

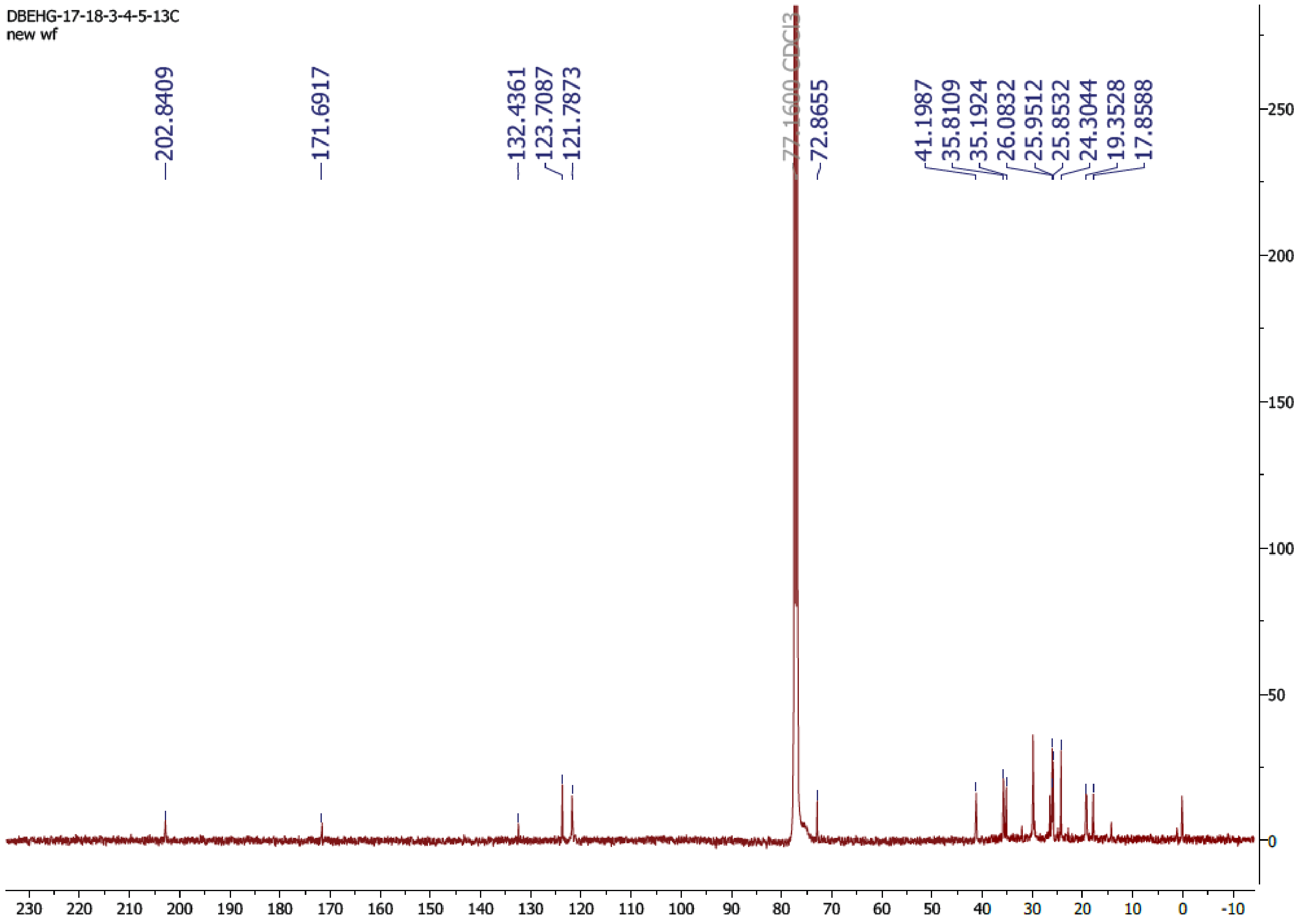

Figure S3. ^{13}C NMR spectrum of compound **1** (δ, $CDCl_3$, 125 MHz)

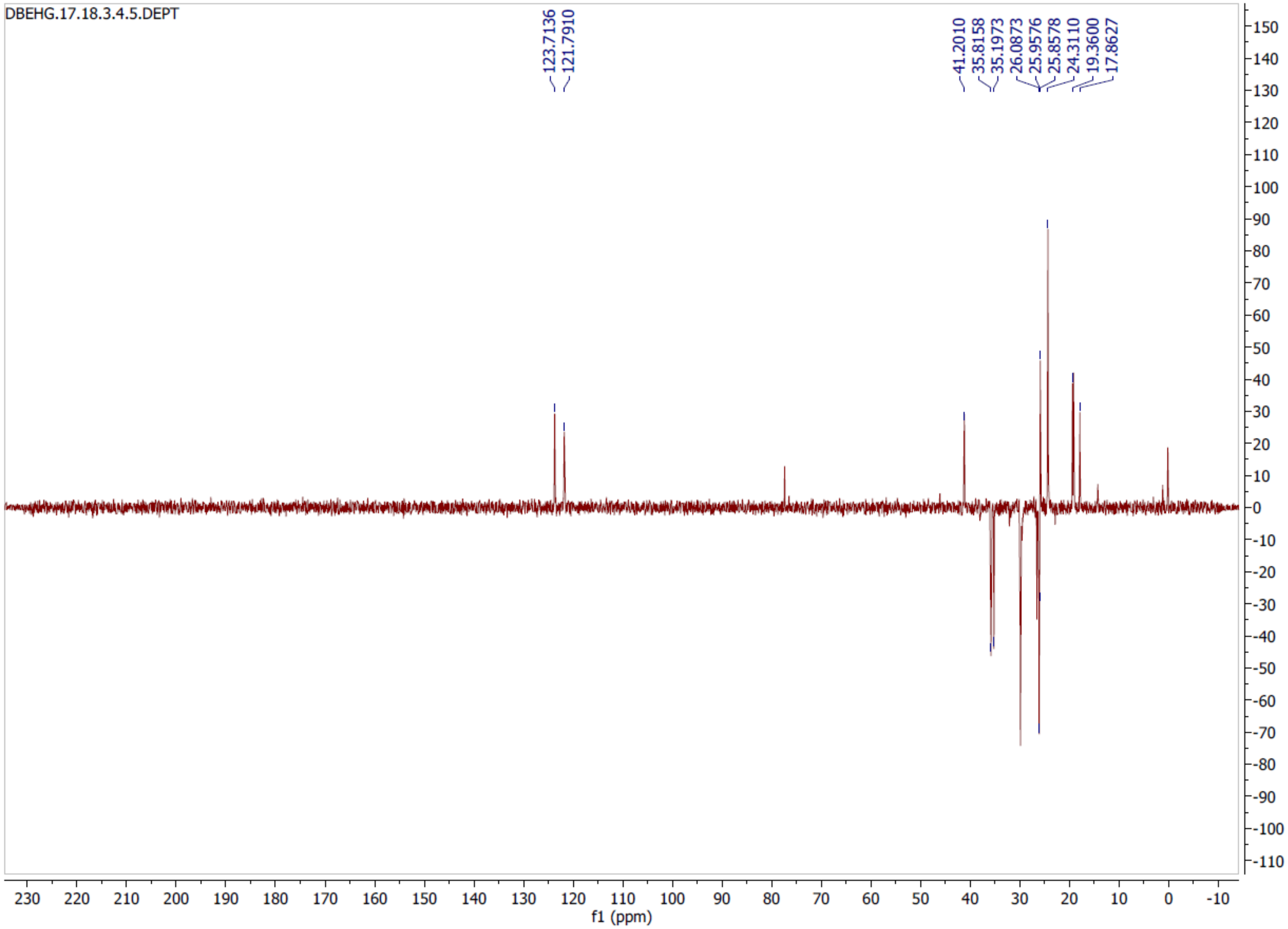

Figure S4. DEPT spectrum of compound **1** (δ, $CDCl_3$, 125 MHz)

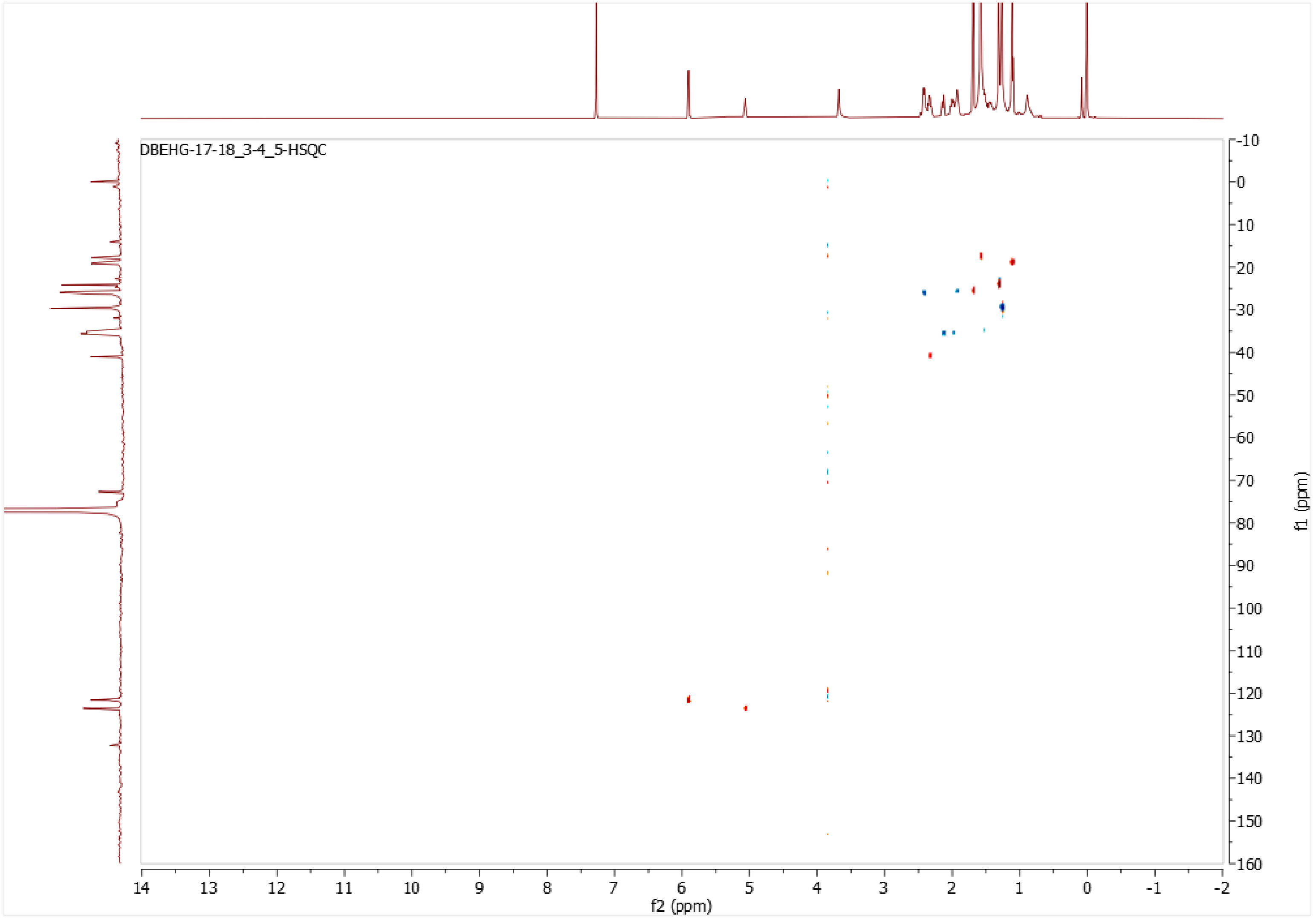

Figure S5. HSQC correlation map of compound **1** (δ, $CDCl_3$, 500 and 125 MHz)

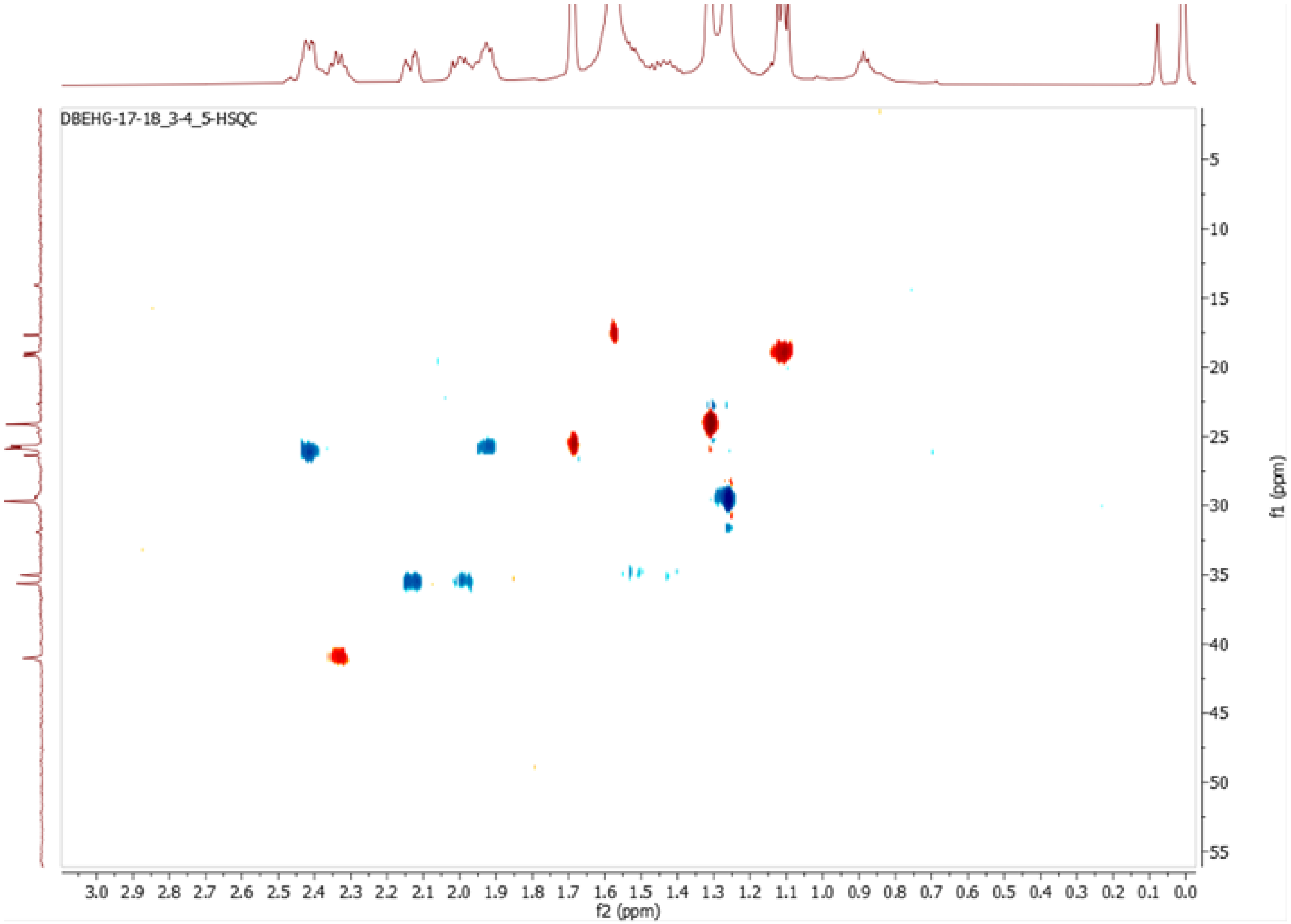

Figure S6. Expansion of HSQC correlation map of compound **1** (δ, $CDCl_3$, 500 and 125 MHz)

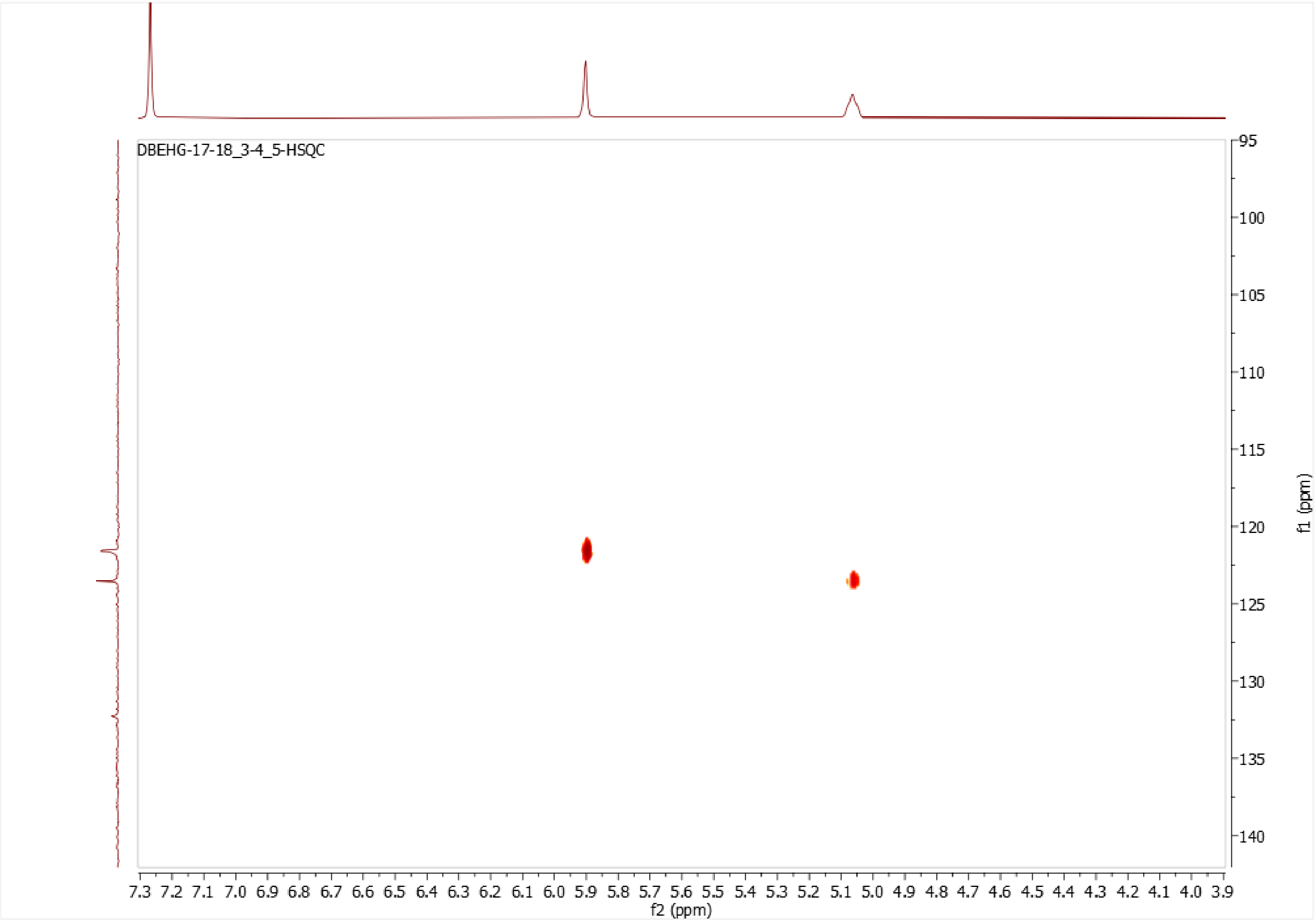

Figure S7. Expansion of HSQC correlation map of compound **1** (δ, $CDCl_3$, 500 and 125 MHz)

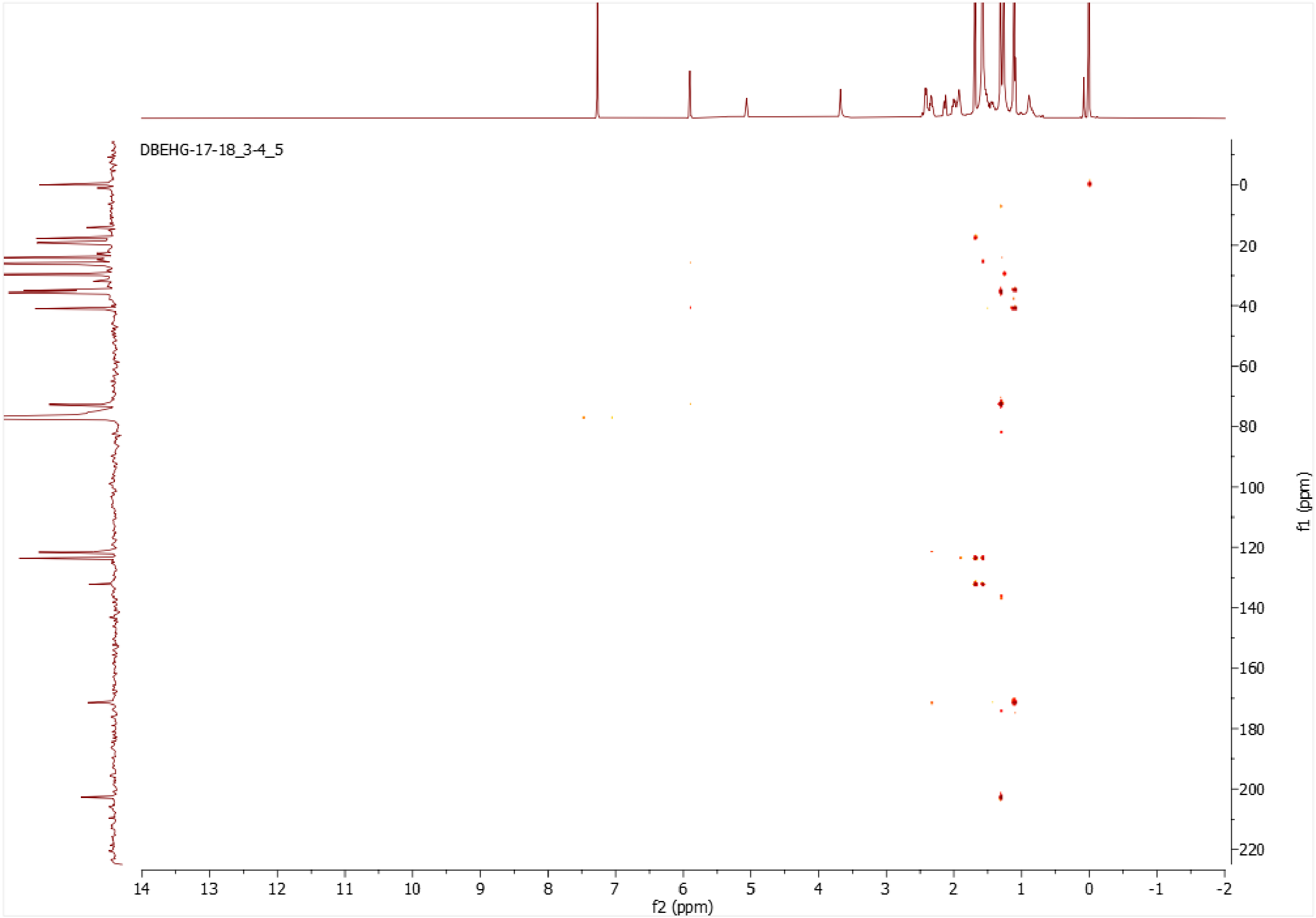

Figure S8. HMBC correlation map of compound **1** (δ, $CDCl_3$, 500 and 125 MHz)

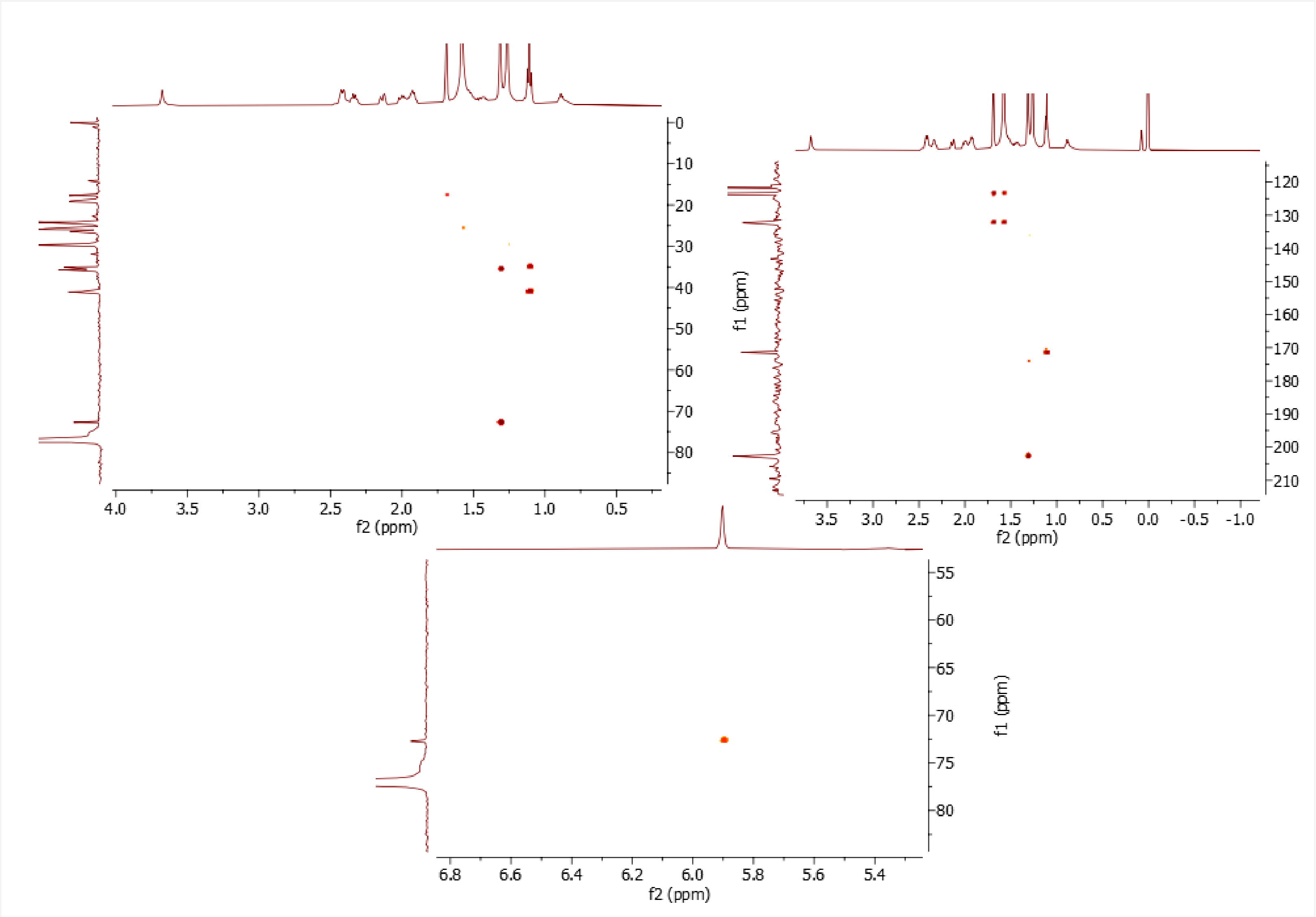

Figure S9. Expansions of HSQC correlation map of compound **1** (δ, $CDCl_3$, 500 and 125 MHz)

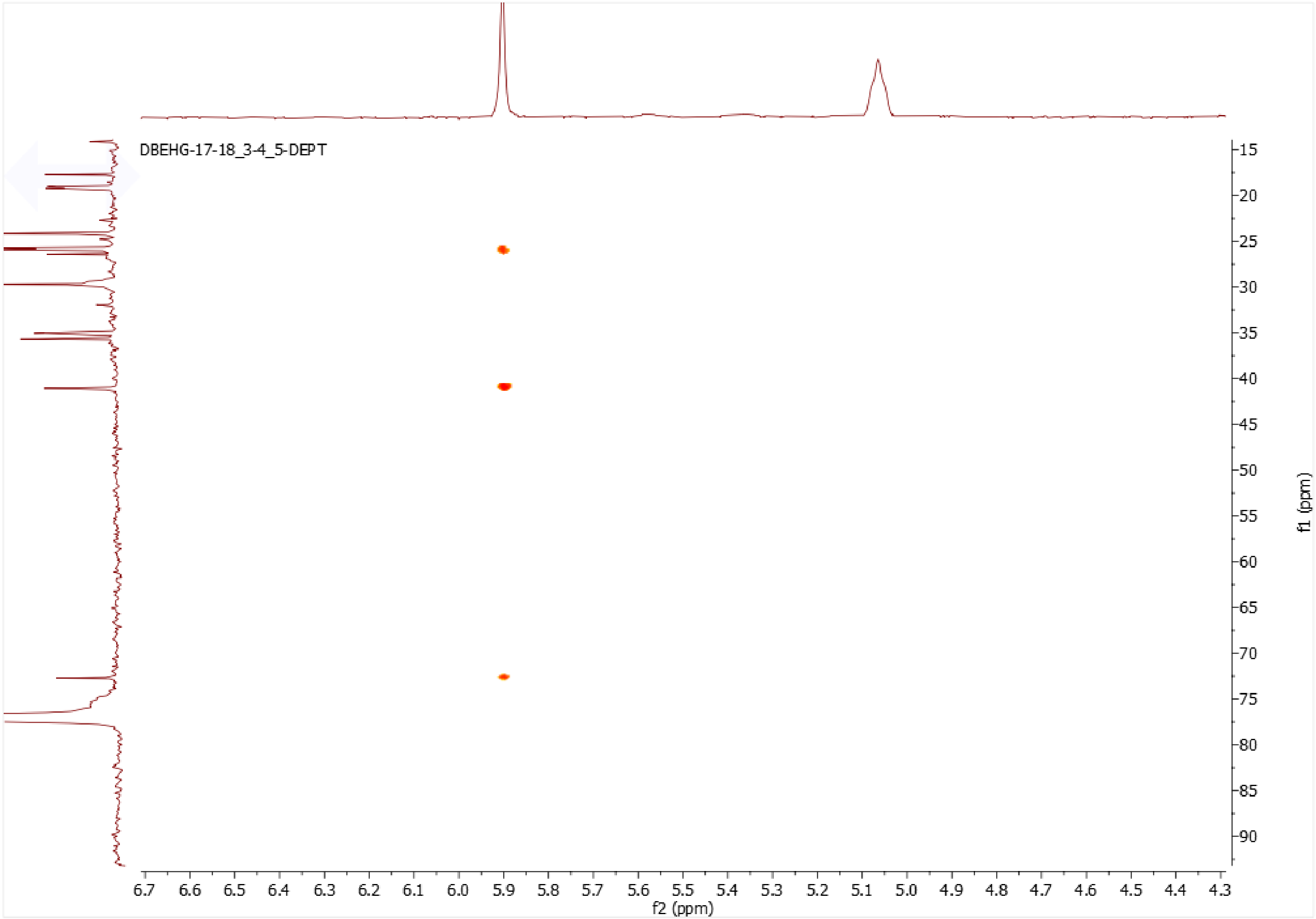

Figure S10. Expansion of HMBC correlation map of compound **1** (δ, $CDCl_3$, 500 and 125 MHz)

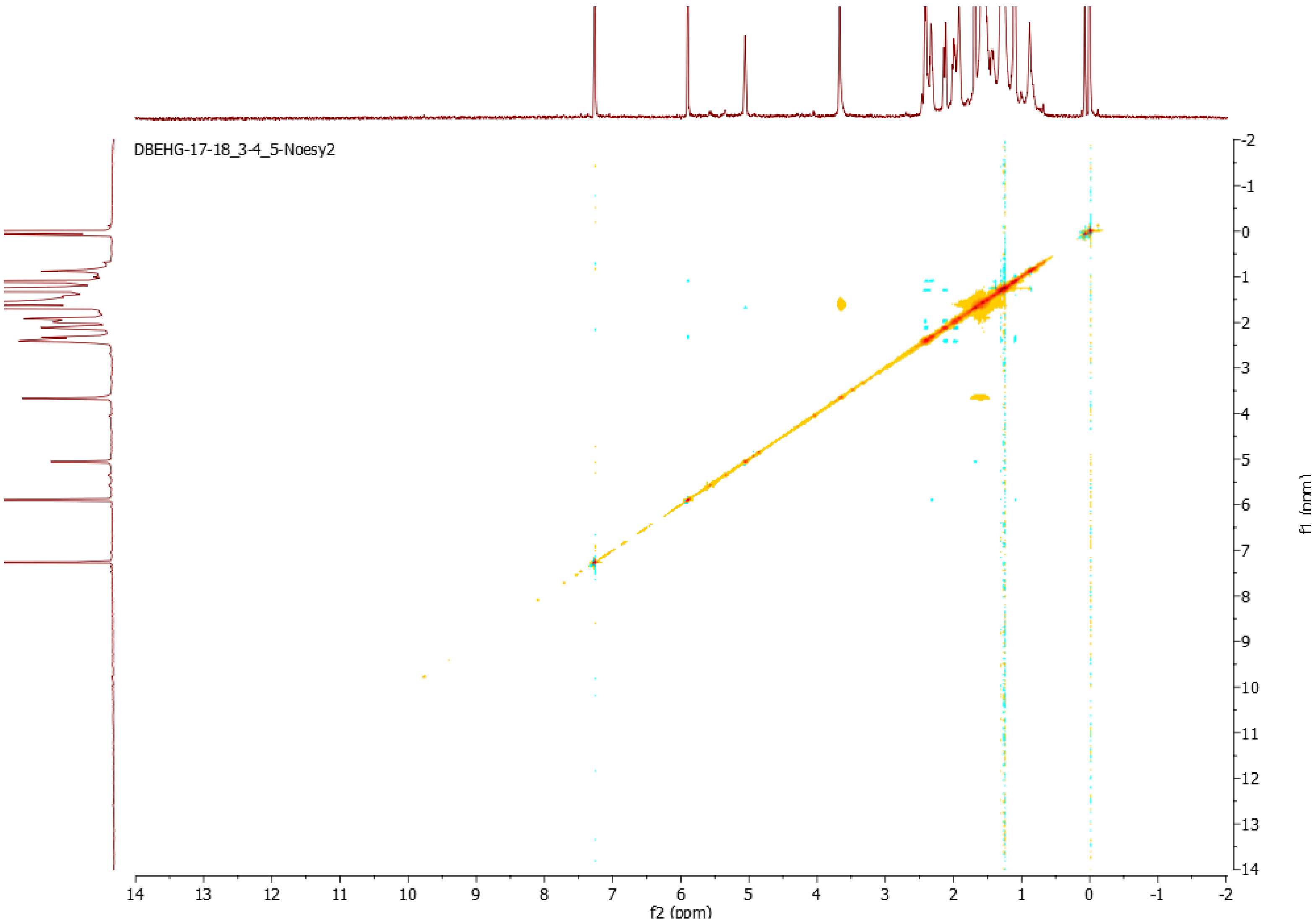

Figure S11. NOESY correlation map of compound **1** (δ, $CDCl_3$, 500 MHz)

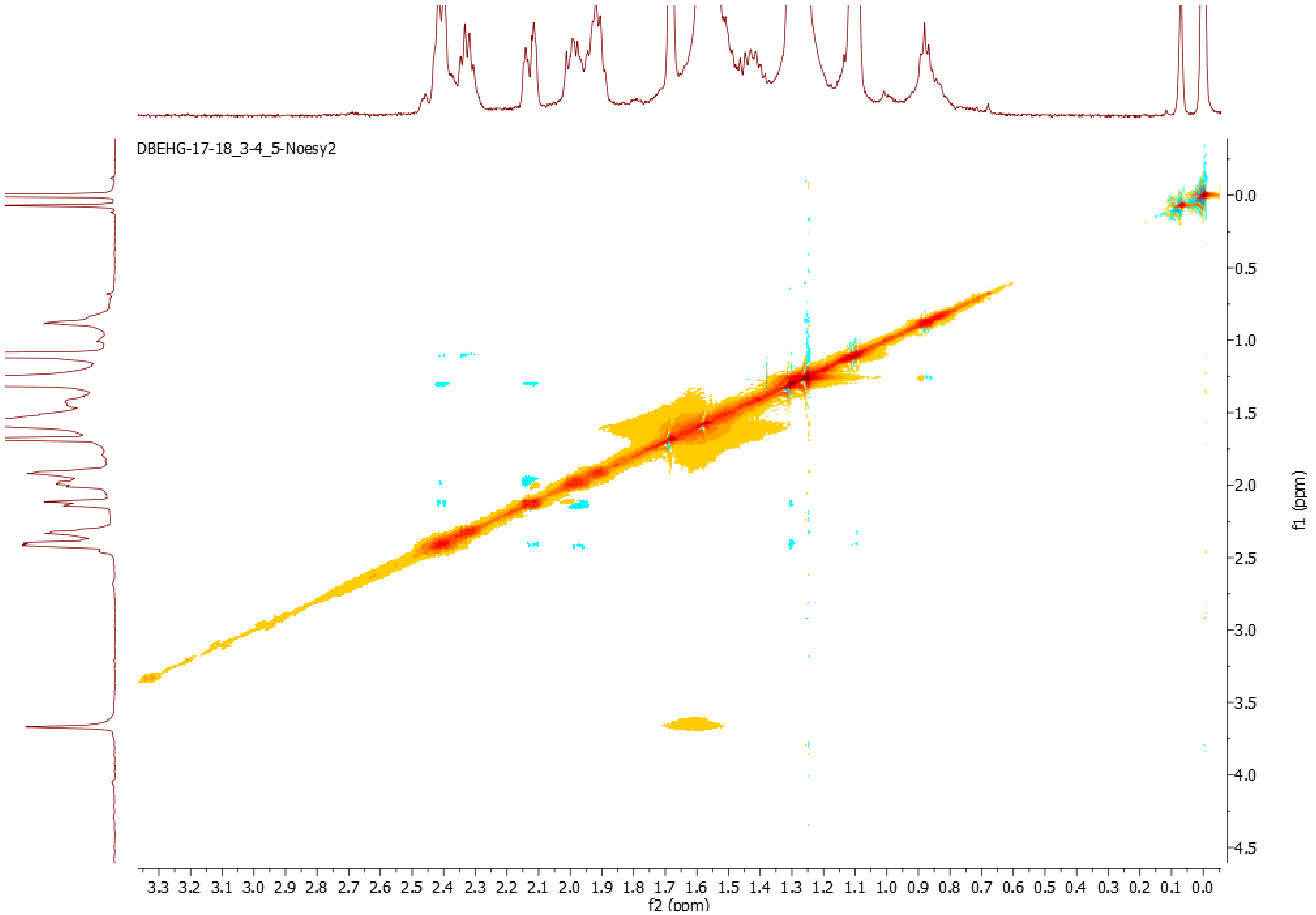

Figure S12. Expansion of NOESY correlation map of compound **1** (δ, $CDCl_3$, 500 MHz)

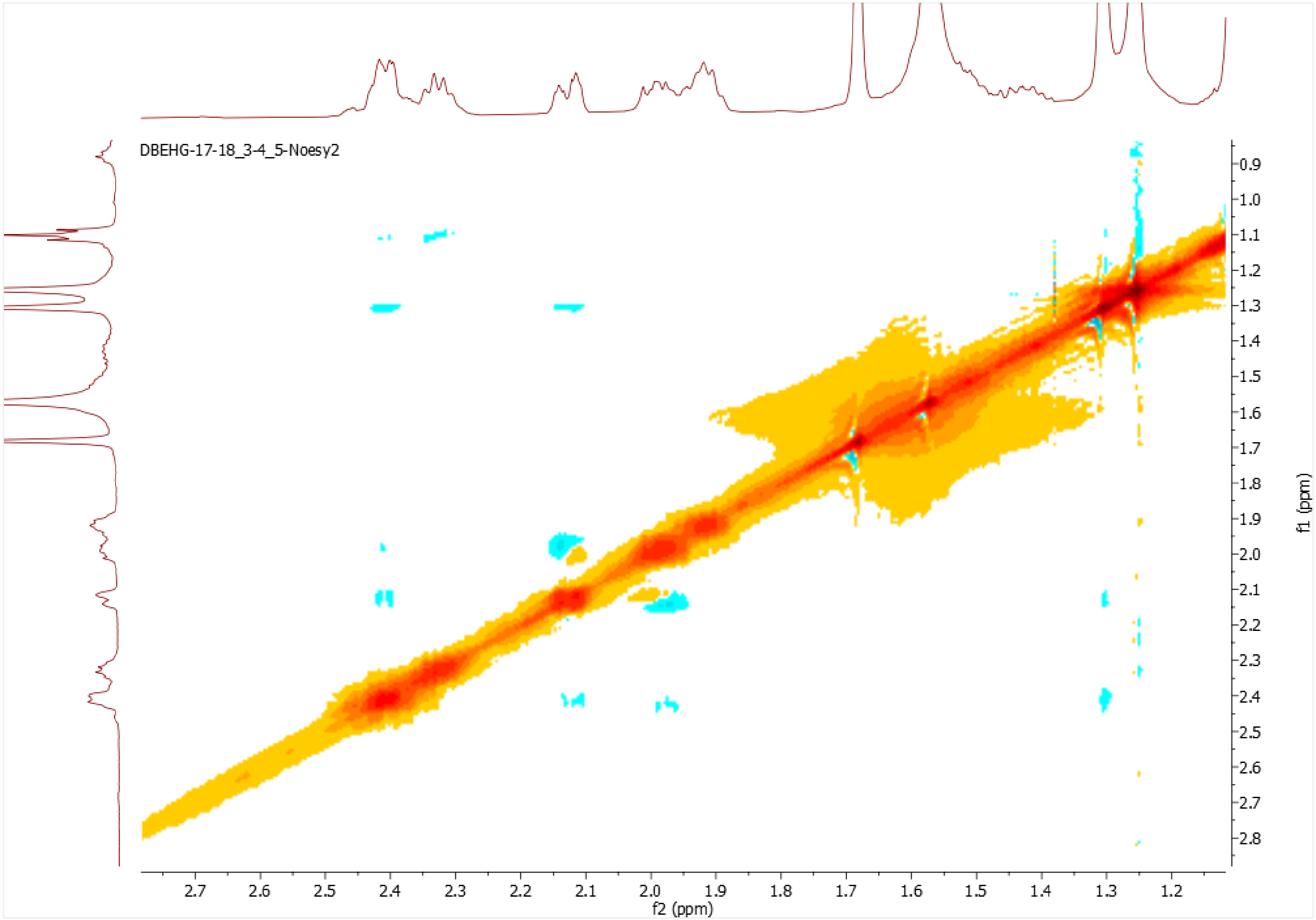

Figure S13. Expansion of NOESY correlation map of compound **1** (δ, $CDCl_3$, 500 MHz)

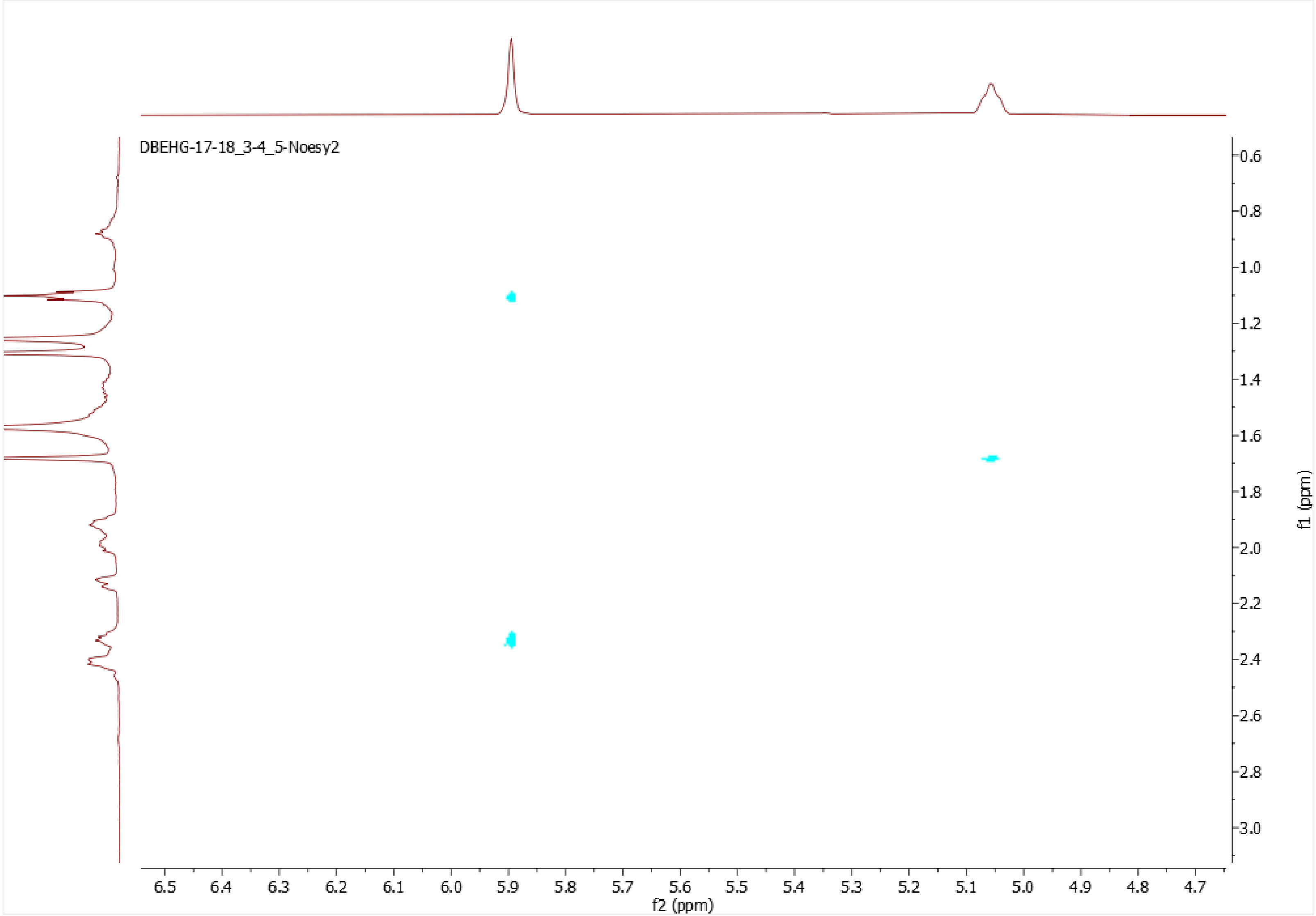

Figure S14. Expansion of NOESY correlation map of compound **1** (δ, $CDCl_3$, 500 MHz)

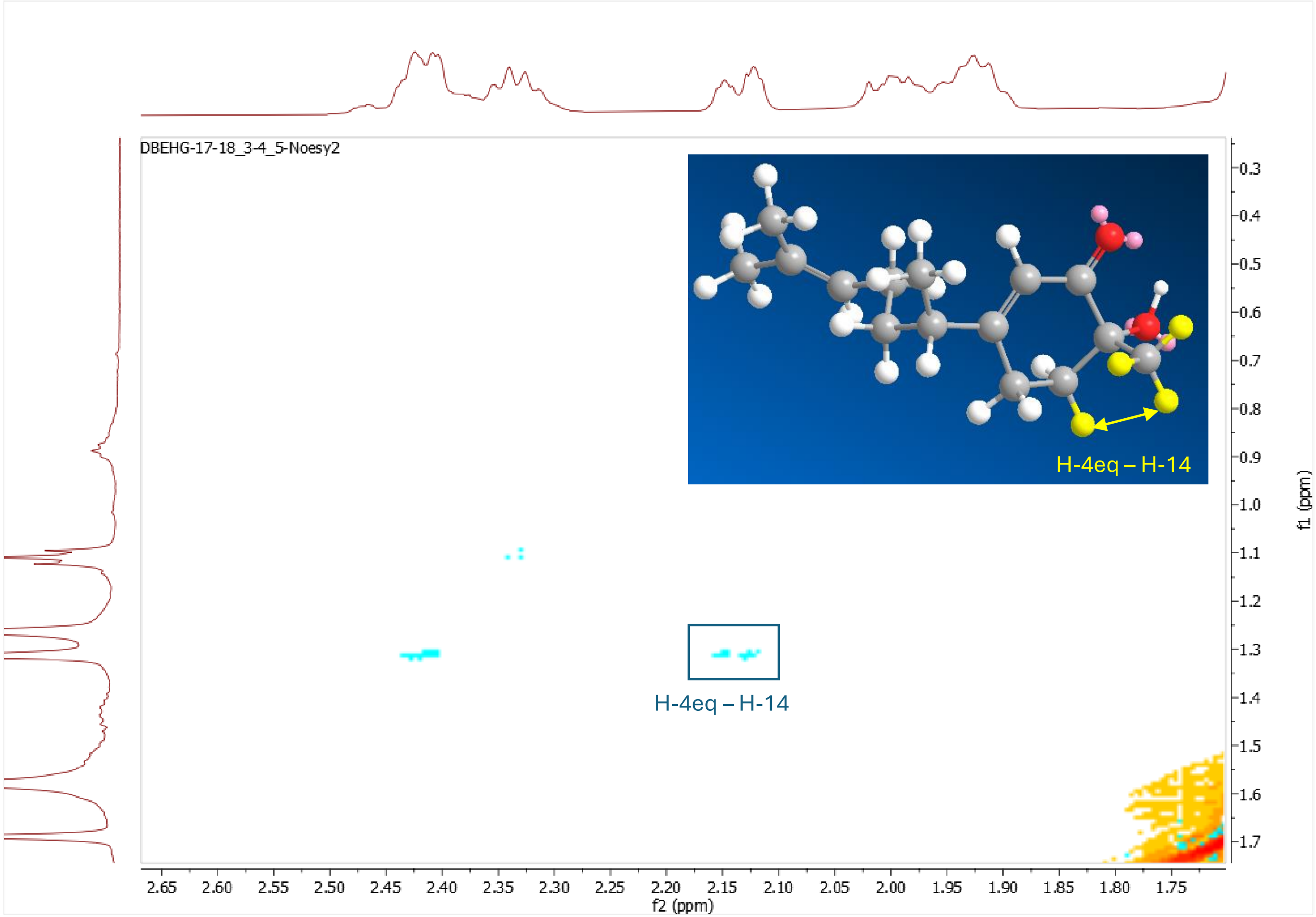

Figure S15. Expansion of NOESY correlation map of compound **1** (δ, $CDCl_3$, 500 MHz) and a figure representing a correlation between H-4$_{eq}$ and H-14.

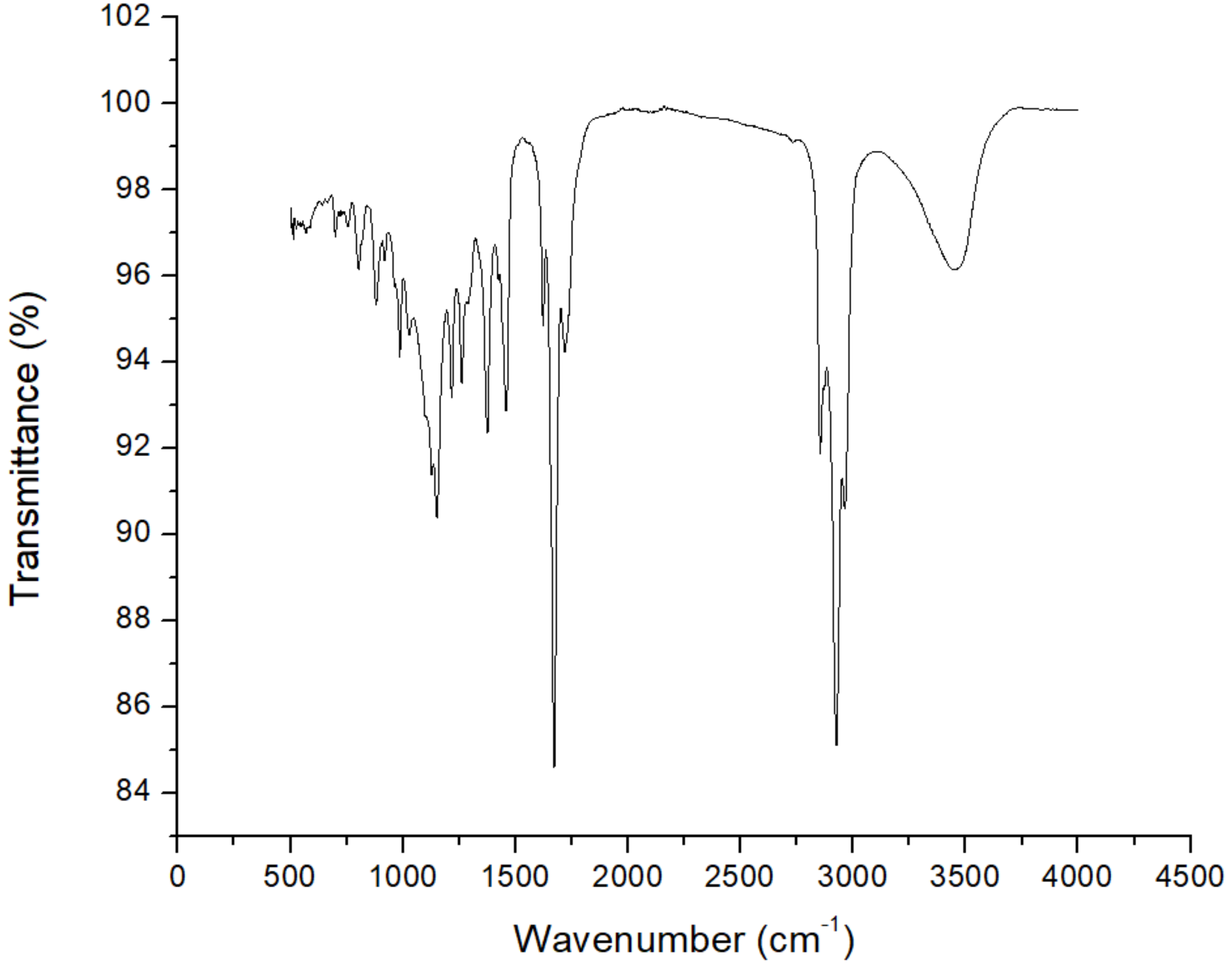

Figure S16. IR spectrum of compound **1**

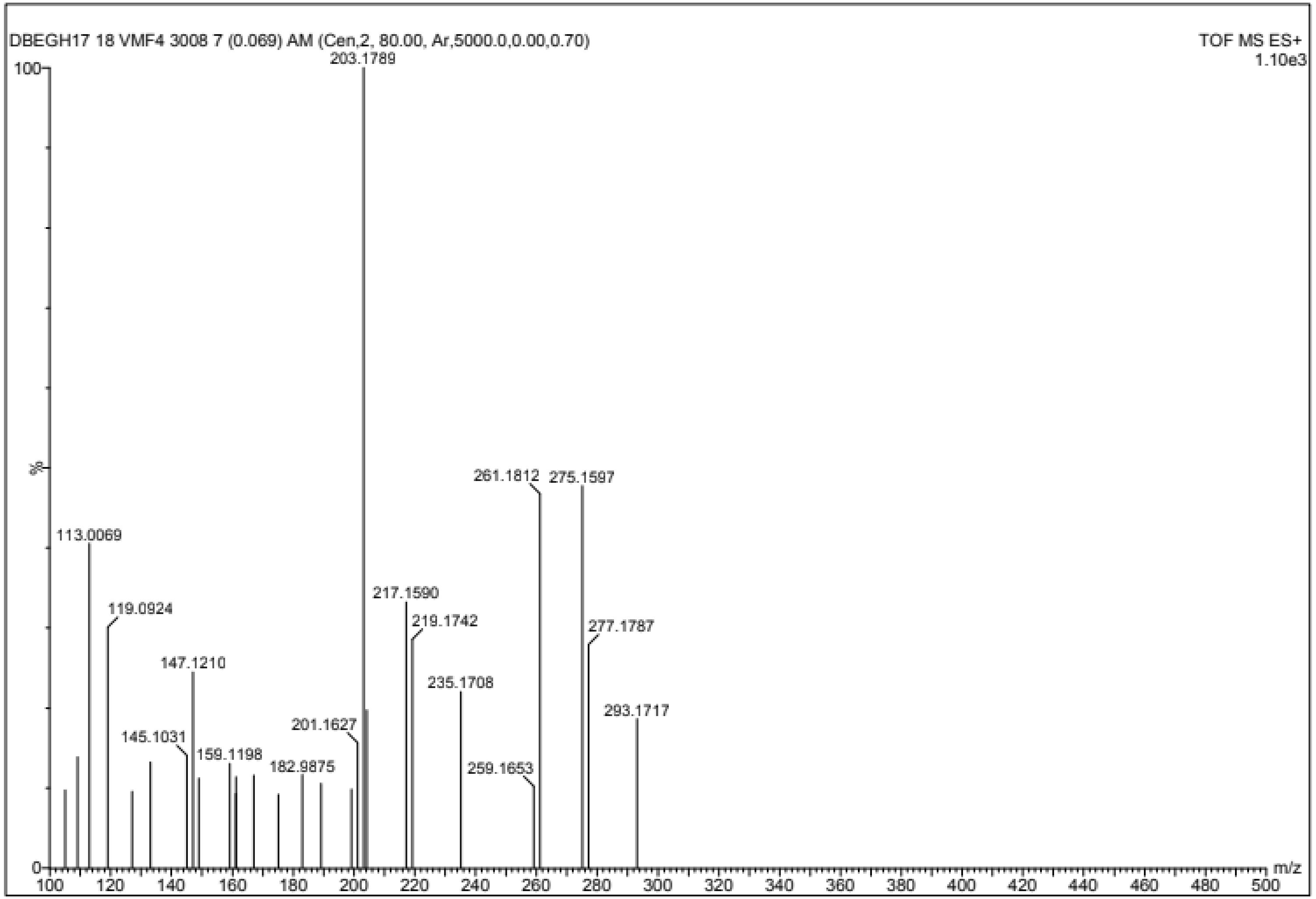

Figure S17. ESI-HRMS spectrum of compound **2**

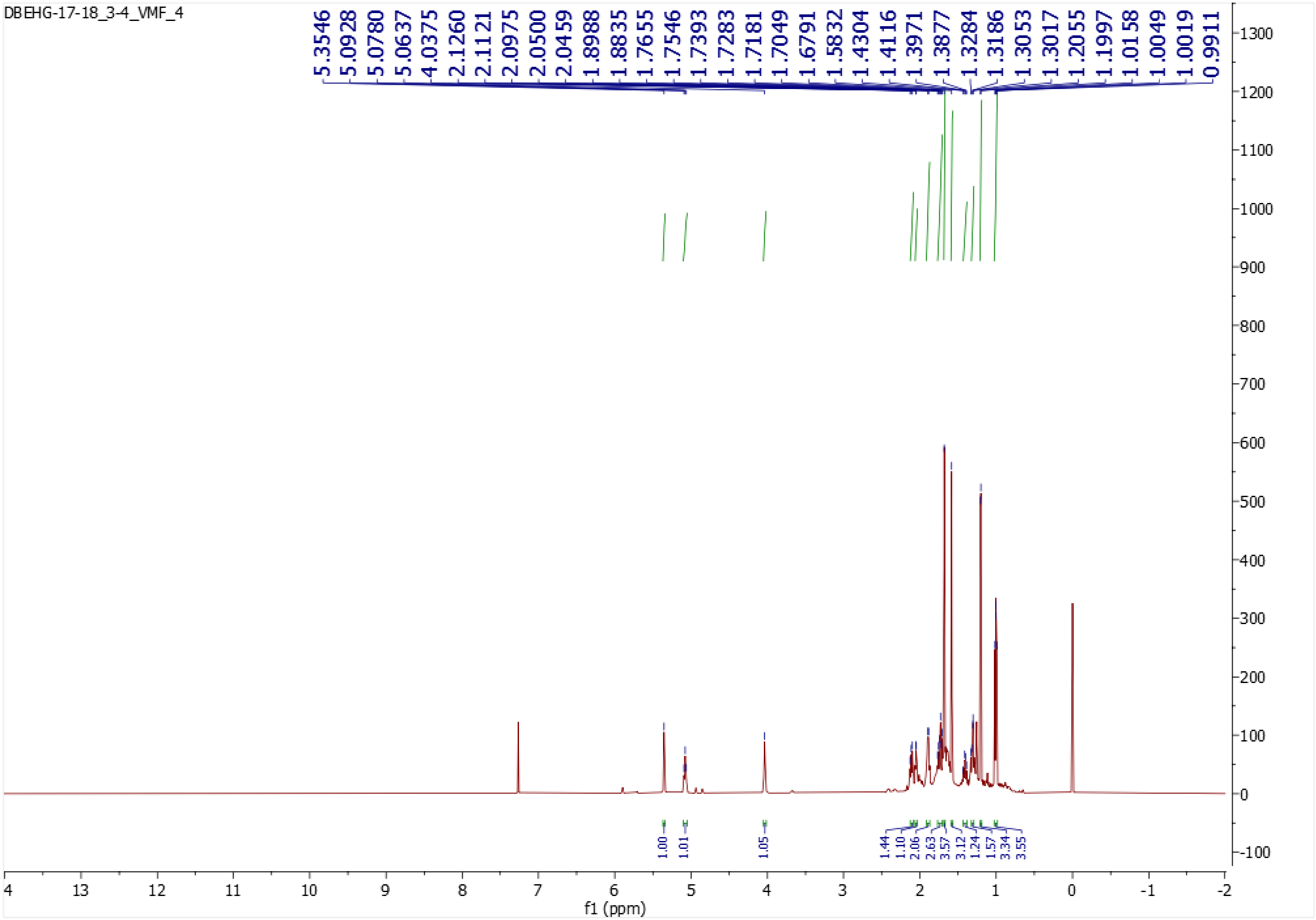

Figure S18. ^{1}H NMR spectrum of compound **2** (δ, $CDCl_3$, 500 MHz)

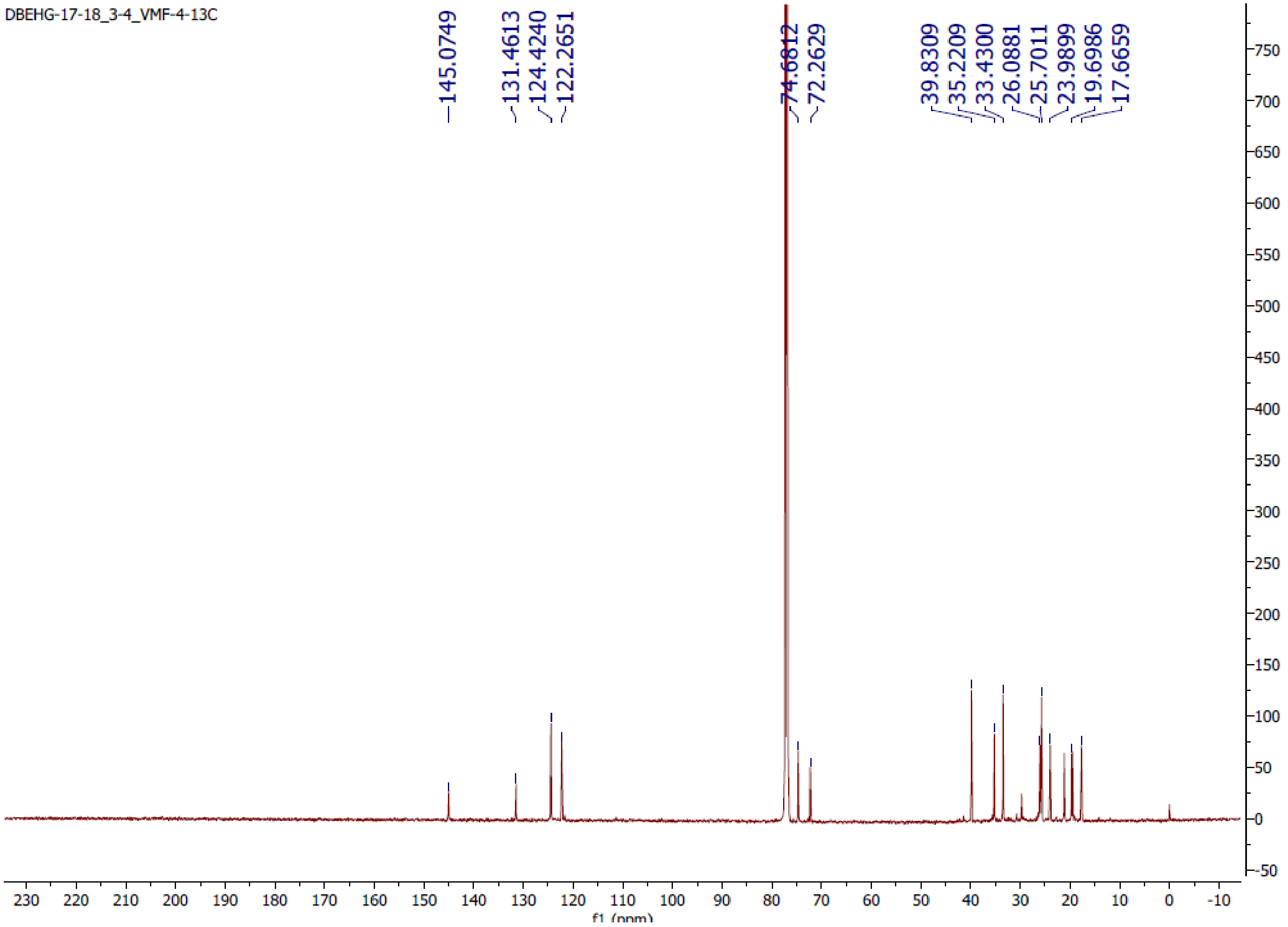

Figure S19. ^{13}C NMR spectrum of compounds **2** (δ, $CDCl_3$, 125 MHz)

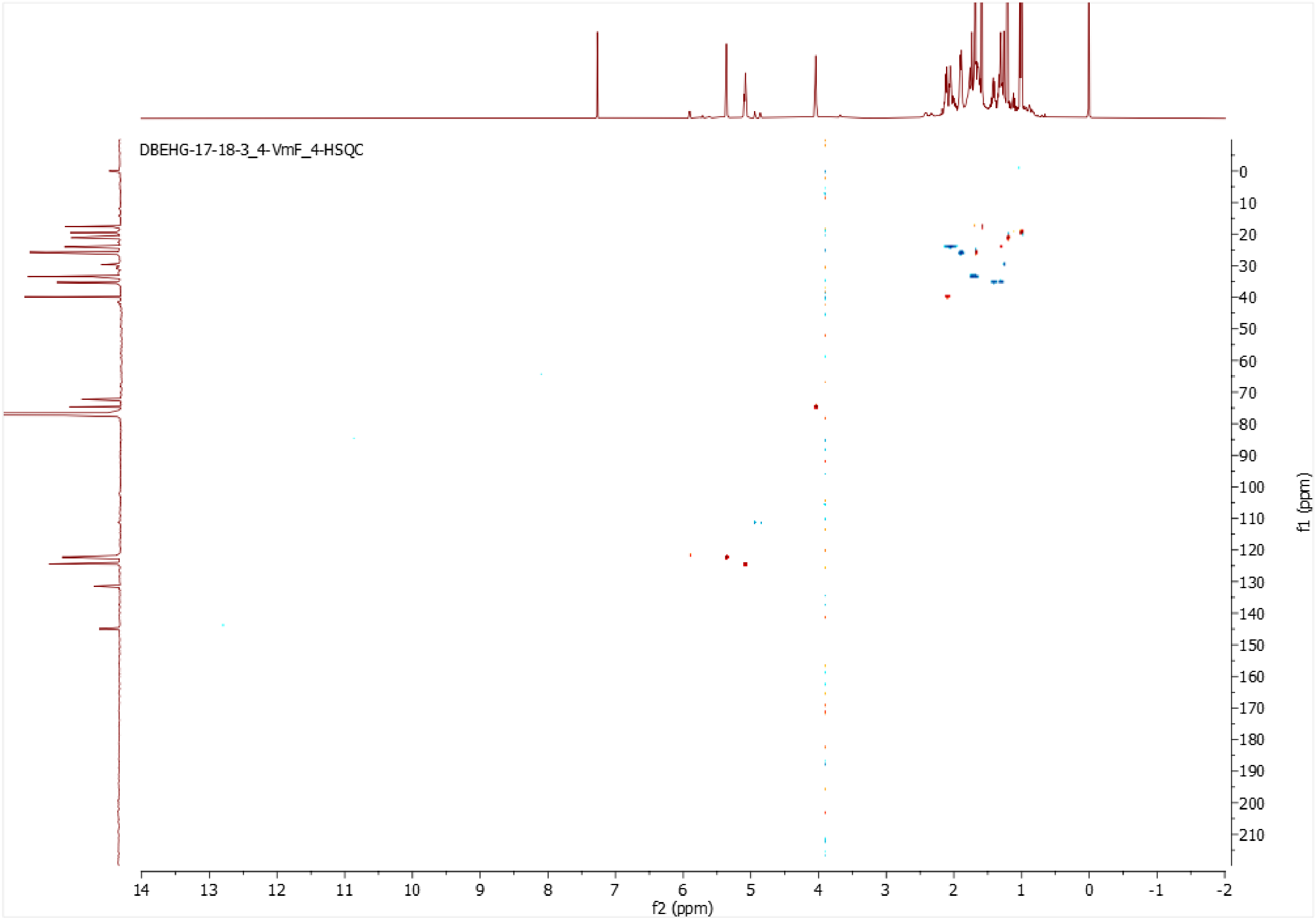

Figure S20. HSQC correlation map of compound **2** (δ, $CDCl_3$, 500 and 125 MHz)

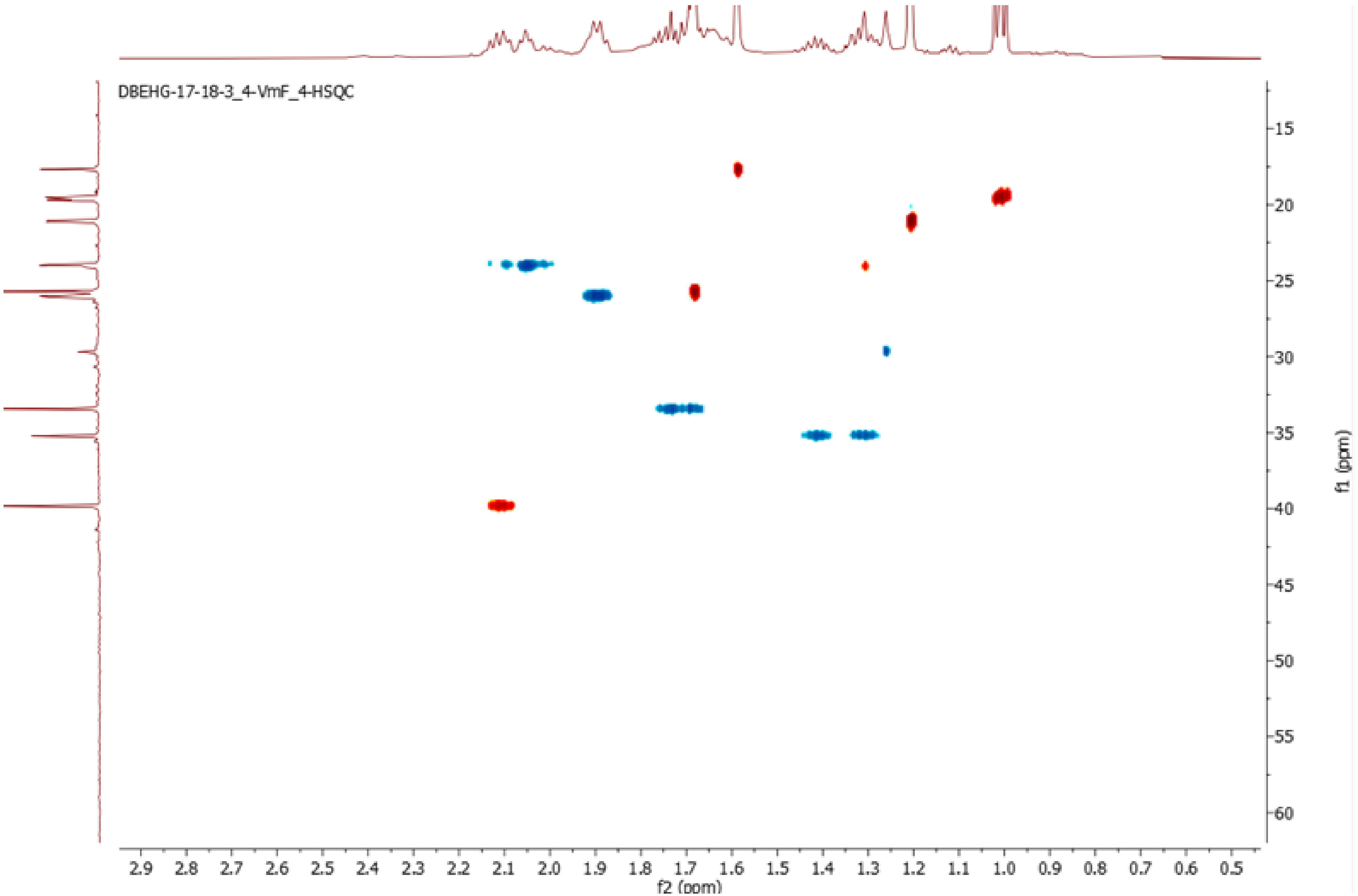

Figure S21. Expansion of HSQC correlation map of compound **2** (δ, $CDCl_3$, 500 and 125 MHz)

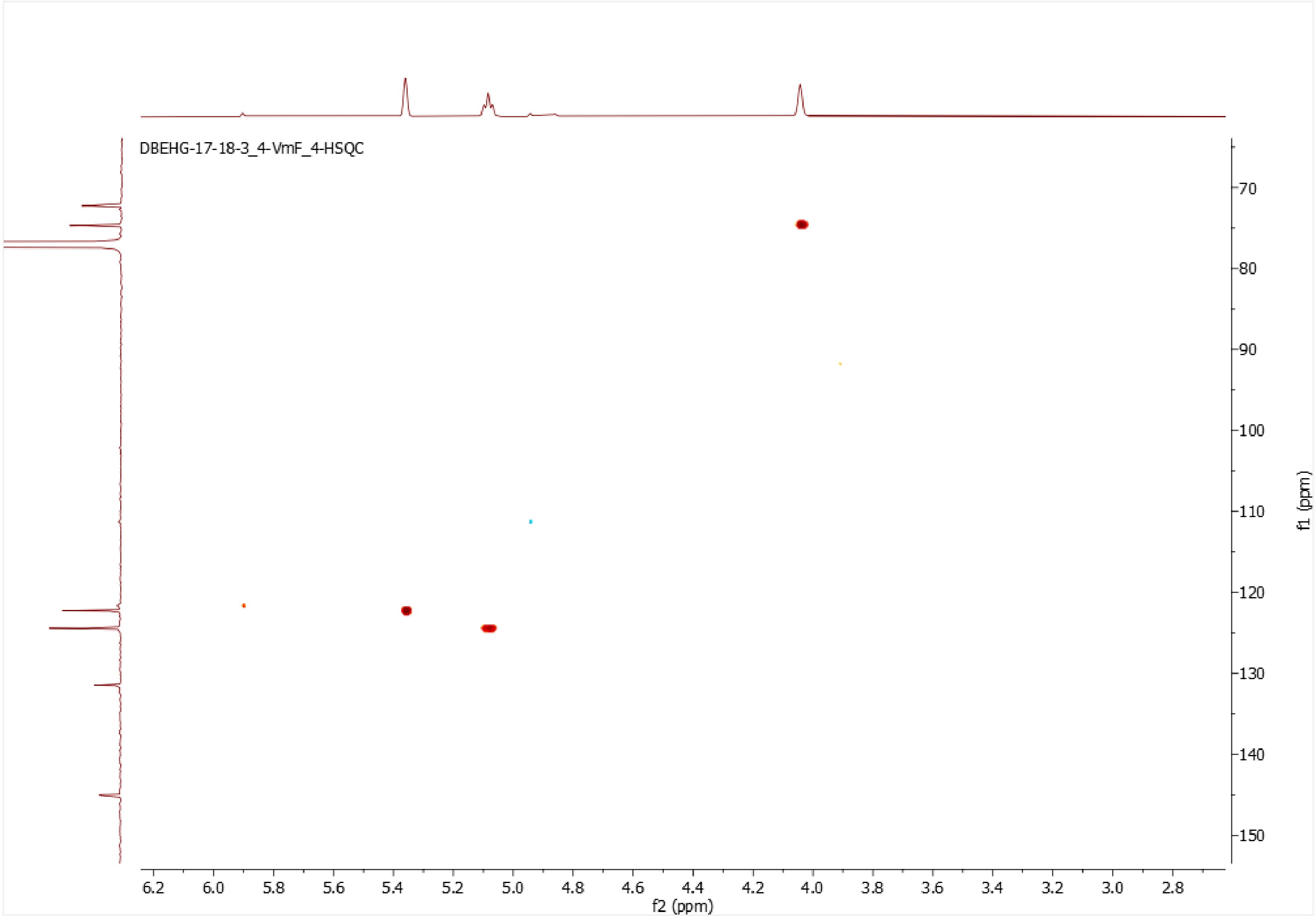

Figure S22. Expansion of HSQC correlation map of compound **2** (δ, $CDCl_3$, 500 and 125 MHz)

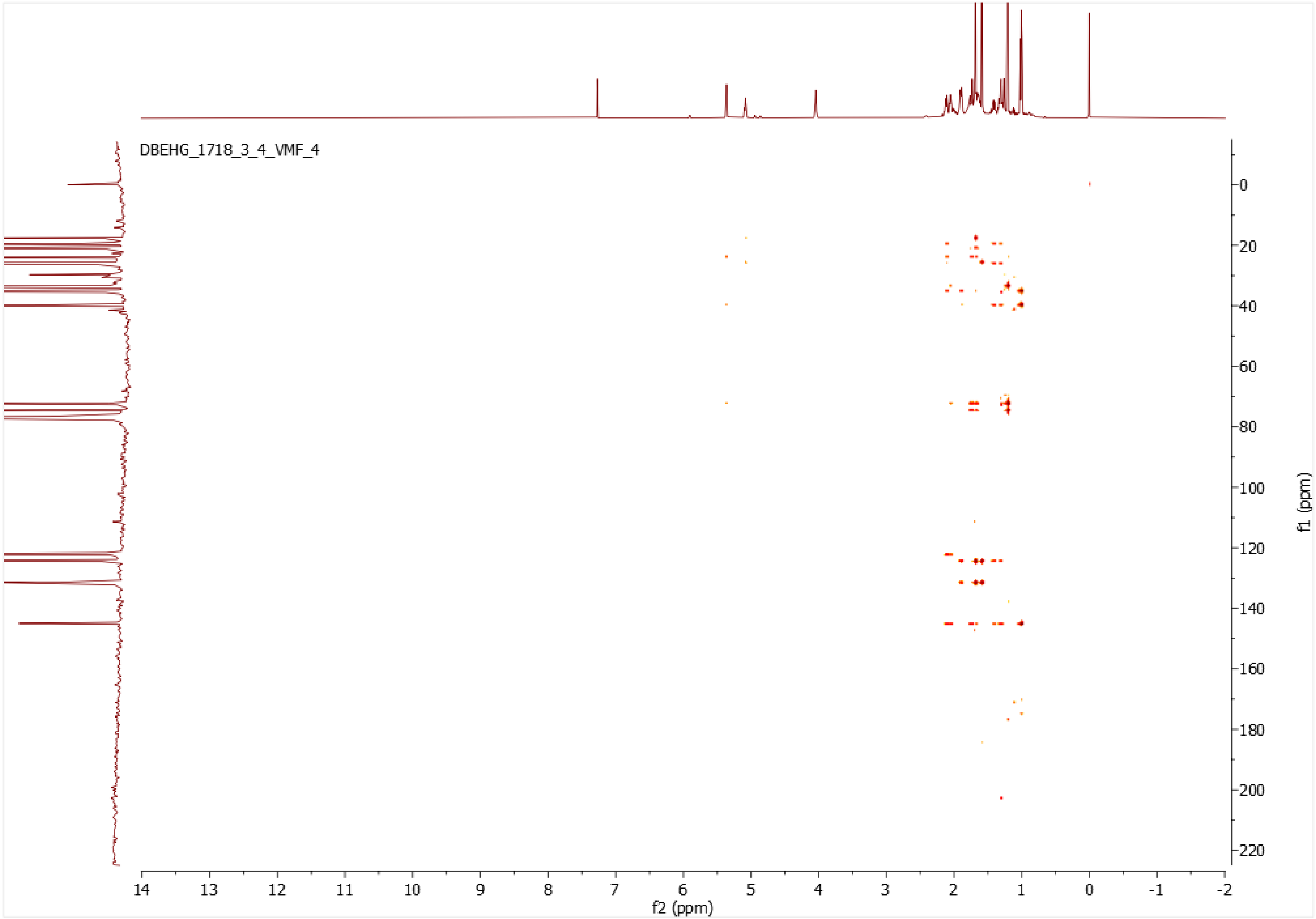

Figure S23. HMBC correlation map of compound **2** (δ, $CDCl_3$, 500 and 125 MHz)

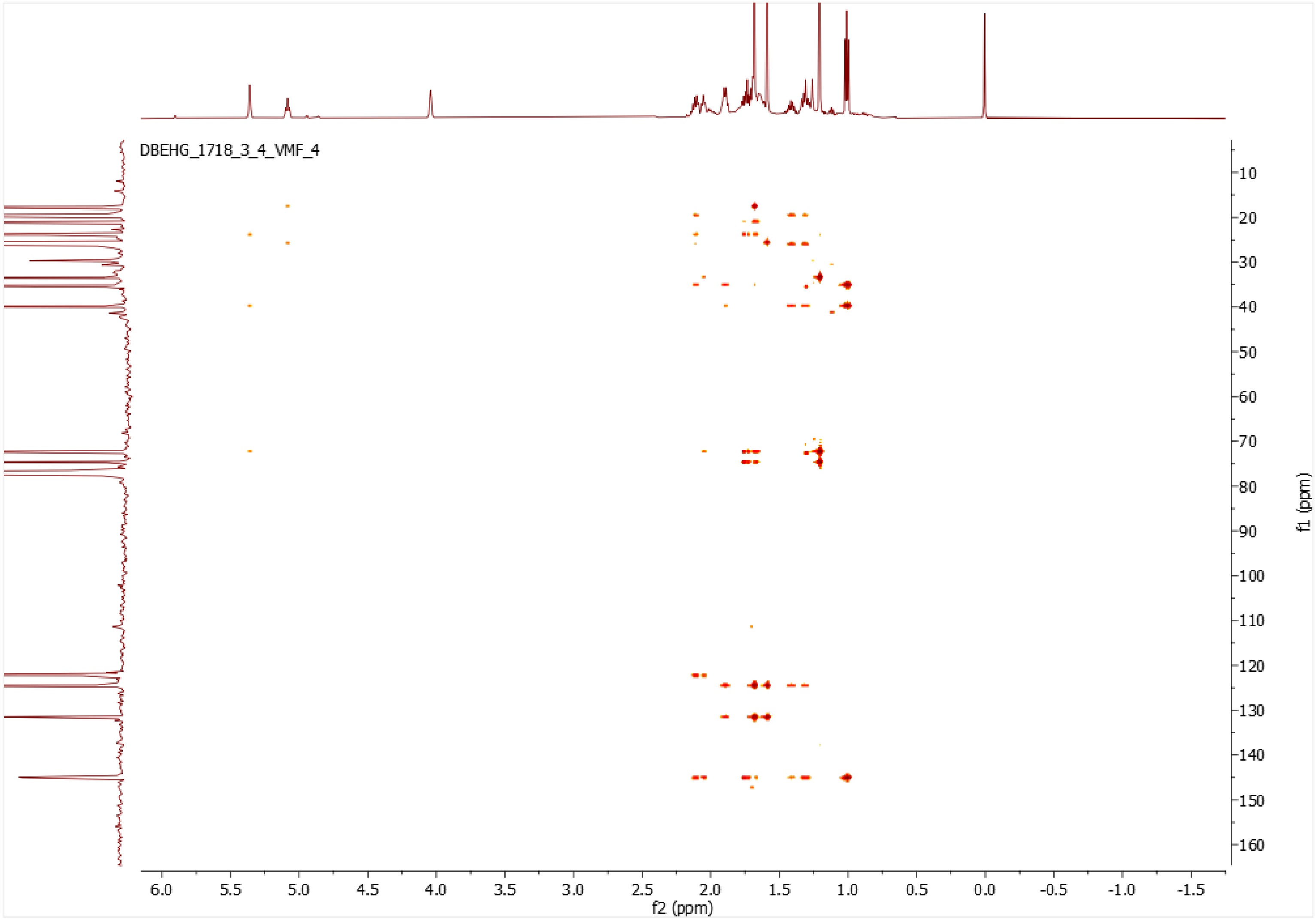

Figure S24. Expansion of HSQC correlation map of compound **2** (δ, $CDCl_3$, 500 and 125 MHz)

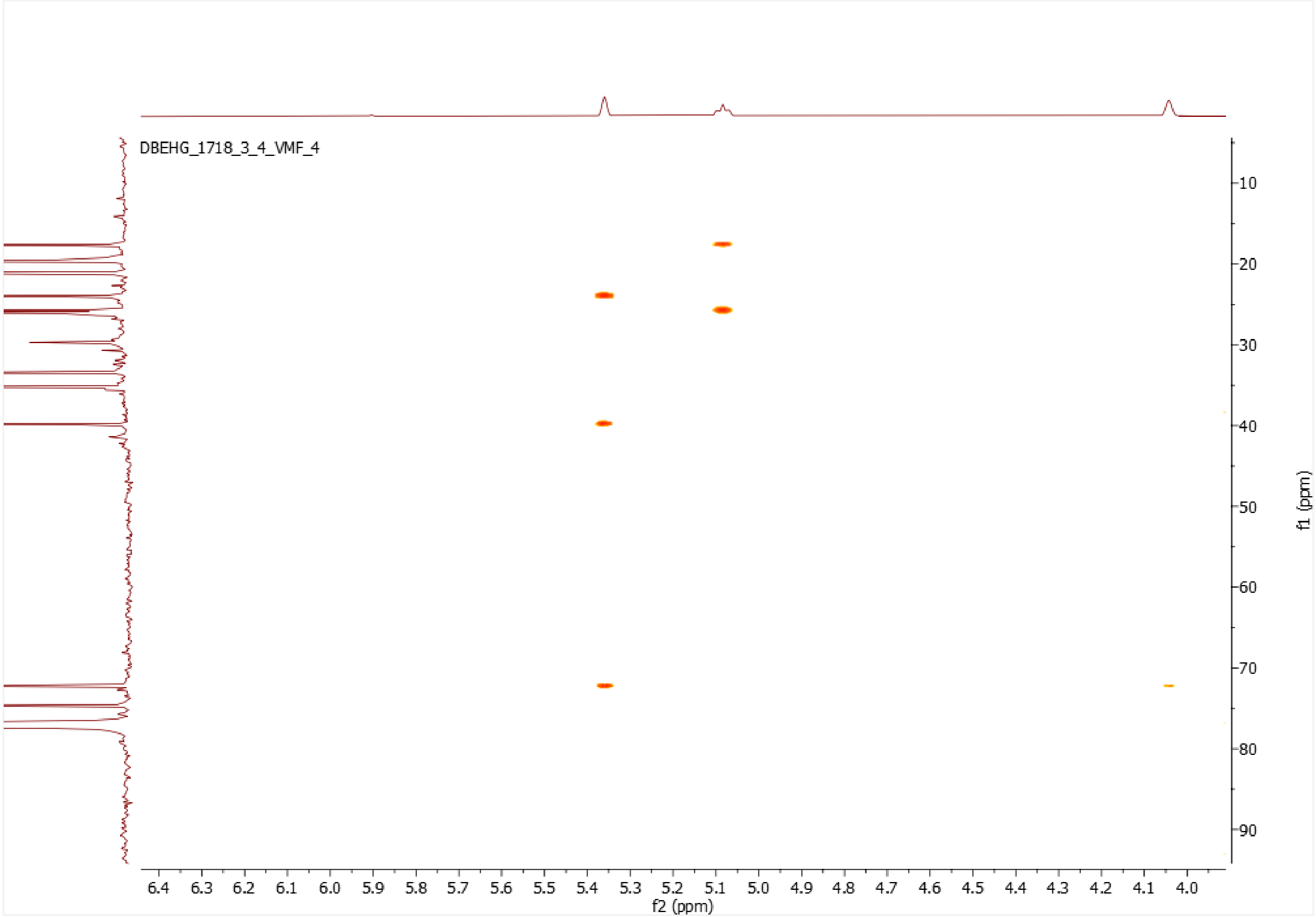

Figure S25. Expansion of HMBC correlation map of compound **2** (δ, $CDCl_3$, 500 and 125 MHz)

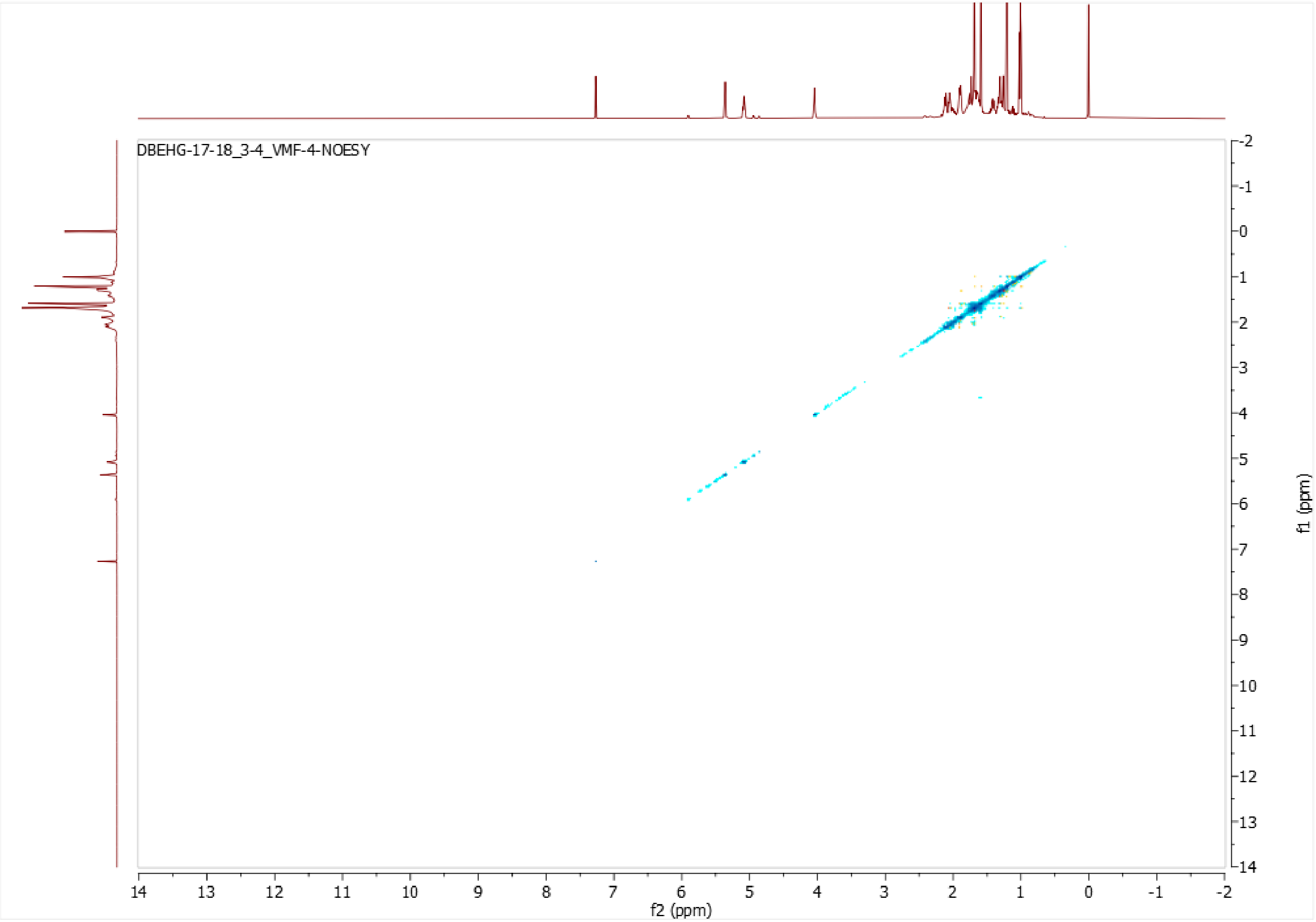

Figure S26. NOESY correlation map of compound **2** (δ, $CDCl_3$, 500 MHz)

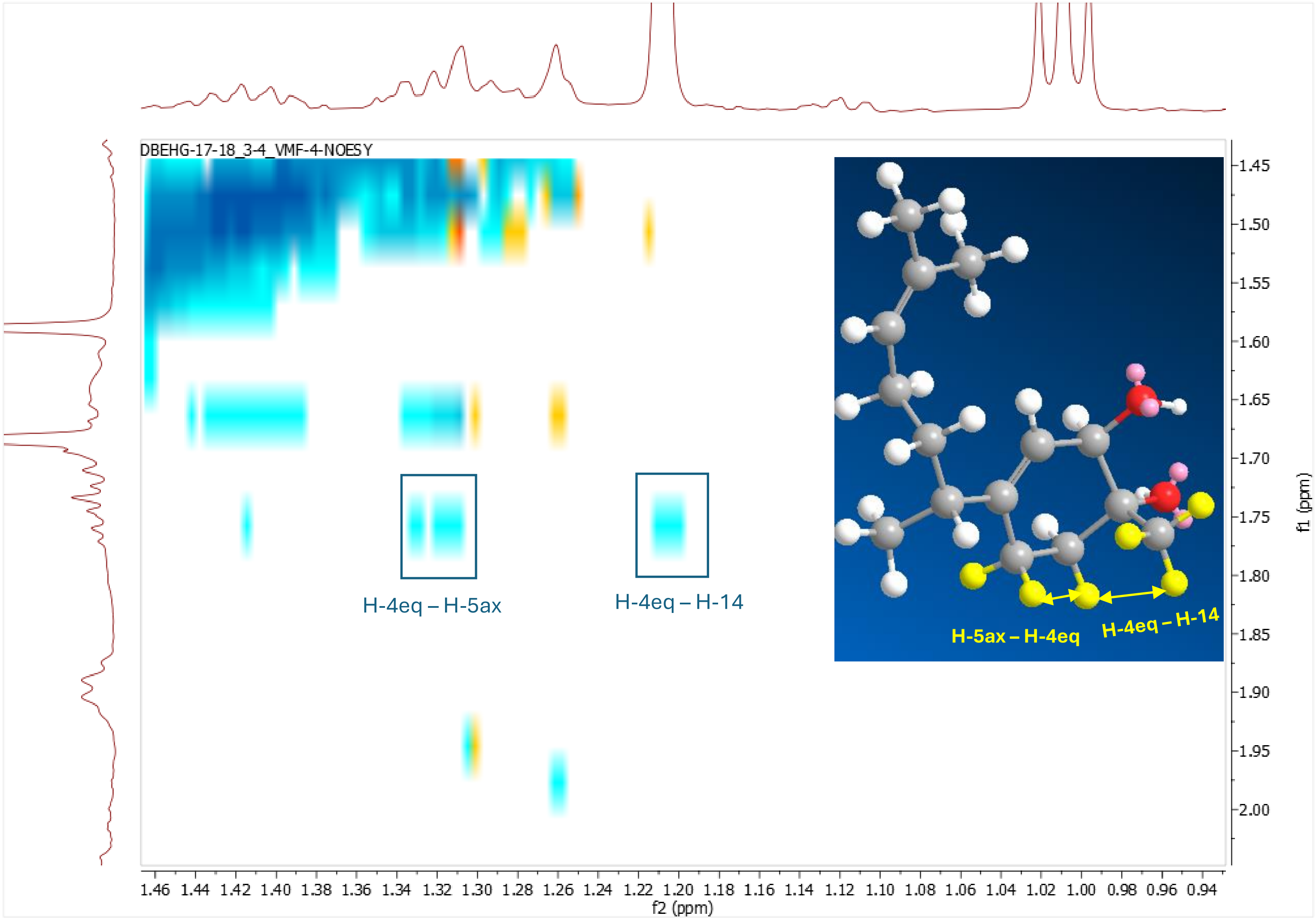

Figure S27. Expansion of NOESY correlation map of compound **2** (δ, $CDCl_3$, 500 MHz) and a figure representing a correlation between H-4_{eq} and H-5_{ax} and between H-4_{eq} and H-14.

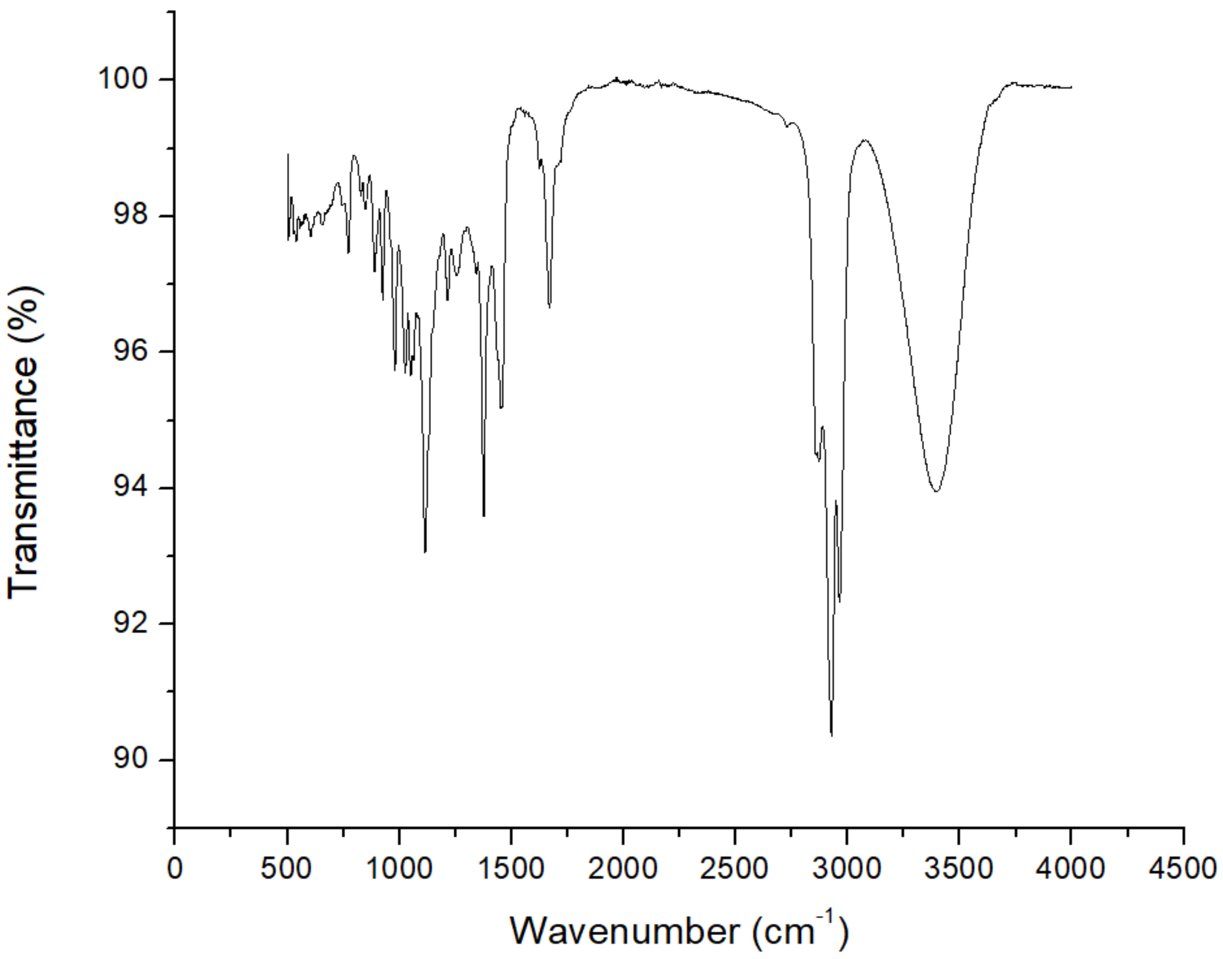

Figure S28. IR spectrum of compound **2**

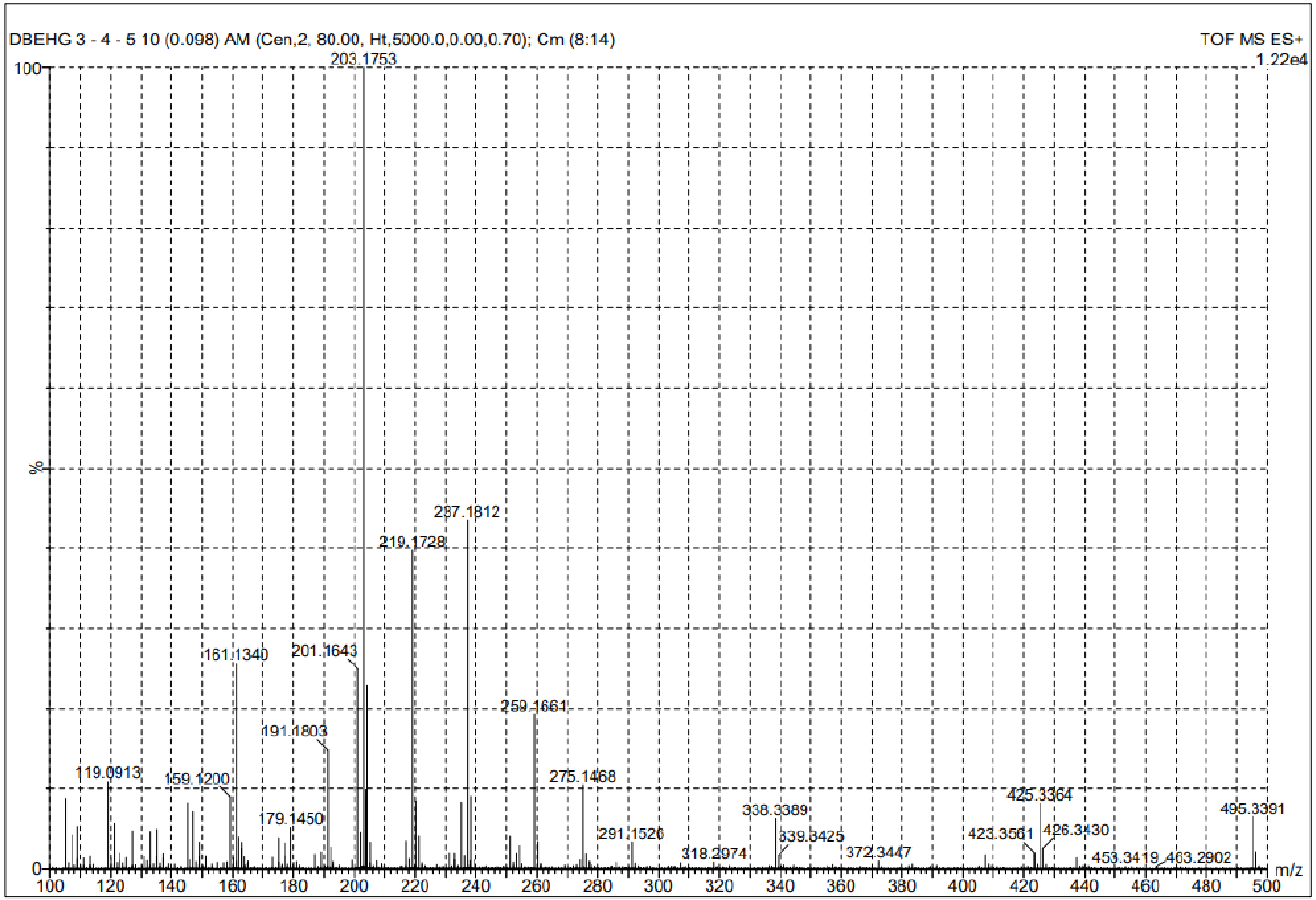

Figure S29. ESI-HRMS spectrum of compound **3**

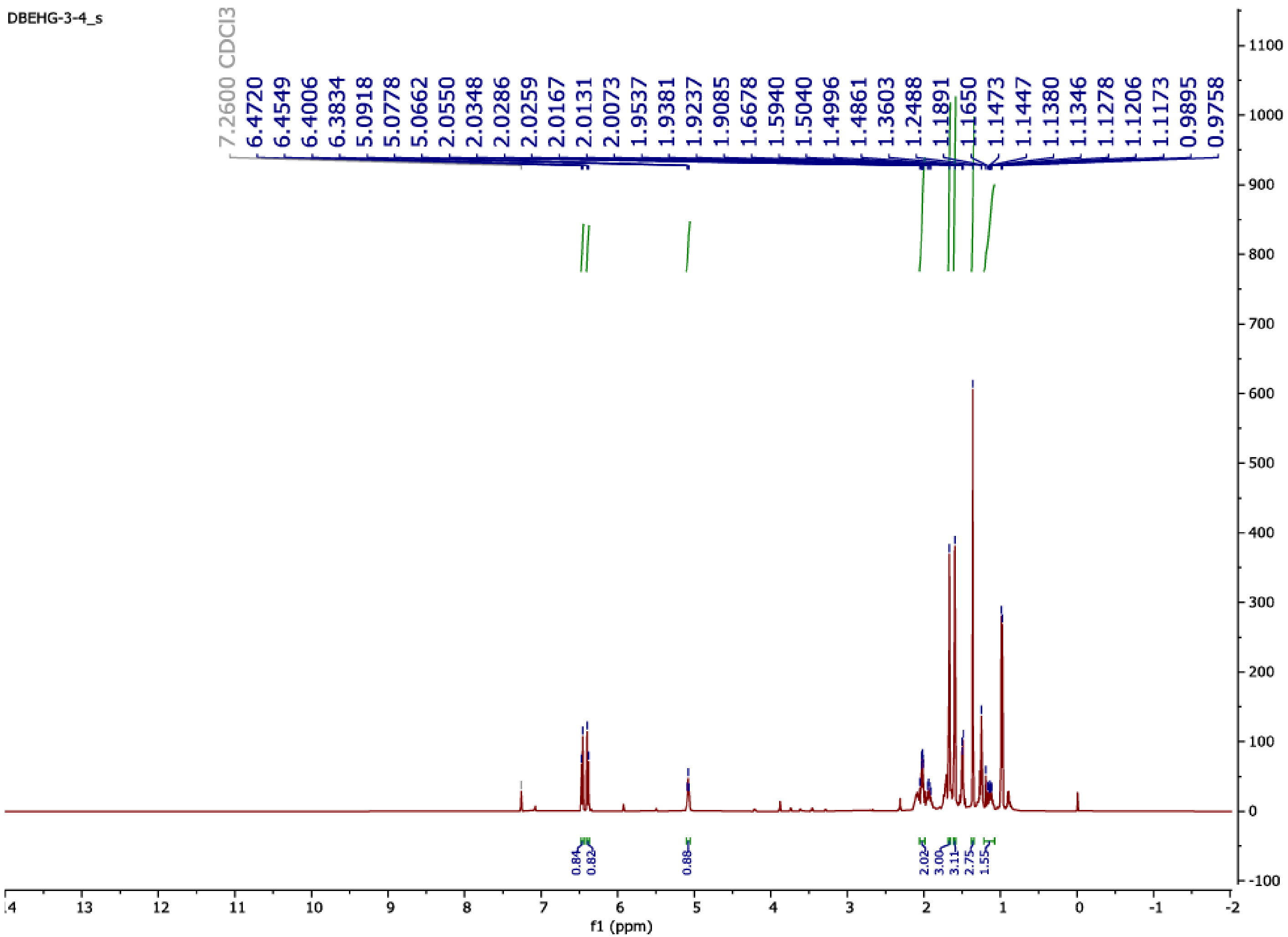

Figure S30. ^{1}H NMR spectrum of compound **3** (δ, $CDCl_3$, 500 MHz)

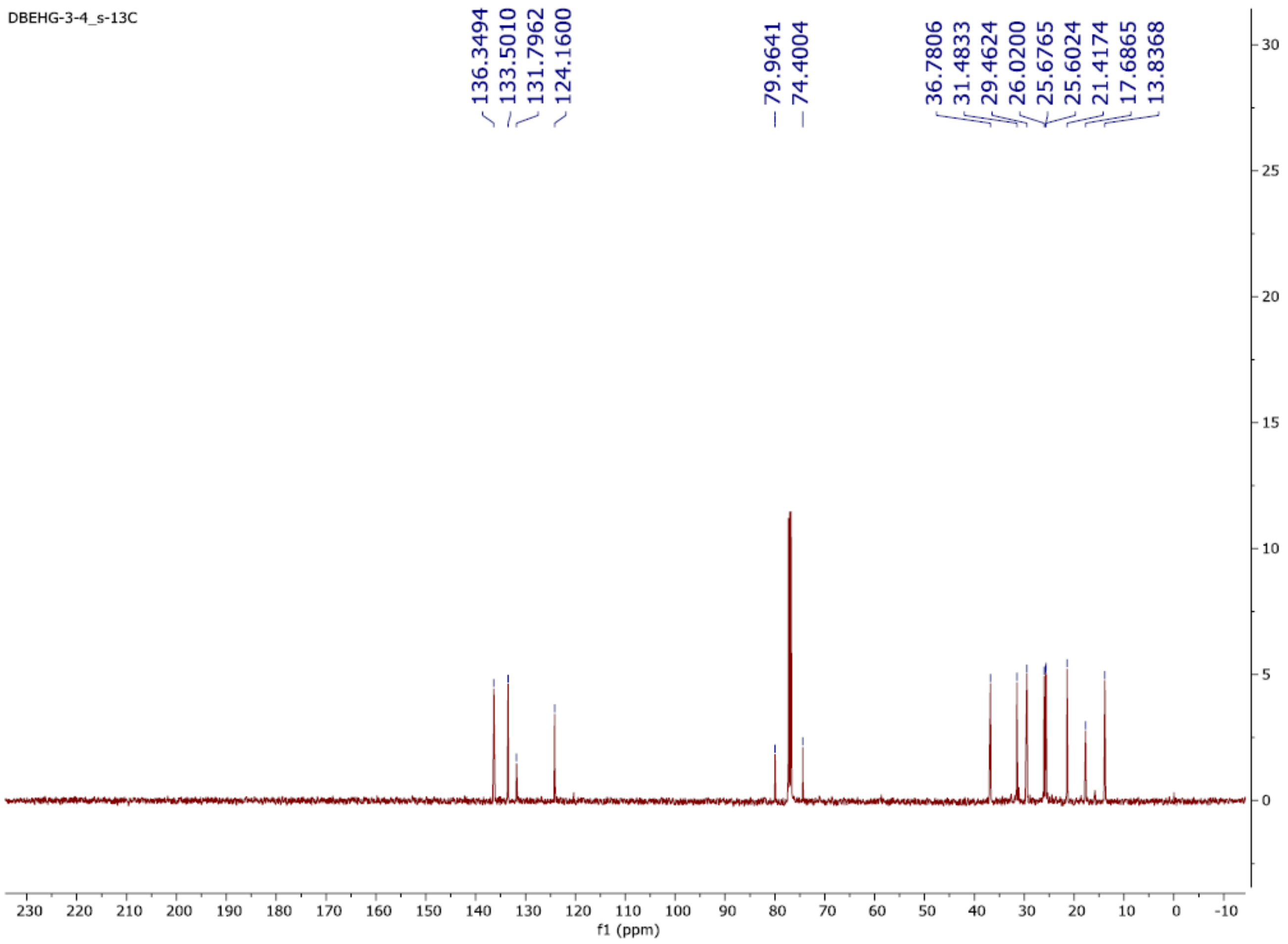

Figure S31. ^{13}C NMR spectrum of compound **3** (δ, $CDCl_3$, 125 MHz)

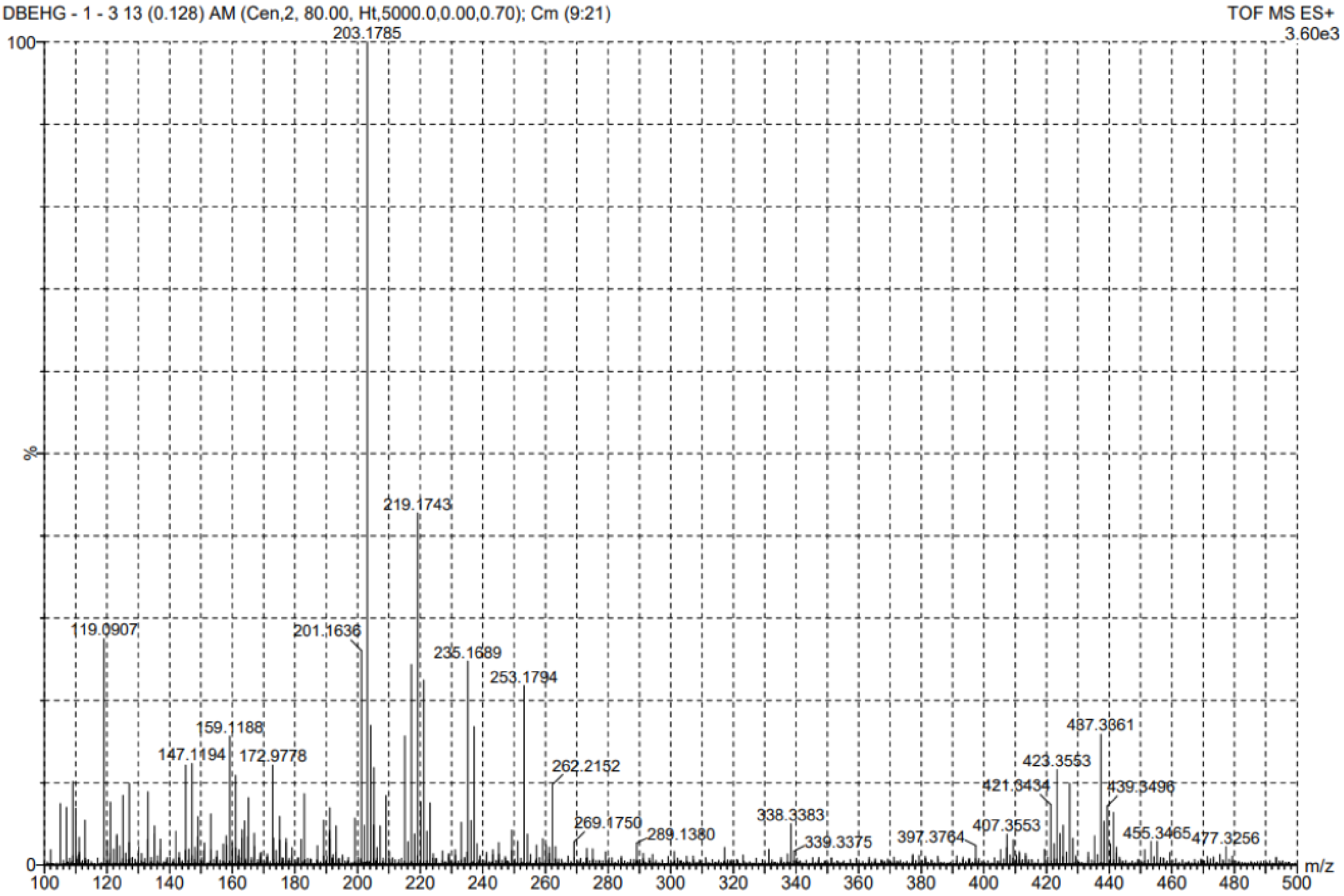

Figure S32. ESI-HRMS spectrum of compound **4**

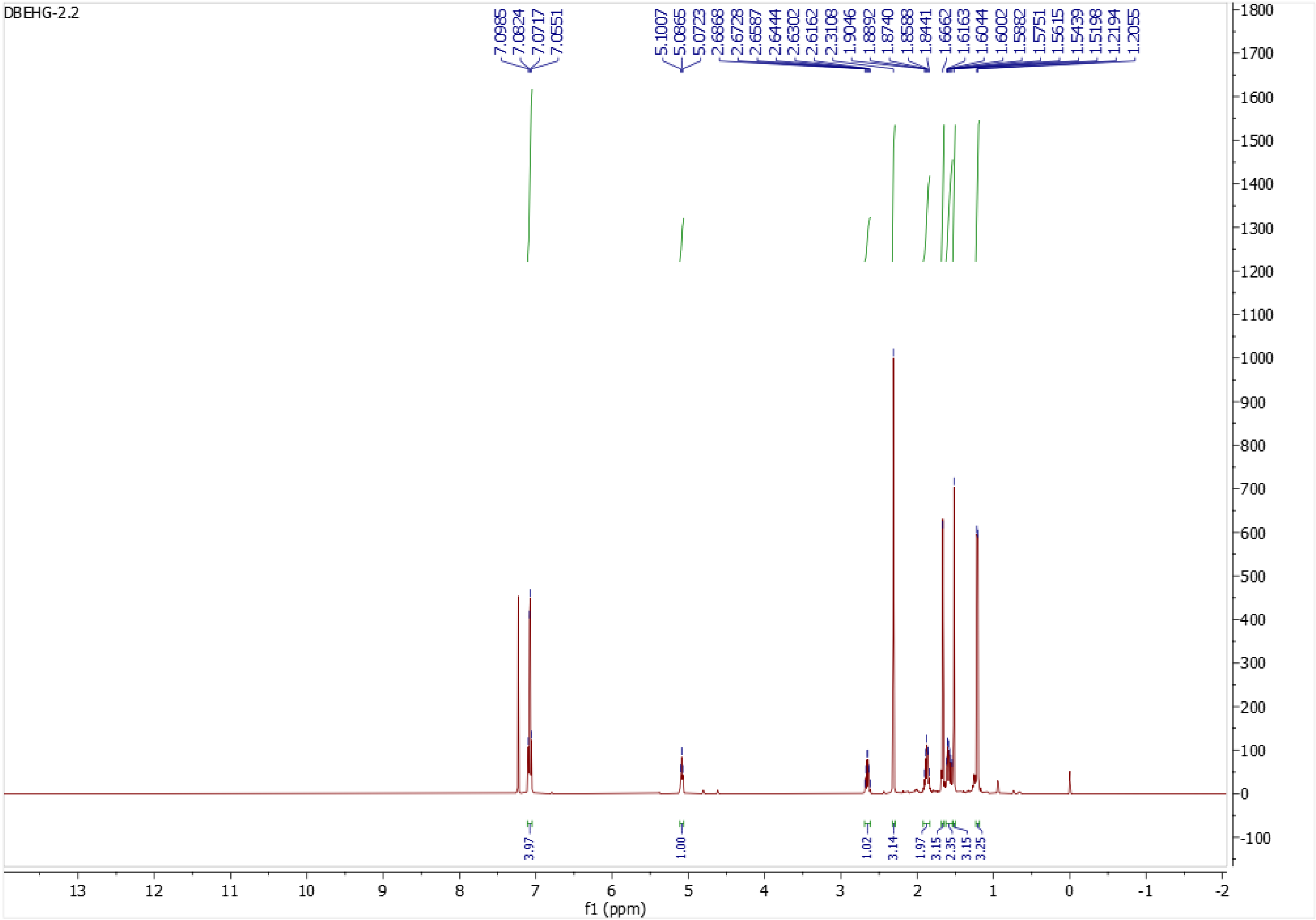

Figure S33. ^{1}H NMR spectrum of compound **4** (δ, $CDCl_3$, 500 MHz)

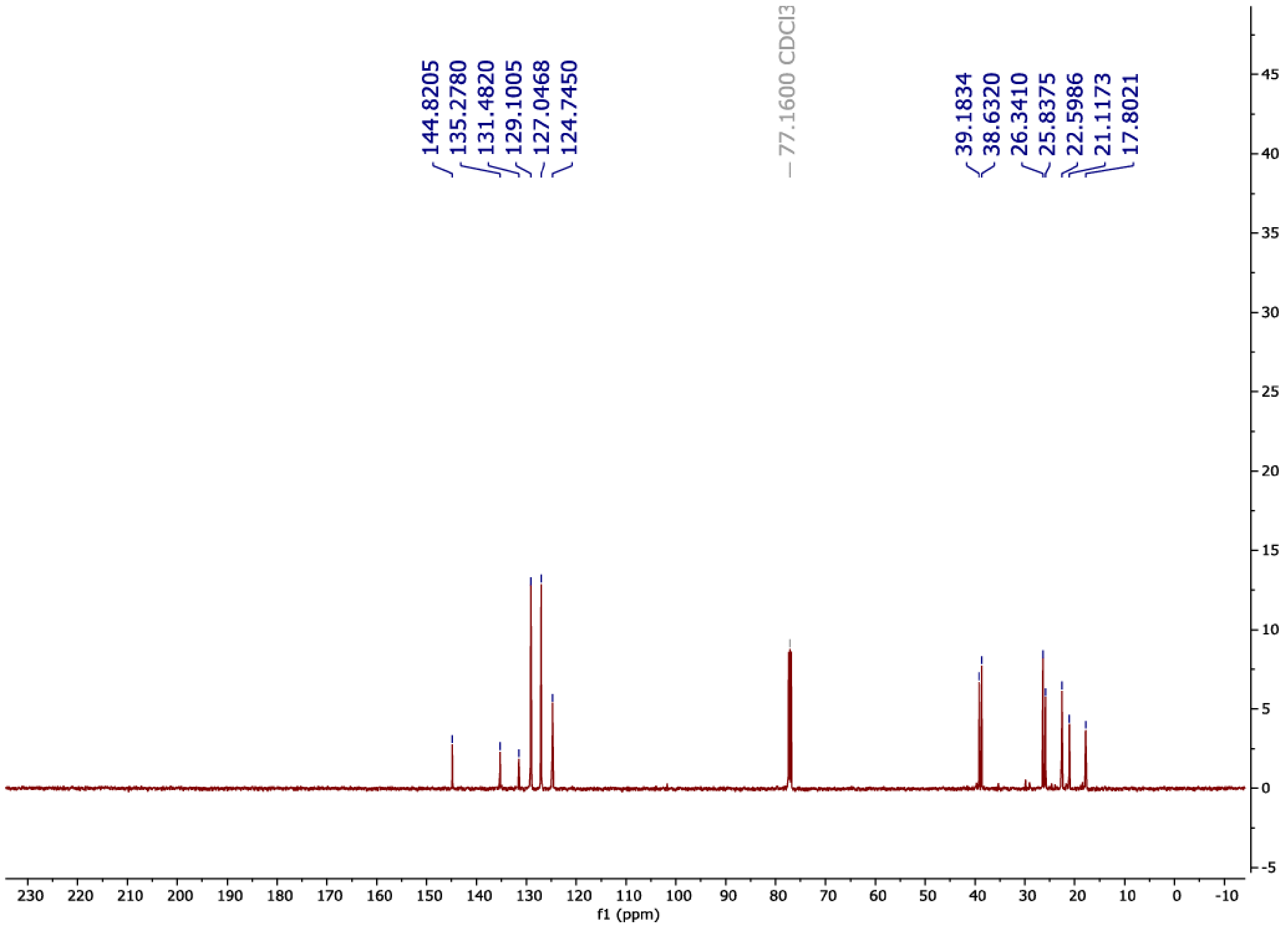

Figure S34. ^{13}C NMR spectrum of compound **4** (δ, $CDCl_3$, 125 MHz)

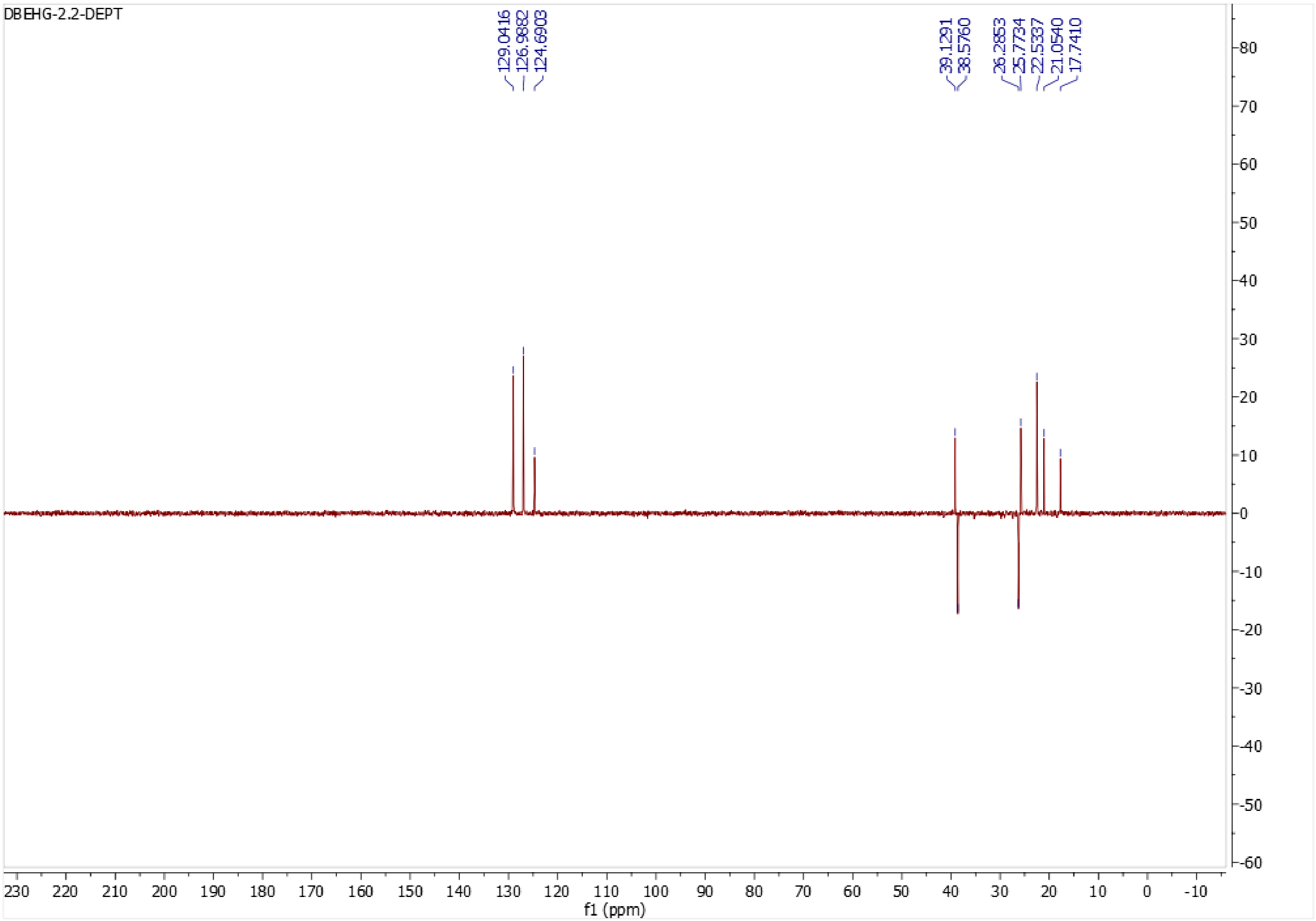

Figure S35. DEPT spectrum of compound **4** (δ, $CDCl_3$, 125 MHz)

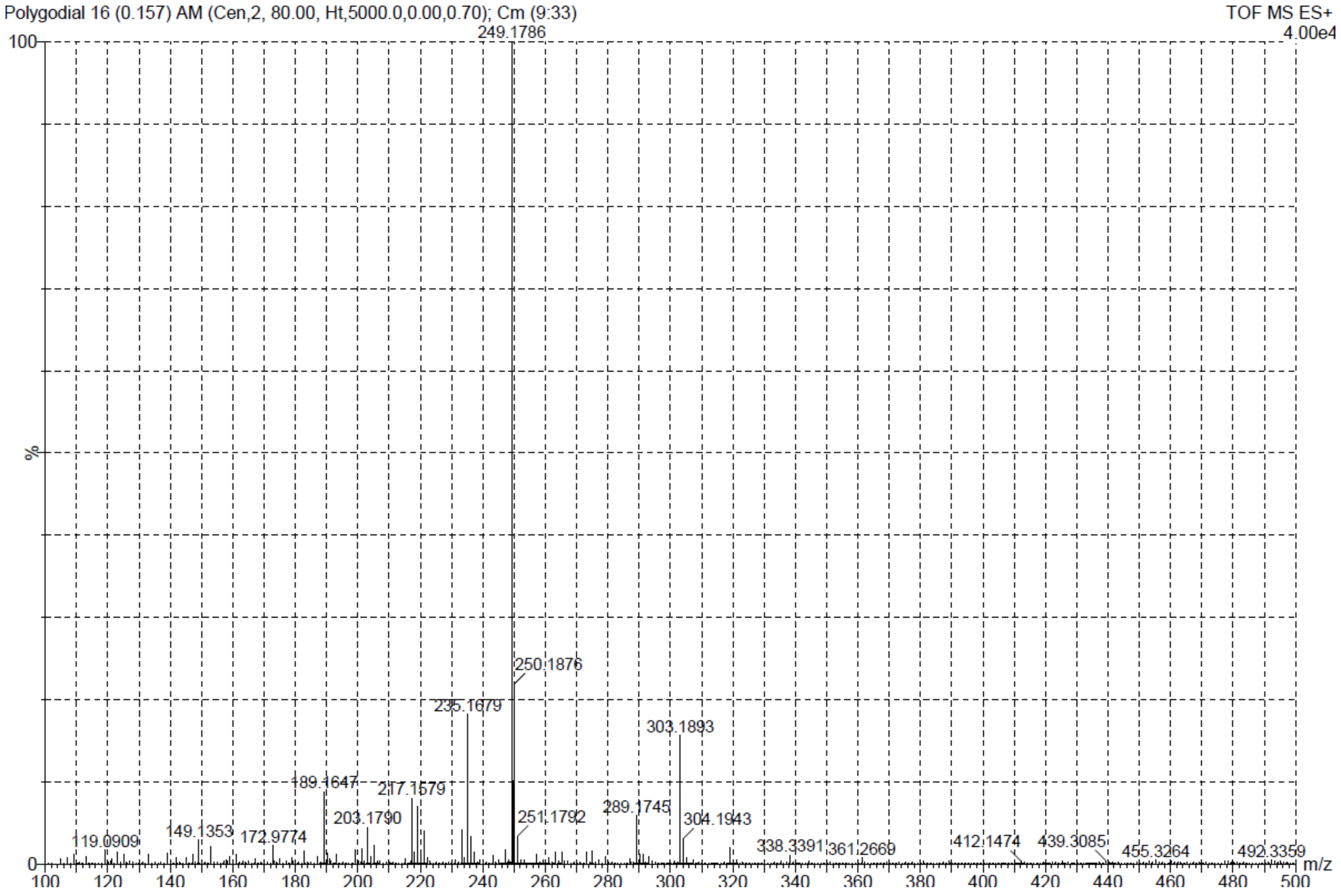

Figure S36. ESI-HRMS spectrum of compound **5**

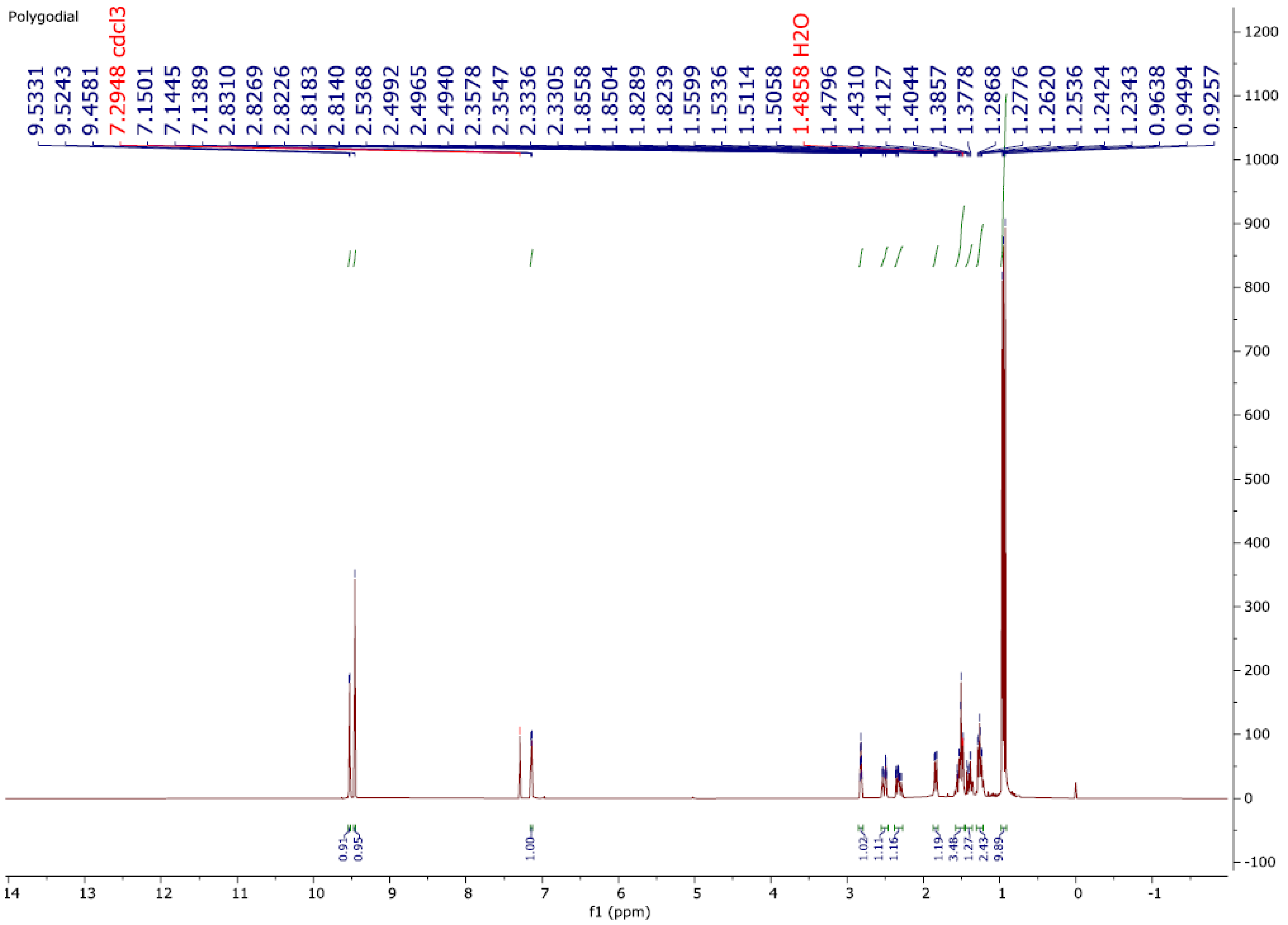

Figure S37. ^{1}H NMR spectrum of compound **5** (δ, $CDCl_3$, 500 MHz)

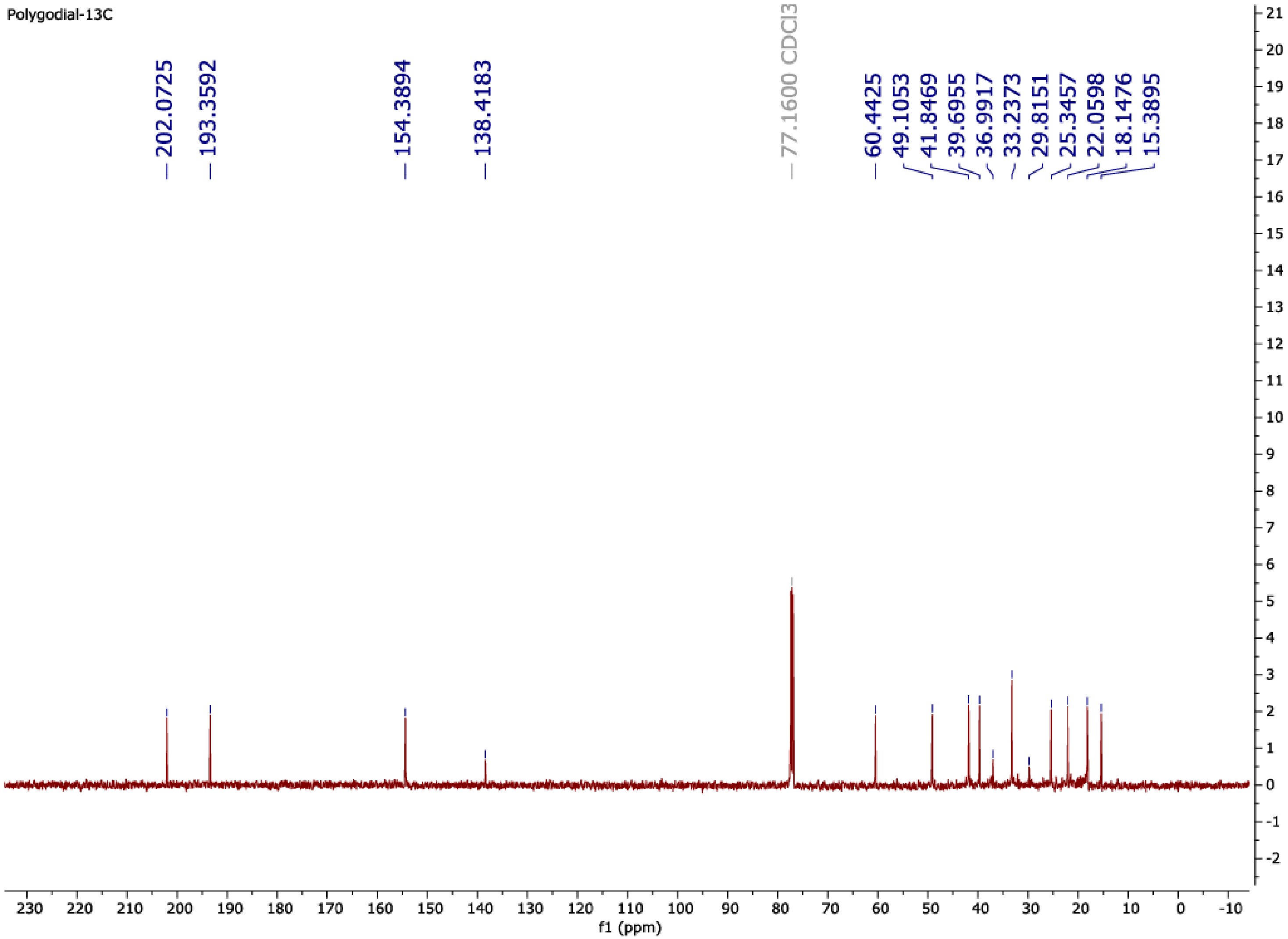

Figure S38. ^{13}C NMR spectrum of compound **5** (δ, $CDCl_3$, 125 MHz)

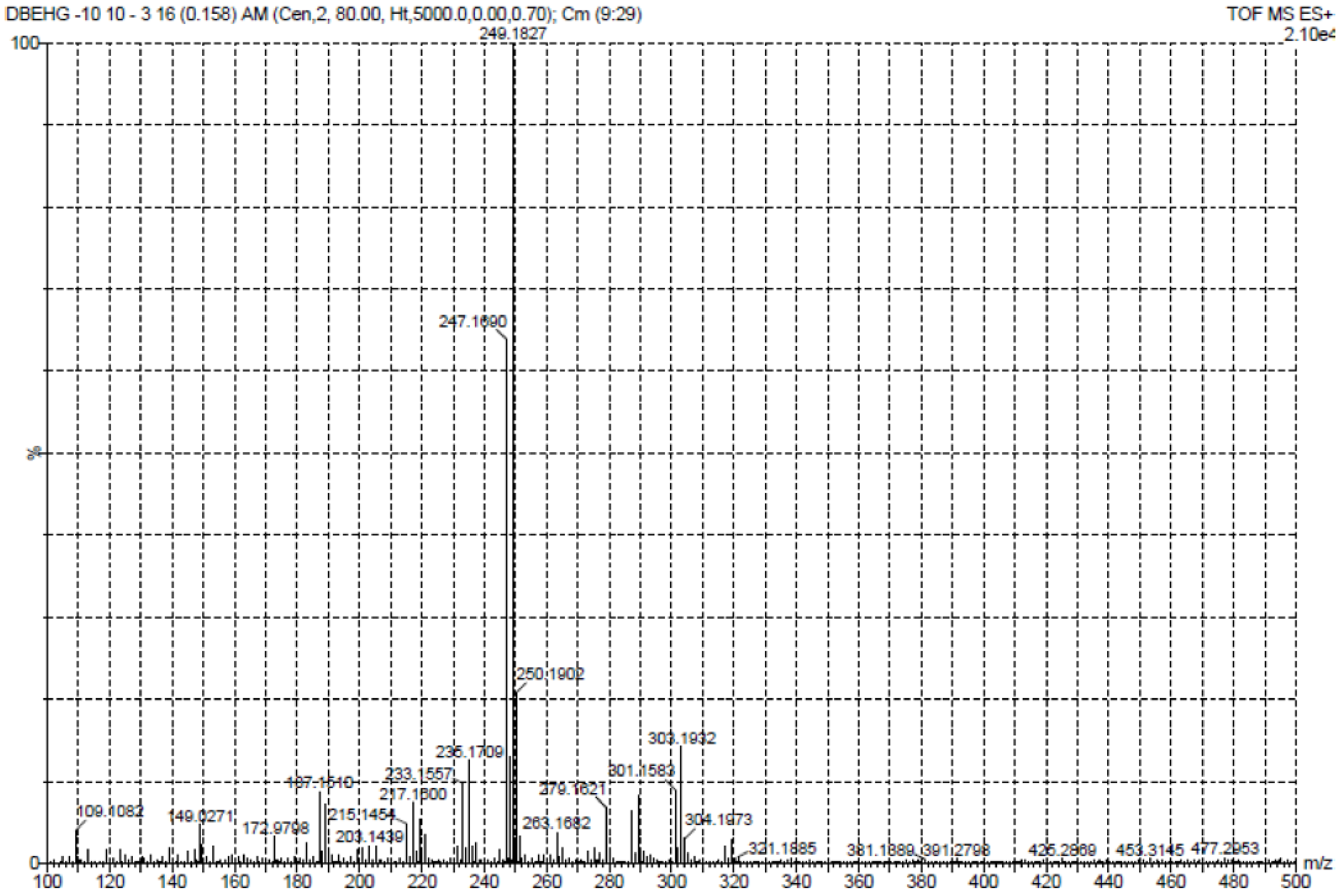

Figure S39. ESI-HRMS spectrum of compound **6**

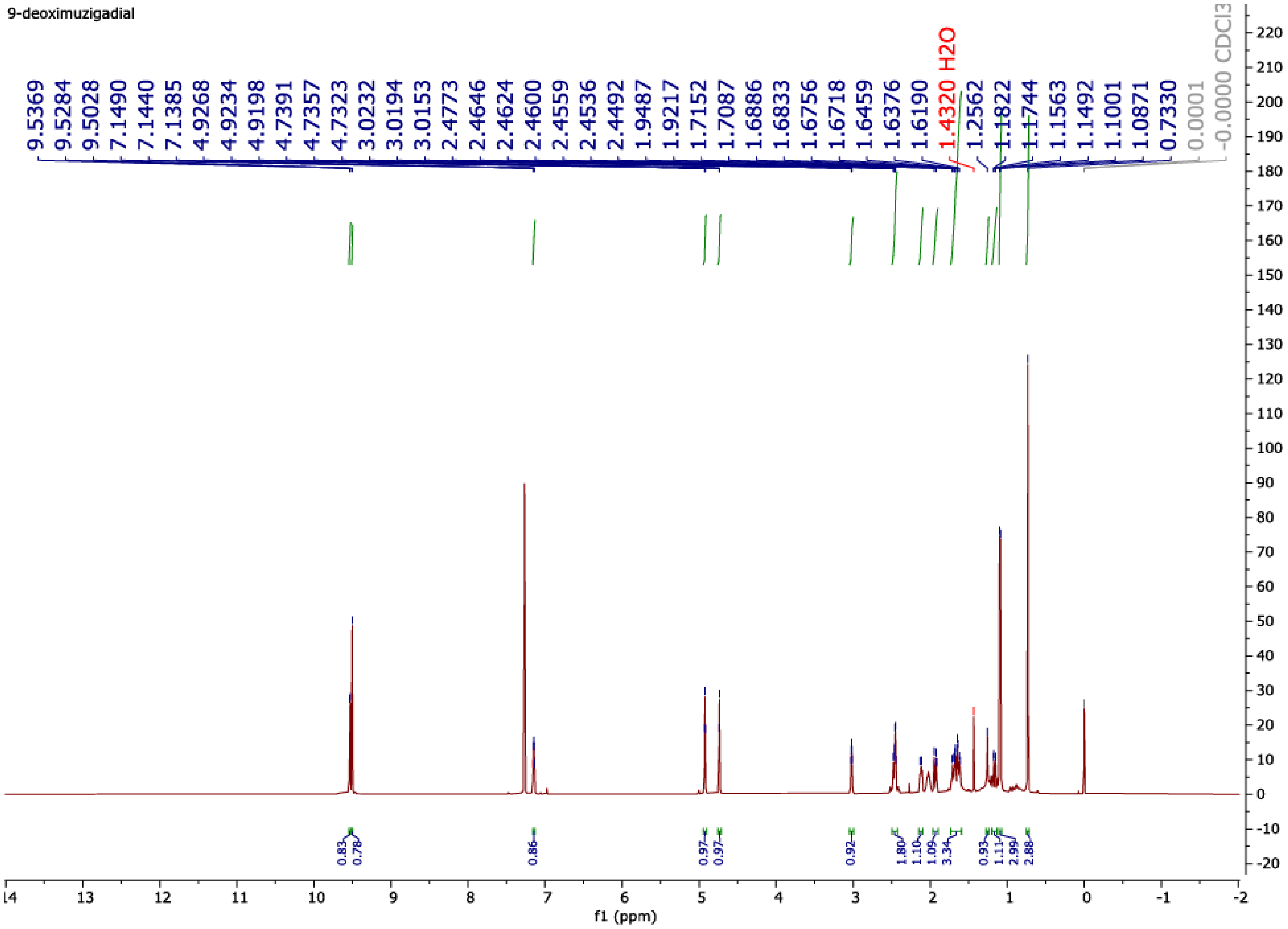

Figure S40. ^{1}H NMR spectrum of compound **6** (δ, $CDCl_3$, 500 MHz)

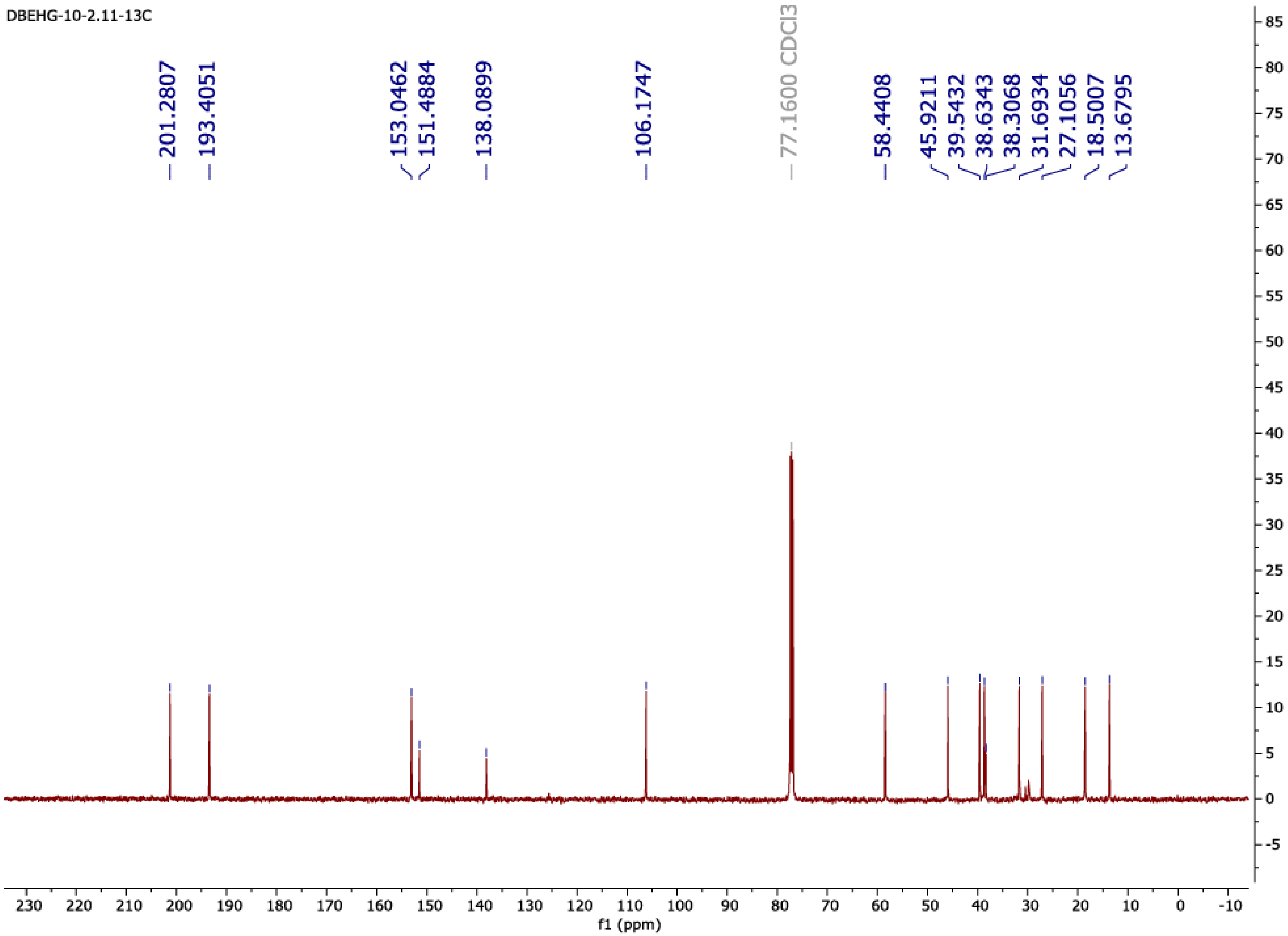

Figure S41. ^{13}C NMR spectrum of compound **6** (δ, $CDCl_3$, 125 MHz)

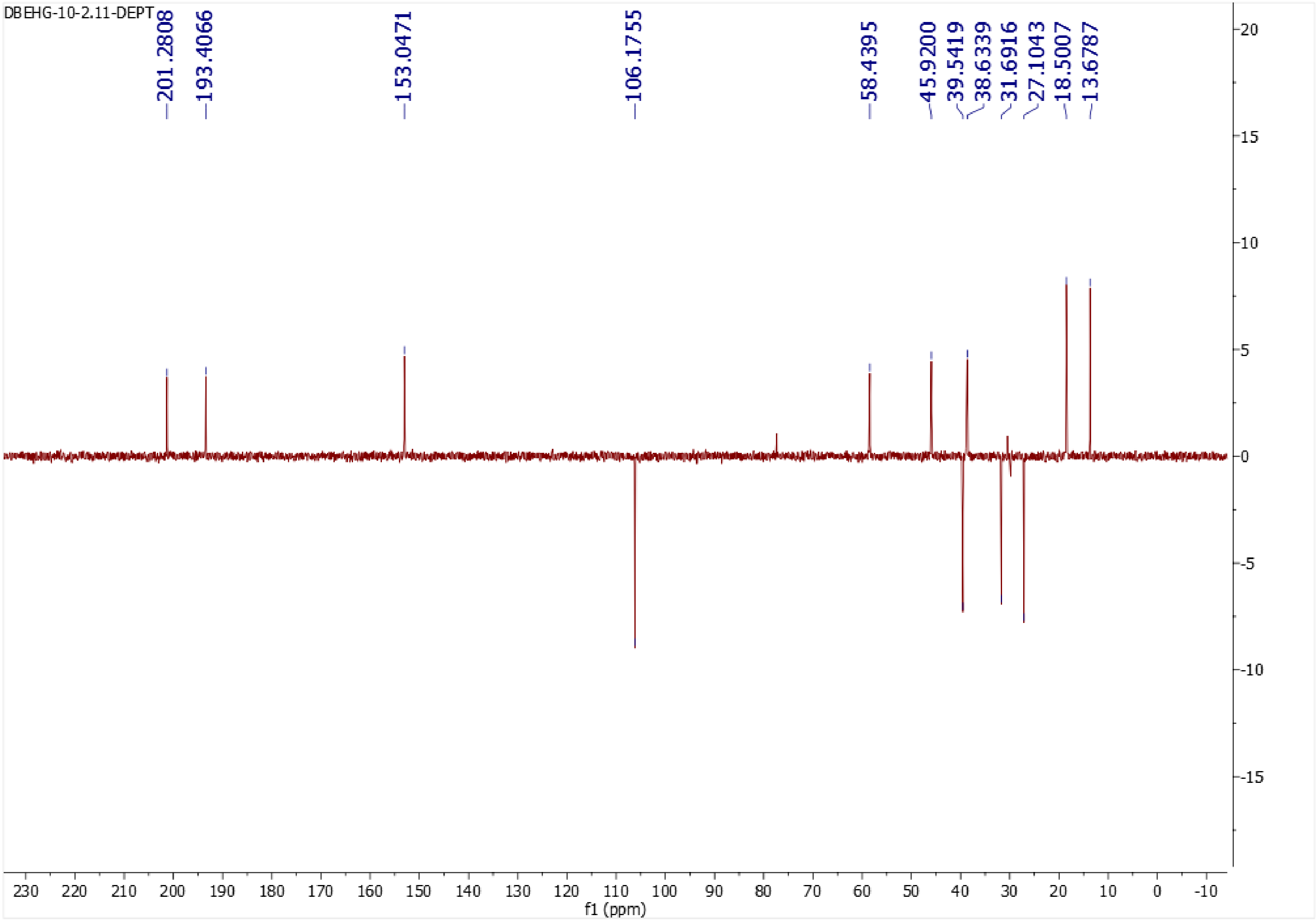

Figure S42. DEPT Spectrum of compound **6** (δ, $CDCl_3$, 125 MHz)

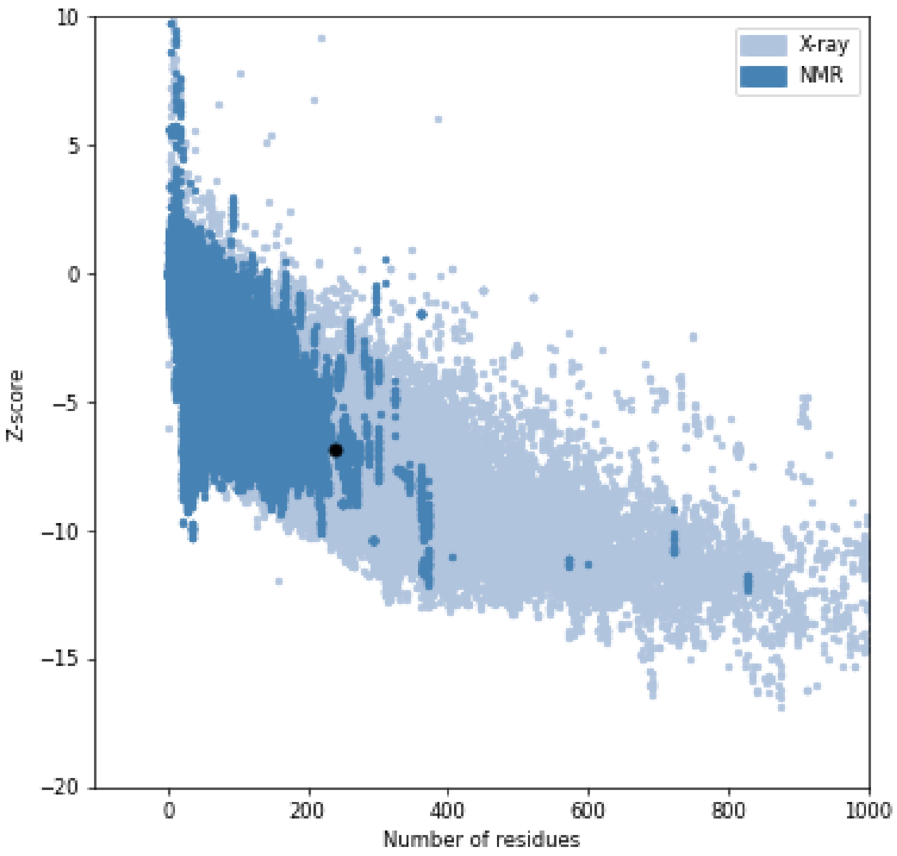

Figure S43. Z-score plot of the modeled *T. cruzi* ATP synthase β-subunit generated using ProSA-web.

The Z-score falls within the range of experimentally determined structures of comparable size, indicating good model quality.
